# Comorbidity between depression and anxiety: assessing the role of bridge mental states in dynamic psychological networks

**DOI:** 10.1186/s12916-020-01738-z

**Published:** 2020-09-29

**Authors:** Robin N. Groen, Oisín Ryan, Johanna T. W. Wigman, Harriëtte Riese, Brenda W. J. H. Penninx, Erik J. Giltay, Marieke Wichers, Catharina A. Hartman

**Affiliations:** 1 Department of Psychiatry, Interdisciplinary Center for Psychopathology and Emotion regulation (ICPE), University Medical Center Groningen (UMCG), University of Groningen, PO Box 30.001, 9700 RB Groningen, the Netherlands; 2 Department of Methodology and Statistics, Faculty of Social and Behavioural Sciences, Utrecht University, Utrecht, the Netherlands; 3 Department of Psychiatry, Amsterdam UMC, Vrije Universiteit and GGZ inGeest, Amsterdam, the Netherlands; 4 Department of Psychiatry, Leiden University Medical Center, Leiden, the Netherlands

**Keywords:** Network analysis, Comorbidity, Depression, Anxiety, Bridge symptoms, Time series, Intensive longitudinal data, Ecological momentary assessment

## Abstract

**Background:**

Comorbidity between depressive and anxiety disorders is common. A hypothesis of the network perspective on psychopathology is that comorbidity arises due to the interplay of symptoms shared by both disorders, with overlapping symptoms acting as so-called *bridges*, funneling symptom activation between symptom clusters of each disorder. This study investigated this hypothesis by testing whether (i) two *overlapping* mental states “worrying” and “feeling irritated” functioned as bridges in dynamic mental state networks of individuals with both depression and anxiety as compared to individuals with either disorder alone, and (ii) overlapping or non-overlapping mental states functioned as stronger bridges.

**Methods:**

Data come from the Netherlands Study of Depression and Anxiety (NESDA). A total of 143 participants met criteria for comorbid depression and anxiety (65%), 40 participants for depression-only (18.2%), and 37 for anxiety-only (16.8%) during any NESDA wave. Participants completed momentary assessments of symptoms (i.e., mental states) of depression and anxiety, five times a day, for 2 weeks (14,185 assessments). First, dynamics between mental states were modeled with a multilevel vector autoregressive model, using Bayesian estimation. Summed average lagged indirect effects through the hypothesized bridge mental states were compared between groups. Second, we evaluated the role of all mental states as potential bridge mental states.

**Results:**

While the summed indirect effect for the bridge mental state “worrying” was larger in the comorbid group compared to the single disorder groups, differences between groups were not statistically significant. The difference between groups became more pronounced when only examining individuals with recent diagnoses (< 6 months). However, the credible intervals of the difference scores remained wide. In the second analysis, a non-overlapping item (“feeling down”) acted as the strongest bridge mental state in both the comorbid and anxiety-only groups.

**Conclusions:**

This study empirically examined a prominent network-approach hypothesis for the first time using longitudinal data. No support was found for overlapping mental states “worrying” and “feeling irritable” functioning as bridge mental states in individuals vulnerable for comorbid depression and anxiety. Potentially, bridge mental state activity can only be observed during acute symptomatology. If so, these may present as interesting targets in treatment, but not prevention. This requires further investigation.

## Background

Comorbidity between depressive and anxiety disorders is common. For instance, of the individuals with a primary depression diagnosis in the Netherlands Study of Depression and Anxiety (NESDA), 67% had a current and 75% had a lifetime comorbid anxiety disorder diagnosis [1]. Similarly, of those with a primary anxiety disorder diagnosis, 63% had a current and 81% a lifetime depressive disorder diagnosis [1]. Although comorbidity rates in community samples are slightly lower [2, 3], the rates reported in NESDA are in line with other studies in primary and clinical settings [4, 5]. Comorbidity is consistently associated with higher illness severity, chronicity, and impairments in everyday life [4, 6, 7]. It is well-known that the presence of one of the disorders acts as a risk factor for developing the other [3, 8, 9]. This holds not only on a disorder level but also on a symptom level, with a meta-analysis by Jacobson and Newman [9] showing that symptoms of depression and anxiety predicted each other over weeks and months. Experiencing symptoms of one of these disorders may thus be associated with a higher risk of onset of symptoms of the second disorder.

**Fig. 1:**
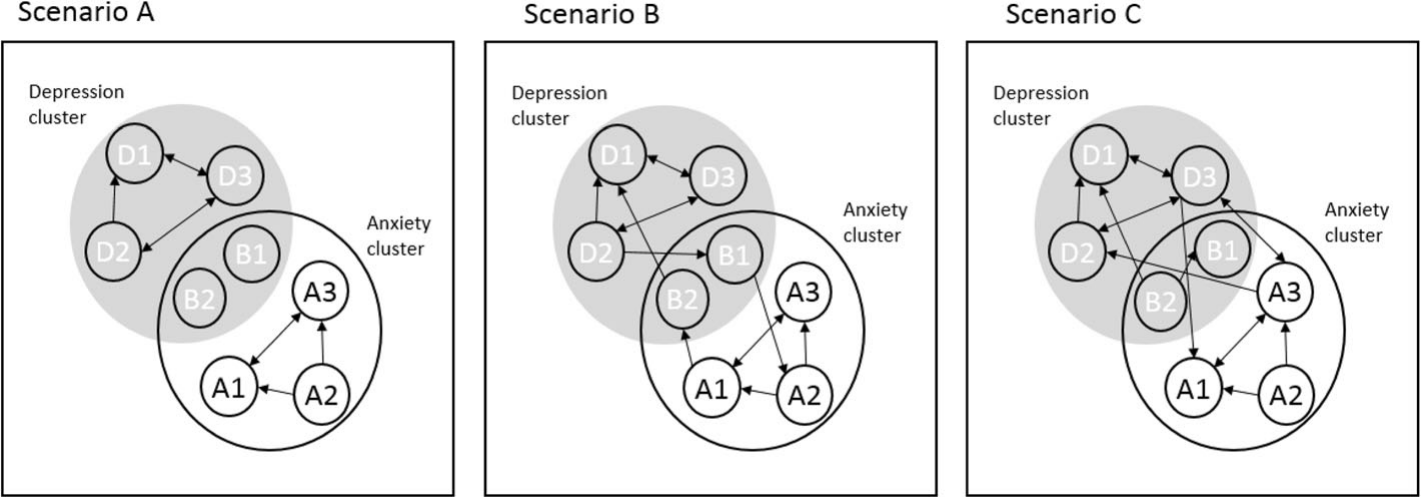
Network account of comorbidity. The small circles represent different symptoms, and the edges between them represent uni- or bidirectional associations over time. The larger circles represent clusters of symptoms of different diagnostic syndromes. The two symptoms (i.e., B1 and B2), which are part of both larger circles, are *overlapping* symptoms. These are common to both diagnostic syndromes. In scenario A, the overlapping symptoms are not activated as part of either diagnostic syndrome; therefore, no comorbidity between depression and anxiety arises. In scenario B, *overlapping* symptoms function as bridge symptoms between both diagnostic syndromes, resulting in comorbidity. In scenario C, comorbidity arises due to non-overlapping symptoms (i.e., D3 and A1) functioning as bridges between diagnostic syndromes

A heightened risk of developing symptoms of a second disorder can be understood from a network perspective on psychopathology, in which causal interactions between symptoms are theorized to drive the development of psychopathology including comorbidity [10]. Central to the network perspective is the proposition that psychiatric disorders arise due to symptoms triggering other symptoms over time, such as when, for instance, feeling listless makes it difficult to become active during the day, which later in the day results in increased sadness and restlessness because a person did not accomplish what he/she intended to do. Hypothetically, the stronger the depression symptoms from the above example trigger one another over time (i.e., the higher the network density), the more difficult it might be to disrupt the activation which eventually may result in the onset of a depressive disorder. This perspective also offers some potential explanations of comorbidity. For example, we can consider both anxiety and depression symptoms as subclusters in an overall network of psychopathology, including some symptoms that are shared between both disorders, the so-called overlapping symptoms. An interest in overlapping symptoms to explain comorbidity rates is not new (e.g., [11]). However, whereas previously it was examined whether comorbidity rates could be attributed to the presence of these symptoms ([12, 13] found no support, but see [14] also), in the network approach, these symptoms are hypothesized to play an active role in the development of comorbidity [10, 15]. If there are no direct interactions between anxiety and depression symptoms (as depicted in Fig. 1 (a)), we would expect there to be no comorbidity of both disorders. If such symptoms, like worrying [16] or irritability [17], share strong relationships with symptoms from each individual disorder, then the symptoms of disorder A may activate the symptoms of disorder B indirectly through these symptoms (as depicted in Fig. 1 (b)). An alternative view posits that any symptom, so also symptoms that are specific to only one of the two disorders, could potentially act as a bridge between disorder (e.g., [18, 19]). This is depicted in Fig. 1 (c), where symptom activation from the depression-cluster to the anxiety-cluster via non-overlapping anxiety and depression symptoms would too lead to comorbidity.

Multiple cross-sectional network studies have addressed comorbidity between depressive and anxiety disorders [10, 16–26]. In these studies, symptoms of both diagnoses were combined into one network structure, with symptom connections reflecting the statistical relationships between symptoms when the value of all other symptoms variables is controlled for. Symptoms belonging to the same disorder tended to be more strongly connected to symptoms of the same disorder than to symptoms of the other, comorbid disorder ([15, 16, 18–26], but see [10] as exception). Each study identified at least one symptom in the network that connected both to symptoms of the same disorder and to the symptom cluster belonging to the second, comorbid disorder. However, these symptoms were not always overlapping symptoms (e.g., [18, 19]). Irrespective of which bridge symptom conceptualization (i.e., overlapping or non-overlapping symptoms) was used, the structures of cross-sectional networks revealed patterns that we might expect based on the bridge symptom hypothesis.

However, cross-sectional network patterns may not reflect symptom activation within a person over time [27–29]. The use of intra-individual time series to model network structures has two immediate advantages over the use of cross-sectional data: first, patterns of dependence within-person can be directly established, avoiding well-known problems with ergodicity [27–29], and second, time-series analysis allows us to establish *time-lagged* (i.e., temporal) dependencies between symptoms often discussed as a necessary though not sufficient condition for the presence of a causal relationship [30]. Temporal networks based on *simulated* intra-individual time series in which comorbidity could only arise via bridge symptoms showed promising results [31], in that they reproduced prevalence rates of major depressive disorder (MDD) and generalized anxiety disorder (GAD), and their comorbidity as reported by the National Comorbidity Survey Replication (NCS-R [32]). The bridge symptom hypothesis has not been investigated yet using *empirical* time-series data. A next step would thus be to investigate whether the expected temporal pattern is observed in networks based on intra-individual time series of individuals diagnosed with comorbid depression and anxiety. To this end, ecological momentary assessment (EMA) designs could be used, in which momentary experiences of symptoms are assessed by asking participants’ multiple days, and multiple times per day to report on their current mental state (e.g., feeling sad or anxious). Although these mental states map onto symptoms listed in diagnostic criteria of depressive and anxiety disorders, and could therefore be considered as daily proxies of symptoms, they are not symptoms. For the remainder of this paper, we will refer to such momentary experiences of symptoms and affect assessed with EMA as “mental states.”

**Fig. 2:**
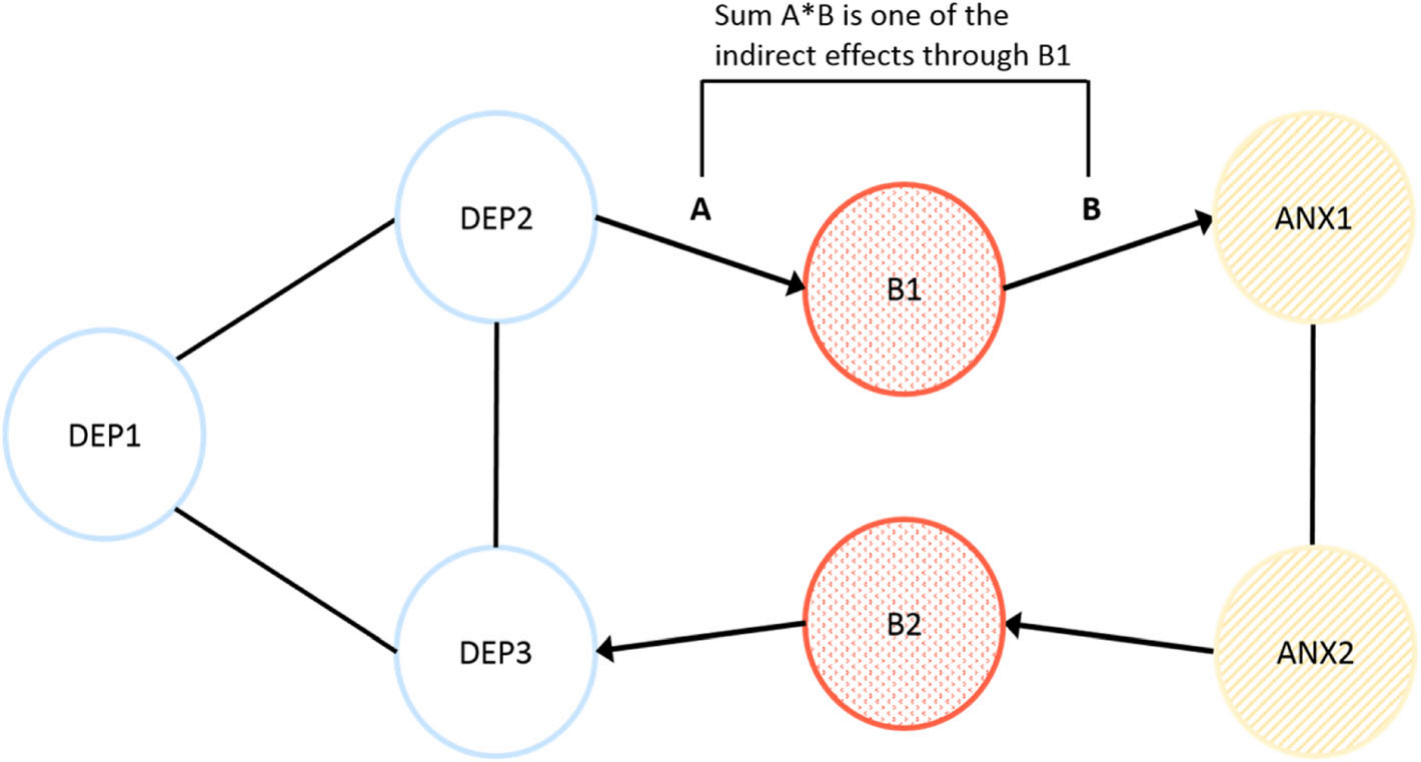
Hypothetical network of comorbid depression (DEP1–DEP3) and anxiety (ANX1 and ANX2), in which bridge mental states (i.e., momentary experiences of symptoms) (B1 and B2) funnel the activity from one mental state cluster to the other. The lines within the two mental state clusters show the within-cluster associations between the mental states. The four arrows show possible indirect effects from depression mental state 1 (DEP1) → bridge mental state 1 (B1) → anxiety mental state 1 (ANX1), and vice versa, from anxiety mental state 2 → bridge mental state 2 → depression mental state 3. The indirect effects via the bridge mental states, such as A*B in the figure, are hypothesized to be stronger in the network of individuals with comorbid depression and anxiety, as compared to individuals with either disorder

In the current paper, we investigate the bridge symptom hypothesis using EMA data. Investigating whether we can detect within-individual temporal associations corresponding with this hypothesis is novel. We used data from participants in the Netherlands Study of Depression and Anxiety [33], who had been diagnosed with depression, anxiety, or both, and reported on multiple depression and anxiety mental states, multiple times a day, for 2 weeks. As such, this dataset provides the opportunity to study whether bridge mental states are stronger in the within-day mental state dynamics in individuals with comorbid depression and anxiety to those with either single disorder. A priori, we hypothesized that two *overlapping* mental states “feeling irritated” and “worrying,” reflecting momentary expressions of shared symptoms (i.e., irritability and repetitive negative thinking) of depression and anxiety, would function as bridge mental states. In this paper, we consider a mental state to be a “bridge” between two clusters of mental states from different syndromes if indirect effects from depression mental states to anxiety mental states and/or vice versa travel via this mental state (Fig. 2). The degree to which a symptom acts as a bridge can thus be quantified by the strength of all those indirect effects. Our first aim was to test whether overlapping mental states functioned as stronger “bridges” in the network of individuals with comorbid disorders as compared to individuals with single disorders. Our second aim was to evaluate the assumption that overlapping mental states are more likely to act as bridge mental states than are non-overlapping mental states [15]. In the “Discussion” section of this paper, we synthesize our results, identify and elaborate on the barriers to using this type of analytic approach to investigate causal relationships between mental states, and suggest multiple areas for future research.

## Methods

### Preregistration

The aims and analyses of the current paper were preregistered on the Open Science Framework (https://osf.io/jwuz9). When deviations from the preregistered analyses occurred, reasons for this are explained in the footnotes. More detail about the preregistration, deviations, and sensitivity analyses can be found in Additional File 1 [34–39].

### Sample selection

The current study used data collected in a subsample of participants that completed the Ecological Momentary Assessment & Actigraphy add-on study of the Netherlands Study of Depression and Anxiety (NESDA-EMAA). NESDA is an ongoing multisite longitudinal cohort study of Dutch adults (at baseline: *n* = 2981; age 18–65 years), with and without depressive and/or anxiety disorders who were recruited from community settings, and primary and secondary healthcare settings. Participants were excluded if they (i) had a primary diagnosis of psychotic, obsessive-compulsive, bipolar, or severe addiction disorder or (ii) were not fluent in Dutch. See [33] for a detailed description of the rationale, objectives, and methods of NESDA. Six measurement waves, including the baseline measurement, have so far been completed in NESDA.

At the sixth measurement wave (2014–2017), NESDA-EMAA took place. Inclusion criteria of this add-on study were: participation in at least two previous NESDA waves, participation in the regular interview ≤ 31 days prior to starting the EMA measurements, and being familiar with smartphone use. Based on participants’ diagnostic history (derived from diagnostic interviews conducted at each NESDA wave), we selected NESDA-EMAA participants (*n* = 220) that had > 30%^1^ completed EMA assessments and could be allocated to one of the following three groups: (i) comorbid depression and anxiety at any NESDA measurement wave (*n* = 143), (ii) only depression at any NESDA measurement wave (*n* = 40), and (iii) only anxiety at any NESDA measurement wave (*n* = 37). Participants were assigned to the comorbidity group if they met criteria for both a depressive disorder (MDD or dysthymia) and an anxiety disorder (generalized anxiety disorder (GAD), panic disorder with (out) agoraphobia, agoraphobia, or social phobia) at the most recent or any prior NESDA wave. The episodes of depression or anxiety had to occur at the same NESDA wave. Individuals diagnosed with depression and anxiety at different waves were not included as part of the comorbidity group. Participants who met criteria for a depressive disorder at any NESDA wave and did not meet criteria for an anxiety disorder at any NESDA wave became the depression-only group. Likewise, participants were assigned to the anxiety-only group if they met criteria for an anxiety disorder at any NESDA wave, but did not meet criteria for a depressive disorder at any NESDA wave. However, some of the participants (*n* = 15; 40.5%) in the anxiety-only group met criteria for a depressive disorder prior to NESDA (> 9 years ago), see page 2 in Additional File 1, for further details on group allocation.

For a more detailed description of the NESDA-EMAA recruitment procedure and inclusion criteria, see [40]. The research protocols of both the main NESDA study and EMAA add-on study were approved by the Ethical Review Board of the VU University Medical Center and thereafter by the local ethical committees of the participating centers. All participants gave verbal and written informed consent.

### Design

NESDA-EMAA participants were asked to complete five EMA questionnaires a day according to a fixed sampling scheme with 3-h intervals, for 2 weeks. Questions concerned participants’ current mental state, activities, and company and took between 3 and 5 min to complete. During each day, at the appointed times, participants received a text message on their smartphones with a link to an online EMA questionnaire. Participants had to complete the questionnaire within 60 min after the prompt, but were encouraged to do so as soon as possible (preferably within 15 min of the prompt). The questionnaires were administered and stored via Roqua (www.roqua.nl). If participants answered at least 80% of all diary questions, they received a 20-euro gift-card as remuneration as well as a personalized report of their own EMA responses. The EMA protocol has been described in detail elsewhere [41].

### Measurements

#### Ecological momentary assessments

Of the 16 questions on current mental states (positive and negative affect) and cognitions (see [40] for a complete list), we selected seven questions,^2^ which most directly reflect depression, anxiety, and bridge mental states. Items were translated from Dutch. The three selected depression mental states were as follows: “I feel listless/apathetic” (LIS; “Ik voel me lusteloos), “I feel down” (DOW; “Ik voel me somber”), and “I do not feel cheerful” (CHE; reverse coded from “Ik voel me opgewekt”). The two anxiety items were as follows: “I feel nervous” (NER; “Ik voel me nerveus”) and “I do not feel relaxed” (REL; reverse coded from “Ik voel me ontspannen”). The two bridge mental states were as follows: “I worry/brood a lot” (WOR; “Ik pieker veel”) and “I feel irritated” (IRR; “Ik voel me geïrriteerd”). Participants were asked to rate the extent to which they experienced each mental state at the moment of assessment on a 7-point Likert scale ranging from 1 = “not at all” to 7 = “very.”

#### Diagnostic interview

The Composite International Diagnostic Interview (CIDI, lifetime version 2.1 [42]) was used to assess the presence of depressive disorders (major depressive disorder and dysthymia) and anxiety disorders (generalized anxiety disorder, panic disorder, agoraphobia, social phobia) at each NESDA wave, in between NESDA waves, and before enrollment in NESDA.

#### Symptom severity

Current (past week) severity of depressive and anxiety symptoms was assessed using the Inventory of Depressive Symptomatology-Self Report (IDS-SR30 [43]), the Beck Anxiety Inventory (BAI [44]), and Fear Questionnaire (FQ [45]), respectively.

### Statistical analysis

Analyses were conducted in Mplus version 8.3 [46] and in R version 3.6.2 [47]. using packages *qgraph* [48] for network visualization and *MplusAutomation* [49] for reading Mplus output into R. All R code and Mplus syntax can be found on OSF (https://osf.io/jzru8/) and the Mplus syntax of the main analysis in Additional File 2.

#### Cross-lagged associations (dynamic mental state networks)

To investigate the dynamic associations between our seven momentary mental states, we fitted a multilevel multivariate first-order vector autoregressive model (VAR(1) ) using the Dynamic Structural Equation Model (DSEM) module in Mplus [38]. There was no multicollinearity among predictors (see Additional file 1: Table S3); therefore, all seven items were included in the model. Moreover, visual inspection of participants’ responses over time revealed no initial elevation bias [50]; therefore, we included all completed assessments in our model. We evaluated the VAR model assumption of stationarity, by visual inspection and by means of the Kwiatkowksi-Phillips-Schmidt-Shin unit root test (KPSS [51]). We observed one individual with a clear trend; however, when running our models without this individual, we did not find results to differ and did not exclude this individual. The KPSS test indicated that time series were stationary for the majority of participants (71–91% depending on the variable); we therefore did not linearly detrend the data.^3^

Within DSEM, the total variability across individuals and across time points is decomposed into two components: within-person variability and between-person variability. Each of these parts is modeled separately [36]. For the within-person part, previous values (at *t-1*) of the mental state variables were used to predict mental state variables at time *t*. In our case, the majority of assessments were approximately 3 h apart (median = 183 min, IQR = 175–225 min). However, larger intervals also occurred due to the sampling procedure (no nighttime assessments) and missed assessments. One important assumption of VAR models is that time series are equally spaced. This is because the size of the interval between assessments influences the strength of the estimated autoregressive and cross-lagged effects [52–55]. To account for the unequally spaced intervals, we used the TINTERVAL command in Mplus such that parameter estimates should be taken to reflect lagged relationships at an approximately 180-min^4^ time-interval [38].

At the between level, we modeled the effect of group membership using dummy variables on the latent variables that we obtained through the within-level model (i.e., the autoregressive and cross-lagged parameters from the within-person level become latent variables at the between-person level). This allowed us to obtain point estimates for the average autoregressive and cross-lagged parameters in each group. Subsequently, we used the average group cross-lagged effects to calculate lag-2 indirect effects (see Fig. 2) for each group to test our two research questions (explained in detail below) [56].

We used the default specifications of DSEM, which performs Bayesian estimation with non-informative priors based on two independent Monte Carlo Markov Chains (MCMC) [38]. Bayesian estimation has several advantages for the estimation and group comparison of the indirect effects that were of interest in this study. First, the hierarchical model of interest and subject-specific parameters model can be directly estimated (rather than relying on indirect marginal model specification as in maximum likelihood-based methods). Second, the use of MCMC sampling means that we can obtain posterior distributions for each of the bridge effects reported in this paper, by simply recording at every iteration the current estimate of each within-person lagged parameter. This procedure provides both point estimates and uncertainty estimates (in the form of credible intervals) which are computed from the posterior distribution. Using frequentist approaches, it would be difficult to obtain standard errors and *p* values of the same effects. Third, DSEM allows for fitting a single multivariate model, while separate models for each outcome variable would have been necessary using a frequentist approach. Lastly, the presence of missing observations and unequal time-intervals are handled differently in DSEM as compared to how they would be dealt with in frequentist approaches. Namely, during estimation of multilevel VAR models using maximum likelihood, missing observations are typically deleted, and unequal time-intervals are typically ignored, likely leading to biased parameter estimates [55]. In contrast, in DSEM, missing data are automatically “imputed” as they are sampled from a posterior distribution during each MCMC iteration (i.e., resembling a Kalman filter approach, see [35]). When combined with the TINTERVAL option (which inserts missing values such that observations are approximately equally spaced), the bias we typically introduce by ignoring unequal time-intervals is avoided. For a more detailed discussion of the differences between DSEM and frequentist approaches, see Mcneish and Hamaker [57] and Hamaker et al. [35].

From the estimated posterior distribution for each indirect effect, we obtained a point estimate and Bayesian credible interval (based on the mean and spread of the posterior, respectively), which quantified the uncertainty about each effect. We use the credible interval as a pseudo-significance test (with parameters labeled as “significant” if their 95% CI does not include zero). More information on the preprocessing steps, treatment of missing data, and DSEM specifications can be found in Additional File 1 [35–37].

### Research question 1: Do overlapping mental states function as stronger bridge mental states in the comorbid group as compared to the single disorder groups?

To test the hypothesis that overlapping mental states functioned as stronger bridge mental states in the network of participants with comorbidity as compared to participants with a single disorder, we first estimated all average within-person indirect effects through both overlapping mental states (“feeling irritated” and “worrying”) for each group separately. These indirect effects represent the overall effect from one cluster to another cluster via the potential bridge mental state. They were estimated as the cross-product of the group-specific fixed effects for lagged associations from an anxious mental state to a bridge state and from a bridge to a depressive state, and vice versa (see Fig. 2 for illustration), and thus reflect the average within-person indirect effect in that group. We then summed these indirect effects, obtaining a group-average summary of all indirect effects through a potential bridge mental state. For the remainder of this paper, we will call this summed indirect effect the “bridge effect,” as this measure quantifies the degree to which the variable acts as a bridge. We subtracted the value of each bridge effect in the anxiety-only and depression-only groups from those of the comorbid group. This resulted in four difference scores (two for both overlapping mental states). To determine whether the estimates (i.e., bridge effects and difference scores) were different from zero, we inspected whether zero fell into the 95% credible intervals of the respective posterior distributions.

### Research question 2: Are overlapping mental states the best bridge mental states?

We examined whether the overlapping mental states “worrying” and “feeling irritated” were most likely to act as bridge mental states, by evaluating whether their bridge effects were higher than bridge effects of the non-overlapping mental states. To this end, we computed the bridge effect for all mental states in the network. Because “worrying” and “feeling irritated” are part of both the anxiety and depression mental state clusters, they could represent either anxiety or depression mental states in calculating the indirect effects. For instance, in case “feeling down” was treated as bridge mental state, both the indirect effect of “worrying” → “feeling down” → “feeling listless” (i.e., from anxiety to depressive mental state via the bridge) and “worrying” → “feeling down” → “feeling nervous” (i.e., from depressive to anxiety mental state via the bridge) would be part of the bridge effect for “feeling down.” However, we did not count an effect from “worrying” → “feeling down” → “worrying,” as indirect effects had to begin and end at a different node. Due to unequal numbers of items belonging to the depression (3 items) and anxiety (2 items) mental state clusters, different numbers of indirect effects contributed to the bridge effects. For the “original bridge states” (“worrying” and “feeling down”), the bridge effect consisted of 22 indirect effects, while this was 24 when the anxiety mental states were conceptualized as bridge state, and 26 in case any of the depressive mental states was considered as bridge state. Due to the differences in number of indirect effects contributing to the bridge effect, we report the mean bridge effect for each node and ranked nodes accordingly. The position of “worrying” and “feeling irritated” among that ranking was evaluated descriptively.

#### Sensitivity analyses

We performed two sensitivity analyses to evaluate to what extent findings among the three groups were influenced by (i) anxiety and depression severity differences and (ii) recency of diagnoses.^5^ In the first sensitivity analysis, we compared the single disorder groups to participants (*n* = 46) in the lowest severity tertile of the comorbid group. Having comorbid disorders is associated with higher severity of complaints [1]. Severity differences could drive possible group differences in dynamic structure, because especially lower levels of severity may coincide with low levels of variability (due to restriction of range [58]) which in turn may influence the cross-lagged estimates. In the second analysis, we compared the single disorder groups to participants (*n* = 75) of the comorbid group with a current diagnosis (i.e., diagnosis in the 6 months prior to the EMA study). This sensitivity analysis was done post hoc and aimed to evaluate whether the contrast between groups in terms of bridge effects would become more pronounced.

## Results

Together, the 220 participants completed 14,185 observations with on average 64.48 (SD = 6.16, range 24–70) observations per participant. Diagnostic groups did not differ in sex, age, or the number of EMA assessments completed. However, there were differences in level of education, symptom severity, number of diagnoses, and recency of diagnoses (Table 1). Groups also differed in intra-individual means for all variables and intra-individual standard deviations for some of the mental state variables. Table 2 presents these results. Overall, the findings from Tables 1 and 2 indicate that the comorbid group was worse off compared to the single disorder groups. Participants with a depression diagnosis appeared to be slightly better off than participants with an anxiety diagnosis, although differences were not statistically significant.

**Table 1:** Demographics and clinical characteristics compared between the three outcome groups

	1. Comorbid disorders at the same NESDA wave (*n* = 143)	2. Depressive disorder only during NESDA (*n* = 40)	3. Anxiety disorder only during NESDA (*n* = 37)	*p* value
**Demographics**				
Female, *n* (%)^a^	100 (69.9)	29 (72.5)	27 (73.0)	0.91
Age, mean (SD)^b^	48.67 (11.1)	47.38 (14.1)	48.62 (13.3)	0.83
Education, *n* (%)^c^				0.08
1. *Low*	8 (5.6)	1 (2.5)	0 (0)	
2. *Intermediate*	84 (58.7)	17 (42.5)	17 (45.9)	
3. *High*	51 (35.7)	22 (55.0)	20 (54.1)	
Born outside the Netherlands (%)^c^	8 (5.6)	1 (2.5)	4 (10.8)	0.33
Marital status (%)^c^				0.84
Never married	46 (32.2)	12 (30.0)	15 (40.5)	
Currently married	64 (44.8)	21 (52.5)	17 (45.9)	
Married living separated	2 (1.4)	0 (0)	0 (0)	
Formerly married	31 (21.7)	7 (17.5)	5 (13.5)	
Employment status—unemployed (%)^a^	33 (22.4)	3 (7.5)	3 (8.1)	**0.03**
**Psychopathology**				
Type of disorder				
Major depression (%)	143 (100.0)	38 (95)	0	
Dysthymia (%)	67 (48.9)	7 (18.4)	0	
Generalized anxiety disorder (GAD) (%)	89 (64.0)	0	12 (33.3)	
Social anxiety disorder (%)	93 (66.4)	0	23 (65.7)	
Panic with/without agoraphobia (%)	89 (63.3)	0	15(41.7)	
Agoraphobia (%)	52 (38.2)	0	8 (22.2)	
Recency* (%)^a^				**< 0.001**
< 2 weeks	54 (37.8)	1(2.5)	8 (21.6)	
2 weeks to < 1 month	11 (7.7)	0	1 (2.7)	
1 to < 6 months	10 (6.9)	6 (17.5)	3 (32.4)	
6 to 12 months	7 (4.9)	2 (5.0)	1 (2.7)	
> 1 to 9 years^#^	61 (42.0)	31 (77.5)	24 (64.9)	
Symptom severity mean (SD)^b^				
Inventory of Depressive Symptoms (IDS; range 0–84), mean (SD)^b^	22.4^2,3^ ± 12.7	13.0^1^ ± 9.4	11.3^1^ ± 5.9	**< 0.001**
Beck Anxiety Inventory (BAI; range 0–63), mean (SD)^b^	11.7^2,3^ ± 9.7	5.3^1^ ± 5.8	7.0^1^ ± 5.4	**< 0.001**
Fear Questionnaire (FQ; range 0–120), mean (SD)^b^	21.7^2,3^ ± 19.0	8.3^1^ ± 11.7	15.3^1^ ± 13.2	**< 0.001**

^a^Chi-squared test

^b^Analysis of variance (ANOVA)

^c^Fisher’s exact test

^1,2,3^Numbers refer to groups from which that group differs significantly

*Established at the time of the interview; the EMA study started within 31 days of the interview

^#^Number of individuals who only met criteria for a diagnosis at the first NESDA measurement; N=5 in comorbid group; N=10 in depression group; and N=12 in anxiety group

**Table 2:** Descriptives (prevalence of items, person means, within-person standard deviation) of the momentary mental states

	Comorbidity group			Depression-only			Anxiety-only		
	Percent endorsed (score of 2 or higher)^a^	Person mean (SD)^b^	Within-person variation (SD)^a^	Percent endorsed (score of 2 or higher)^a^	Person mean (SD)^b^	Within-person variation (SD)^a^	Percent endorsed (score of 2 or higher)^a^	Person mean (SD)^b^	Within-person variation (SD)^a^
Not relaxed	96.6^2,3^	3.52 (0.73)^2,3^	1.10 (0.28)	87.8^1,3^	2.84 (0.69)^1^	1.13 (0.31)	92.2^1,2^	3.09 (0.71)^1^	1.10 (0.33)
Not cheerful	98.2^2,3^	3.66 (0.86)^2,3^	0.97 (0.29)	93.9^1^	3.03 (0.71)^1^	0.95 (0.33)	94.2^1^	3.01 (0.70)^1^	0.89 (0.33)
Irritated	49.3^2^	2.09 (0.89)^2,3^	1.07 (0.40)	22.3^1^	1.48 (0.47)^1,3^	0.88 (0.42)	36.8	1.70 (0.51)^1,2^	1.00 (0.40)
Listless	60.4^2,3^	2.48 (1.07)^2,3^	1.04 (0.38)	30.7^1^	1.75 (0.96)^1^	0.87 (0.48)	36.7^1^	1.72 (0.61)^1^	0.92 (0.46)
Down	58.2^2,3^	2.43 (1.21)^2,3^	0.94 (0.38)^3^	24.1^1^	1.50 (0.63)^1^	0.70 (0.48)	28.4^1^	1.47 (0.51)^1^	0.65 (0.44)^1^
Nervous	54.0^2,3^	2.22 (1.05)^2,3^	0.96 (0.39)^2^	18.9^1,3^	1.37 (0.55)^1,3^	0.58 (0.43)^1,3^	39.8^1,2^	1.77 (0.74)^1,2^	0.88 (0.49)^2^
Anxious	34.1^2,3^	1.70 ± (0.97)^2,3^	0.61 (0.41)^2^	7.0^1^	1.11 (0.26)^1,3^	0.23 (0.33)^1^	17.8^1^	1.27 (0.36)^1,2^	0.47 (0.40)
Worry	72.8^2,3^	3.02 (1.43)^2,3^	0.94 (0.39)	39.3^1^	1.76 (0.79)^1^	0.79 (0.47)	56.2^1^	2.19 (1.01)^1^	0.85 (0.46)

^a^Analysis of variance (ANOVA)

^b^Kruskal-Wallis test

^1,2,3^Numbers refer to groups from which that group differs significantly based on post hoc comparisons: Tukey’s HSD (in case of ANOVA) or Dunn’s test (in case of Kruskal-Wallis)

**Fig. 3:**
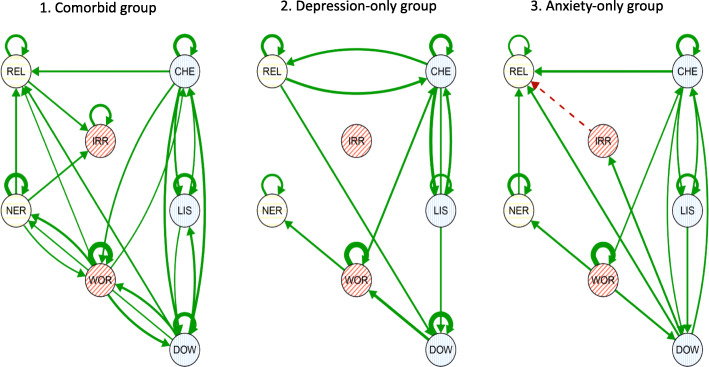
Networks depicting the group-average lagged association (fixed) effects between mental states of the VAR(1) model for the comorbid, depression-only, and anxiety-only groups. Each node in the network represents a mental state: REL, not relaxed; NER, feeling nervous; IRR, feeling irritated; WOR, worrying; CHE, not cheerful; LIS, feeling listless; DOW, feeling down. Each edge is the lagged association of that mental state at *t-1* (3 h before) with another mental state at *t* (now). Autoregressive effects (curved arrows from and to the same node) show the influence of the mental state on itself over time. Green (solid) arrows reflect positive effects over time, while red (dotted) arrows reflect negative effects over time

The estimated average VAR(1) networks for each of the three groups are displayed in Fig. 3 [59]. Here, group-average lagged regression parameters which were not significant (i.e., have a credible interval that contains zero) are omitted. Supplementary Tables 4 and 5 in Additional File 1 present all lagged mental state associations for each group and variances of random effects, respectively. The comorbid group’s network was denser (26 out of 49 edges) than the other two groups (depression-only, 14/49 edges; anxiety-only, 20/49 edges), and all effects were positive. Furthermore, the comorbid group’s network featured the most bidirectional associations between mental states of the three groups The largest effect across all groups was found in the anxiety-only group and concerned the AR effect for “worrying” (beta = 0.36 [95% CI 0.26, 0.41]). The largest cross-lagged effect was also found in the anxiety-only group and concerned the effect from “not cheerful” at *t-1* to “not relaxed” at *t* (beta = 0.13 [95% CI 0.06–0.19]). In both the comorbid group and the depression-only group, there were significant associations going from a depression mental state to the bridge mental state “worrying” to an anxiety mental state. In the comorbid group, the opposite direction (i.e., anxiety to worry to depression) was also observed. In all groups, “worrying” featured more significant effects than “feeling irritated.” In the depression-only group, there was no significant effect to or from the mental state “feeling irritated” at all.

### RQ 1: Do overlapping mental states function as stronger bridge mental states in the comorbid group as compared to the single disorder groups?

**Fig. 4:**
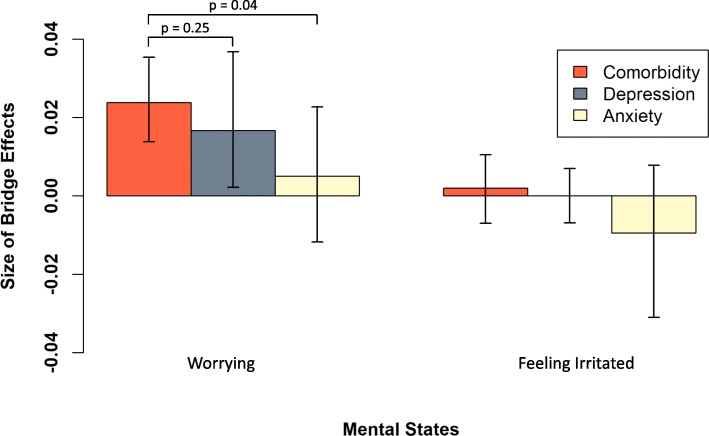
Average within-person summed indirect effects with credible intervals (black lines) going through the overlapping mental states “worrying” and “feeling irritated,” for the comorbid, depression-only, and anxiety-only groups

In Fig. 4, we have plotted the bridge effects of the overlapping mental states “worrying” and “feeling irritated” for each group. In the comorbid and depression-only groups, the bridge effect of “worrying” was significantly different from zero (effect comorbidity 0.024 [95% CI 0.014, 0.035], *p* < 0.001; effect depression-only 0.017 [95% CI 0.002, 0.037], *p* = 0.012), but this was not the case for the anxiety-only group (effect anxiety-only 0.005 [95% CI − 0.012, 0.023], *p* = 0.255). However, the CIs for the difference between the comorbid and depression-only groups (delta = 0.007 [95% CI − 0.015, 0.026], *p* = 0.251), and the difference between the comorbid and anxiety-only groups (delta = 0.019 [95% CI − 0.002, 0.039], *p* = 0.035) included zero. The bridge effect of “feeling irritated” was not significantly different from zero for any of the three groups, nor different from each other.

The Bayesian credible intervals (CIs) for the comorbid group are narrower than the CIs of the other two groups, likely due to the differences in group sizes; in the first sensitivity analysis that adjusted for the larger severity and sample size of the comorbid group, CIs of the comorbid group resembled CIs of the other groups (see Additional file 1: Fig. S5). Importantly, in this sensitivity analysis, the bridge effect of worrying for the lowest tertile of the comorbid group was of the same size as the effect for the depression-only group. The results of the second sensitivity analysis  (see Additional file 1: Fig. S6, which compared participants in the comorbid group with current diagnoses (< 6 months prior to EMA assessments) to the single disorder groups, showed a larger bridge effect for “worrying” in the comorbid group as compared to the main analysis. The difference in the bridge effect of worrying between the comorbid and single disorder groups became slightly larger (delta_com-dep_ = 0.012 [95% CI − 0.013, 0.035], *p* = 0.171; delta_com-anx_ = 0.024 [95% CI 0.002, 0.047], *p* = 0.017). Both sensitivity analyses yielded the same result for “feeling irritated” as the main analysis, that is, the bridge effect of “feeling irritated” was not significantly different from zero for any of the three groups, nor different from each other.

### RQ 2: Are overlapping mental states the best bridge mental states?

**Fig. 5:**
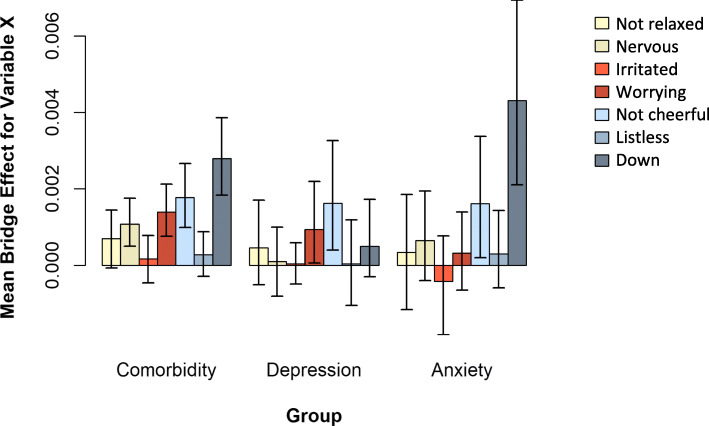
Average within-person summed indirect effects with credible intervals (black lines) for each mental state when treating that mental state as a bridge mental state, for the three groups separately

Figure 5 presents the mean bridge effect (i.e., bridge effect divided by number of indirect effects) of each mental state in each group. Within each group, we compared the mental state with the highest mean bridge effect to the other bridge mental states (see Table 3). Not “worrying” but “feeling down” featured the highest bridge effect in the comorbid group. With the exception of “not feeling cheerful,” “feeling down” was also significantly stronger than other mental states in this group. The same pattern was observed for the anxiety-only group. With the exception of “not feeling cheerful,” the mean bridge effect of “feeling down” was stronger than the bridge effects of all other mental states in this group (estimate = 0.0043 [95% CI 0.0021, 0.0069]). The mean bridge effect of “not feeling cheerful” was the only effect significantly different from zero in all three groups (but not different between groups as CIs overlapped considerably), indicating that this mental state funnels activity from depressed to anxiety mental states (and vice versa) irrespective of the diagnostic label. Again, the CIs in the comorbid group are generally smaller than those in the other groups. In all sensitivity analyses, the mean bridge effect of “feeling down” appeared to have the highest point estimate in the comorbid group and anxiety-only group (see Additional file 1: Fig. S6-S7). However, it differed per sensitivity analysis in comparison to which other bridge mental states the mean bridge effect of “feeling down” was significantly stronger (see Additional file 1: Tables S6-S8).

**Table 3:** Posterior means and 95% credible intervals (CIs) for the mean bridge effect (i.e., bridge effect divided by the number of indirect effects) of each mental state in each outcome group

Mental state	Comorbidity group, estimate [CI]	Depression-only group, estimate [CI]	Anxiety-only group, estimate [CI]
Not relaxed	0.0007* [− 0.0001, 0.0014]	0.0005 [− 0.0005, 0.0017]	0.0003* [− 0.0012, 0.0018]
Nervous	0.0011* [0.0005, 0.0018]	0.0001* [− 0.0008, 0.0010]	0.0007* [− 0.0004, 0.0019]
Irritated	0.0002* [− 0.0005, 0.0008]	0.0000* [− 0.0005, 0.0006]	− 0.0004* [− 0.0018, 0.0008]
Worrying	0.0014* [0.0008, 0.0021]	0.0009 [0.0001, 0.0022]	0.0003* [−0.0007, 0.0014]
Not cheerful	0.0018 [0.0010, 0.0027]	**0.0016 [0.0004, 0.0033]**	0.0016 [0.0002, 0.0034]
Listless	0.0003* [− 0.0003, 0.0009]	0.0000 [− 0.0011, 0.0012]	0.0003* [− 0.0006, 0.0014]
Down	**0.0028 [0.0018, 0.0039]**	0.0005 [− 0.0003, 0.0017]	**0.0043 [0.0021, 0.0069]**

Estimates accompanied by an asterisk (*) were significantly different from the bridge effect with the highest point estimate (bolded) within that group

## Discussion

In our study, we tested the hypothesis that bridge mental states play a role in comorbidity among psychiatric disorders. In a sample of individuals with comorbid depression and anxiety, we did not find clear support for a bridge function of mental states in their mental state network. First, we did not find that the two a priori hypothesized overlapping mental states for anxiety and depressive disorders (“worrying” and “feeling irritated”) had a stronger bridging function in the comorbid group compared to the single disorder groups. Second, we observed that “feeling down,” a non-overlapping mental state, featured a bridge effect of comparable strength as “worrying” in both the comorbid group and the anxiety-only group. Thus, our findings are not in line with the bridge symptom hypothesis [10].

Network theory literature has provided numerous hypothetical examples of which symptoms (i.e., overlapping or non-overlapping) may play which role (e.g., forming the link between disorder symptom clusters) in the development of comorbid disorders. These discussions about the bridge hypothesis have concentrated on the network’s structure, while limited attention has been paid to the network’s state, that is, the activation of symptoms and specifically the timing of this activation in the context of comorbidity development [60, 61]. Questions such as “when is the activation of bridge symptoms, or bridge mental states, relevant and detectable?” have therefore remained largely unaddressed. An exception is provided by Borsboom [34], who described four phases, in which interactions between network activation and structure are linked to the development of psychopathology. These can also be applied to the development of comorbidity. Borsboom distinguishes (i) an asymptomatic phase, with a dormant structure and no symptom activation, and thus no active psychopathology development; (ii) a network activation phase in which an external event triggers some of the symptoms; (iii) a symptom spread phase in which connected symptoms (incl. bridge symptoms) become activated and psychopathology starts to emerge; and (iv) a symptom maintenance phase, in which activation is perpetuated due to a strongly connected network containing feedback loops and a full-blown episode of a mental disorder is experienced. Although Borsboom’s account of psychopathology development is quite comprehensive, it reflects a progressive process that stops at symptom maintenance. Our view is that symptom remission also forms a relevant part of the course of psychopathology, and may encompass different phases than those previously mentioned. Such a view is in line with the clinical staging model [62] and with the scar hypothesis [63]. In particular, it is conceivable that a network underlying risk for a first episode of a psychiatric disorder is different from a network underlying risk for a second or third episode [53]. We therefore suggest to add two additional phases, a (v) partial remission phase, in which individuals due to residual symptoms have a heightened risk to return to phase iii, or alternatively progress to a (vi) full remission phase, in which risk for recurrence is still higher as compared to individuals in phase i that have never experienced a psychiatric disorder. Determining in which of these six phases bridge symptoms are relevant and detectable is necessary to establish whether they potentially can be used to prevent or treat comorbidity. Although the question concerning the phase in which to detect bridge effects was not our a priori purpose, our findings aid in further specification thereof, as discussed hereafter.

One of our starting points was that network structure, including the presence of bridge mental states, reflects underlying vulnerability to developing psychopathology [64–66]. This would mean that we would be able to observe bridge effects in individuals vulnerable to experiencing comorbid depression and anxiety. Our sample therefore included both individuals with (re) current psychopathology and individuals at conceivable risk of recurrence of comorbid depression anxiety due to previous experience of these disorders. Given this starting point, and due to its various strengths, the NESDA-EMAA design provided an excellent opportunity to test for the expected group differences in bridge effects between individuals with comorbid and single disorders. First, with nearly 400 participants who had been diagnosed with depression, anxiety, or both, it is one of the largest EMA studies to date. Second, the diagnostic history was assessed in multiple waves up to 12 years prior to EMA assessment for each participant, allowing us to rule out possible comorbidity prior to the EMA study and to allocate participants to each diagnostic group with a high degree of certainty. Third, the number of EMA assessments (max. 70 per person) in NESDA-EMAA is on the higher range of what has been used in dynamic network papers [27, 66, 67], and participants had very few missing observations. However, despite the strengths of this dataset, we did not find support for any group differences in bridge effects.

Yet, absence of evidence for the bridge hypothesis does not imply evidence of absence. This requires us to evaluate what aspects of our design may have impeded finding a role for bridge effects specific to comorbid disorders. We identified three aspects. First, the current design did not allow for investigation of the bridge hypothesis during all phases of psychopathology development. For instance, our sample did not include individuals who were in the active phase of developing a first onset of comorbid depression and anxiety during the EMA assessment, and would thus be in phase iii. Participants in the current sample with comorbid depression and anxiety had already experienced both disorders at the time of the EMA assessment, and the vast majority had their first onset at least 9 years back. Although some participants may have been in the process of developing comorbidity recurrence (phase iii) during the EMA assessments, the vast majority of the comorbid group were likely to be in disease phases iv, v, and vi. If bridge mental state activity is particularly relevant during the period that comorbidity first develops or re-develops (both phase iii), our design, in which we studied a sample that showed heterogeneity in the phase of illness participants were in, was not optimal to detect bridge mental states. This may be one of the reasons for observing a null result. In the subset of participants who were roughly in an active episode (52.4%) (i.e., within 6 months of EMA) (phases iv and v), we observed a stronger bridge effect for “worrying” as compared to the total comorbid group, which also included a large subset of individuals in phase vi (42.0%). Although the difference in bridge effects between the subgroup in this approximate acute comorbid state and the depression-only group was still not significant (difference with the anxiety-only group was significant), it provides some initial indication that bridge effects are detectable and potentially of relevance in the activated symptom phase and that bridge effects are difficult to detect or potentially irrelevant in individuals who are vulnerable for comorbidity but in remission. If our initial findings are replicated, such that vulnerable network structures are completely “dormant” until first symptoms are activated [34], or reactivated (both phase iii), it may be ineffective to target the bridge symptoms in preventative efforts, unless applied during active phases of comorbidity (re-)occurrence. The latter, however, is difficult to predict. Treatment may still be feasible and effective, starting EMA as soon as patients enter care and have active symptoms. Clearly, these ideas need further research.

The second design-related aspect pertains our group-level analyses. Perhaps bridge mental state effects are only discernible at the level of the individual, and not of diagnostic groups. Associations between mental states may vary too much from person to person for average group-level indirect effects to be meaningful. Post hoc inspection of variation in the random effects showed considerable differences between individuals, with lagged association ranging from negative to positive effects, which in turn also affected the sizes of the indirect effects. Individual variation in the size of effects may be due to heterogeneity in symptom presentation and therefore also the experience (and personal relevance) of certain mental states. This is an issue within one disorder category [68], and likely even more so in a group of individuals with comorbid psychopathology. Therefore, if (i) any mental state (overlapping and non-overlapping) may function as a bridge, and (ii) this bridge is different for each individual, falsifying the bridge symptom hypothesis at the group-level becomes difficult. Person-specific models might be one way forward. Fisher et al. [69], for instance, modeled networks consisting of 21 depression and anxiety symptoms for 40 individuals. In their discussion of three exemplar participants, it was clear that individuals not only starkly differed from each other, but also that the most important symptoms for each individual were not necessarily the principal diagnostic criteria of their diagnoses. Although person-specific models can provide new insight in person-specific mechanisms, such models may also require sampling of a large range of variables for each individual in order to be able to detect which mental states are most relevant for a specific individual. Furthermore, testing such complex models that require many parameters to be estimated will also require much longer time series in order to obtain reliable estimates at the individual-level. Although also not without problems, future research may consider analytical techniques that provide compromises between idiographic (person-specific) and nomothetic (group-level) analytical approaches [70, 71].

The third design-related aspect pertains to how we model symptom dynamics: using a linear multilevel first-order VAR model fit to data collected at approximately 3-h intervals. Perhaps, bridge-activation dynamics operate over a shorter or over a much longer timescale than the interval sampled in our design (for a discussion see [72]). In the absence of strong theory to inform us about the optimal lag length at which mental states influence each other, we estimated a lag 1 VAR model to keep our model as parsimonious as possible. It is however, conceivable that some of the modeled effects also occurred over multiple lags, and not accounting for these is thus a limitation of our study. Data-driven approaches to recover the optimal lag of time-series models are developed (e.g., DVTEM developed by [73]), but do not yet exist for models including random effects or that include more than two variables (i.e., such as the model used in this study). Further development of theory to inform the choice of lag length and data-driven tools to model multiple lag lengths simultaneously is necessary to aid appropriate modeling of within-person dynamics. Moreover, although VAR models are frequently used to model psychological dynamics (e.g., [59, 66, 67]), they are somewhat limited in the types of dynamics they can describe. For example, while they allow for relating all variables to one another at the next measurement occasion, they cannot describe systems which switch from one stable state (e.g., asymptomatic) to another (e.g., symptomatic) over time [55, 72, 74]. The latter may be something we expect to observe in individuals moving between phases of pathology (e.g., phase iii to phase iv). This limitation may be somewhat mitigated by the use of more complex statistical models, such as time-varying VAR models, which allow in principle to whether parameter values change over the study period (see [75], for an overview). However, these models do not yet have a multilevel extension, and require much longer time series to perform acceptably than we have available for individual participants in the current study (cf. [76]). The development of more formalized accounts of symptom dynamics is critical in guiding future efforts at capturing dynamic bridge symptom relationships [77]. Absent of such developments, the VAR model is a reasonable first step in approximating those dynamics with a linear model.

A pertinent question arising from this study is which mental states should be considered as bridge mental states in future research? Although the bridge effect of “worrying” was not significantly stronger in the comorbidity compared to the single disorder groups, our findings nonetheless indicate that it did funnel activity from the depression to anxiety mental state cluster and vice versa. This seems in line with findings from epidemiological and clinical research, in which “worrying” or related symptoms such as “ruminating” or “repetitive negative thinking” have been found to function as a mediator between depression and anxiety [78–81], and may suggest that the mental state worrying reflects a momentary expression of the symptom worrying. In contrast, the other overlapping mental state “feeling irritated” did not feature a bridge effect in any diagnostic group. This effect could not be explained by low within-person variation in this mental state, as feeling irritated was one of the most highly varying mental states in every group. One possibility is that “feeling irritated” is not a good momentary proxy of irritability as symptom. Future research is needed to address the link between symptoms and within-day affect, as currently no study has examined the association between changes in daily symptoms and within-day changes in affect. A second possibility is that our findings indicate that “feeling irritated” is not relevant as bridge mental state, at least in adulthood. Irritability features in diagnostic criteria of several anxiety disorders (i.e., GAD, PTSD). While often associated with major depression [82] and considered an indicator of depression in children and adolescents in the Diagnostic and Statistical Manual of Mental Disorders, DSM-5 [83], irritated mood is not officially part of the diagnostic criteria for adults. Therefore, irritated mood might still be an appropriate bridge mental state or symptom in children and adolescents. This needs to be further investigated. Likewise, other bridge mental states or symptoms need to be investigated as well. Currently, only two bridge mental states were assessed, while there are many other symptoms (e.g., insomnia [84]), behaviors (e.g., avoidance behaviors [85]), or cognitive processes (e.g., attributional styles [86]) that have previously been identified as shared features of depressive and anxiety disorders. Ideally, complex constructs like avoidance behavior or repetitive negative thinking would be measured using multiple items. Whether different types of mediators influence development of comorbidity in different people would be particularly interesting follow-up question.

Our finding that “feeling down” featured a large bridge effect in the comorbidity and the anxiety-only groups also requires further study. This finding was somewhat unexpected for two reasons. First, prolonged “sad mood” is part of the criteria of depression and does not officially feature in diagnostic criteria for other psychiatric disorders than depression-related syndromes. Yet “feeling down” included in the EMA might be a less severe, overlapping, mental state in the sense that it accompanies feelings of distress that are present in other conditions. Second, we had not expected “feeling down” to play a role in the dynamics of the anxiety-only group. The majority of this group had never experienced a comorbid depression diagnosis and the others not in at least 9 years. This finding shows two things: (1) apparently, it is more difficult to find “pure” anxiety, than “pure” depression, which might be a consideration to keep in mind for studies wanting to improve the current design, and (2) it shows that subthreshold symptoms (in this case of depression) that may not be sufficient for obtaining a comorbid diagnosis may still play a role in the daily mental state dynamics, which would explain the presence of bridge effects in this group. Future research is necessary to investigate whether the bridge hypothesis may be more about a matter of degree of risk, also including risk for subthreshold complaints of a second disorder.

Because the bridge symptom hypothesis had not previously been investigated using dynamical data, we developed a new approach to estimate bridge mental state effects. We believe that the current approach for estimating the bridge mental state effect, that is, summing all indirect effects from the depression to the anxiety-cluster via the bridge mental states and vice versa, has face validity. It has conceptual similarity to bridge centrality, which estimates nodes’ bridging function in relation to multiple symptom clusters [87]. Bridge centrality has however not been extended to directed networks based on time-series data, for which it is possible to take into account that a bridge effect would unfold over multiple time points (i.e., lags). Future research may come up with alternative methods to estimate the bridge mental state effects in dynamic data.

A limitation of the current study is that individuals could start EMA assessments within 31 days of the clinical interview; it was therefore difficult to establish whether patients at the time of the EMA assessments were in an acute episode. Also, statistical power is potentially an issue. No standard exists for conducting power analyses to determine an appropriate sample size and number of observations in multilevel VAR models [88]. For multilevel models more generally, power is determined both by the number of participants and repeated measures [89]. A recent simulation study of a multilevel autoregressive (AR) model showed that particularly the number of participants (and their similarity) was important for the estimation precision of the parameters [90], but these results are difficult to generalize to a 7-variate VAR model, since it has previously been shown that sample size requirements for the recovery of VAR parameter depend heavily on the true parameter values (particularly the size of the true cross-lagged parameters [91]). In the current study however, due to relatively few missing assessments, the average number of time points per person (i.e., 60 time points) does exceed the length of the time series used in many previous analyses of this type (e.g., [59, 92–94]). Although we have a large sample size for the comorbid group (*n* = 143), a limitation of the current study is the smaller sample sizes in the single disorder groups. This means that average parameter estimates in those groups were estimated with more uncertainty, and thus should be viewed in that light.

Finally, it is important to consider that although the network approach theorizes causal relations between symptom variables, the use of observational data in general precludes us from applying a direct causal interpretation to the statistical relationships estimated in the current paper. For instance, the statistical relationships estimated here are likely influenced by the presence of unmeasured confounding variables. Although multilevel lagged regression models with person mean-centering have been discussed as a method that can control for the effect of unmeasured time-invariant confounding variables (cf. [95]), there are likely numerous unobserved time-varying confounders present in the current setting (such as additional unmeasured symptoms, mental states, and contextual factors). Additionally, misspecification of the causal timescale, as previously discussed, and the functional form of the temporal relationships would also undermine veracity of causal conclusions made on the basis of estimated cross-lagged effects [72]. To improve the study of causal symptom relationships, we likely need to develop a combination of novel experimental manipulations and more precise formal theoretical models of symptom dynamics which incorporate contextual factors [77].

## Conclusion

This study was the first to investigate whether mental states of “worrying” and “irritability” that are part of both depression and anxiety serve as bridge mental states in the daily mental state dynamics in individuals with comorbid anxiety and depression. Although particularly “worrying” may still be a candidate in future research, we did not find strong support for a bridging role in the dynamic mental state network that is specific to risk for comorbid depression and anxiety. Potentially, bridge mental state activity is most relevant (and detectable) during the period that comorbidity (re-)develops, or bridge mental states are only detectable in person-specific networks rather than group-level models. If bridge mental states in person-specific networks are found to play a role in the development of comorbid depression and anxiety, these would be highly interesting targets for treatment in clinical practice. Therefore, more research is needed, especially prospective studies during active development of comorbidity and/or at the individual-level.

## Supplementary information

**Additional file 1.**

**Additional file 2.**

## Data Availability

According to European law (GDPR), data containing potentially identifying or sensitive patient information are restricted; our data involving clinical participants are not freely available in a public repository. However, data are available upon request via the NESDA Data Access Committee (nesda@ggzingeest.nl).

## Footnotes

1 Four participants, who based on their diagnostic history could be allocated to the comorbidity group (*n* = 1), depression-only group (*n* = 2), or anxiety-only group (*n* = 1), were excluded due to completing < 30% of EMA assessments. We do not think that excluding these four individuals has influenced our results.

2 Please note that this is a deviation from our preregistration. We had planned to model eight mental states instead of seven. During the modeling process, it appeared that although the model including the momentary mental state “I feel anxious” (ANX; “Ik voel me angstig”) converged, the Mplus tracing plots showed convergence problems for parameters involving this variable. This means that estimates for these parameters would likely be unreliable. Moreover, proposed sensitivity analyses would not converge for the eight-variable model. We therefore excluded “I feel anxious” from further analyses. “Feeling anxious” had very low variance in all groups. For transparency, we present the results of our main analyses which do include “feeling anxious” in Additional File 1 in Supplementary Figures S1-S2

3 Please note that this is a deviation from the preregistration. The preregistration was informed by a previous intensive longitudinal study during which data was collected during an intervention. For that study, non-stationarity was therefore expected. Data in the current study was not collected during an intervention. Additionally, as a sensitivity analysis, we have run our models based on linearly detrended data, which yielded the exact same results. Plots based on these models are presented in Additional File 1 Fig. S3-S4.

4 Using the shortest time-interval (i.e., 120 min) available in the dataset did not lead us to draw different conclusions.

5 Please note that this is a different sensitivity analysis than that was proposed in our preregistration. Our original plan was to only include individuals who did not have a current diagnosis (no diagnosis in the 6 months prior to the EMA study; *n* = 68 in the comorbid group, *n* = 33 in the depression-only group, and *n* = 25 in the anxiety-only group). This was to evaluate whether effects remained if individuals with most recent and therefore more severe psychopathology were excluded. However, because no group differences were observed, it was not of interest to investigate whether effects remained.
