# Supplementary material for: Comorbidity between depression and anxiety: assessing the role of bridge mental states in dynamic psychological networks
Source: BMC Med. 2020 Sep 29;18:308. doi: 10.1186/s12916-020-01738-z (PMC7523307; doi:10.1186/s12916-020-01738-z)
Supplement: Supplementary file 1 — Additional file 1. [file 12916_2020_1738_MOESM1_ESM.docx]

ADDITIONAL FILE 1

**Comorbidity between depression and anxiety: assessing the role of bridge mental states in dynamic psychological networks.**

Robin. N. Groen , Oisín Ryan, Johanna T.W. Wigman, Harriette Riese, Brenda W.J.H. Penninx, Erik J. Giltay, Marieke Wichers, Catharina A. Hartman

Content:

1. Preregistration, deviations and sensitivity analyses pg. 2
2. Additional group allocation information pg. 3
3. Data preprocessing pg. 3
4. Mplus specifications Dynamic Structural Equation Model pg. 3
5. Table S1-S2 pg. 5
6. Table S3 pg. 6
7. Table S4 pg. 7
8. Table S5 pg. 8
9. Table S6 pg.10
10. Table S7-S8 pg. 10
11. Figure S1 pg. 12
12. Figure S2 pg. 13
13. Figure S3 pg. 14
14. Figure S4 pg. 15
15. Figure S5 pg. 16
16. Figure S6 pg. 17
17. Figure S7 pg. 18
18. Figure S8 pg. 19
19. Figure S9 pg. 20
20. Figure S10 pg. 21

**1. Preregistration, deviations and sensitivity analyses**

Aims, analyses, variable selection and operationalization were registered prior to accessing the data. The preregistration form and code can be downloaded via https://osf.io/jwuz9. Data are available upon request via the NESDA Data Access Committee (nesda@ggzingeest.nl).

Analyses deviated from the planned analyses in the preregistration in a few respects. The first change is that we had planned to include eight variables in our analyses instead of seven. During the modeling process, we noticed convergence problems when including the variable “I feel anxious”. These convergence issues might be related to this variable having low variance in all groups. Results of the eight variable model are included in this supplement on page 13. A second deviation from our preregistration is that we within-person standardized our variables prior to the analyses in Mplus (see section 4 below for more details on this change). Additionally, we did not detrend the data although this was registered. This was motivated by our observation that for the majority of individuals (between 71%-91% depending on the variable) time series were stationary. Furthermore, we had preregistered that we would test group differences in bridge effects by means of permutation tests in R. However, after running the DSEM model we realized that we could utilize the Bayesian credible intervals for this purpose, which made permutation tests superfluous. Finally, we had registered three sensitivity analyses to assess to what extent findings were influenced by (i) severity differences, (ii) recency of diagnoses, and (iii) sex differences. We conducted the first sensitivity analysis as planned. However, we changed the second sensitivity analysis. Our initial plan was to investigate whether any group differences remained if we only investigated individuals without a current (<6 month) diagnosis. This was motivated by the idea that network structure may signal underlying vulnerability for psychopathology even if problems are currently absent (Borsboom & Cramer, 2013; Borsboom, 2017). Because we did not observe group differences in our main analysis, this sensitivity analysis became irrelevant. Instead, we chose to compare individuals of the comorbid group with a current diagnosis to the other two groups to investigate whether differences would become more pronounced. This would then make a case that this hypothesis may perhaps only be studied in individuals with current complaints. The third sensitivity analysis about sex differences was not conducted. This was decided after noticing rather wide credible intervals for our main analysis, which made us realize that we would not have power to compare even smaller subgroups.

**2. Additional group allocation information**

Some individuals in the single diagnosis groups had previously (before the baseline NESDA measurement) met criteria for a diagnosis of the other disorder. To include as many individuals in the single diagnosis groups, and subsequently maximize power, we investigated whether individuals with or without prior-to-NESDA diagnosis differed in current symptom severity scores (IDS, BAI, FQ) of that other diagnosis. In the anxiety group there was no difference on IDS scores in individuals with or without depressive disorder prior to NESDA (see Supplemental Table 1). Individuals in the anxiety-only group were therefore allowed to have met criteria for MDD before partaking in NESDA. These individuals had not experienced a depressive disorder for at least 9 years. However, individuals in the depression-only group with an anxiety disorder prior to NESDA experienced more current anxiety symptoms as compared to individuals without a prior anxiety diagnosis (see Supplemental Table 2). Therefore, we selected only individuals with a depression diagnosis during NESDA who did not have an anxiety diagnosis prior to NESDA for the depression-only group.

**3. Data preprocessing**

First, we checked whether individuals had completed sufficient assessments. Individuals with less than 30% completed assessments were removed prior to the analysis. Second, we within-person standardized the variables prior to analysis in Mplus. This enables to compare the relative strengths of different predictors within and between individuals, and has been recommended because differences in coefficients may be due to differences in variance (Bulteeel et al., 2016; Schuurman et al., 2016). In principle, we would prefer not to standardize before the analysis and this is a deviation from the preregistration: DSEM in Mplus automatically centers individuals around their mean, and allows us to extract within-person standardized parameters. However, it is not possible to directly regress within person standardized parameters on between-person dummy variables, or to automatically calculate indirect effects made up of within-person standardized parameters. After consultation with the developers of Mplus, we settled on the approach described in the main text.

**4. Mplus specifications Dynamic Structural Equation Model and treatment of missing data**

We used the default specifications of DSEM, which performs Bayesian estimation with non-informative priors based on two independent Monte Carlo Markov Chain (MCMC) chains. The priors for the variance-covariance matrix of the residuals and the variance-covariance matrix of the random intercepts are improper Inverse Wishart priors, with matrix parameters all equal to zero, and df equal to -8. As only the random slope variances (and not their covariances) are estimated, the priors for the random slopes terms are improper uninformative inverse gamma distributions, IG(-1,0). For all other parameters (fixed effects and the regression of fixed effects on group membership) the priors are normal distributions with zero mean and infinite variance (Asparouhov, Hamaker & Muthen 2018). We observed a consistent potential scale reduction (PSR) value below 1.1 (indicating model convergence) after 20000 iterations. We doubled the number of iterations to confirm that PSR remained stable <1.1. For our final model we specified 40 000 iterations for each Markov Chain Monte Carlo (MCMC) chain, and used the option THIN = ‘4’, to specify that every fourth iteration from the posterior distribution would be used in the parameter estimation. In Bayesian analyses, missing values are treated as any other unknown parameter that needs to be estimated, meaning that they are sampled from their conditional posterior during each iteration of the MCMC algorithm (see Hamaker et al., 2018 for a detailed explanation). Here, it is assumed that observations are missing at random (MAR), we checked this assumption at the within-person level by evaluating whether missingness of observations was associated with day of the week, time of the day, time since the beginning of the study, and whether missingness of observations could be explained by the values of the observations prior to the missing value. We observed an effect of time since the beginning of the study on the probability that observations were missing, but no other effects. Because we observed that no time trend influenced the observations, and because time-trends are not explicitly part of our model, we do not think this pattern of missingness is problematic. At the between-person level, a weak effect of sex appeared to be associated with number of missing values. This effect appeared to be driven by two individuals of the same sex who had a very high degree of missing values. We do not think this will have affected our conclusions.

| **Table S1.** Means, and standard deviations on the Inventory of Depressive Symptom scale between individuals in the anxiety-only group with and without depressive disorder prior to NESDA | | |
| --- | --- | --- |
|  | Anxiety only no lifetime depression (n=22) | Anxiety with lifetime depression (N=16) |
| Inventory of Depressive Symptoms | 11.4 (5.6) | 11.3 (6.4) |

| **Table S2.** Means, and standard deviations on the Fear Questionnaire, and Beck Anxiety Inventory between individuals in the depression-only group with and without anxiety disorder prior to NESDA | | |
| --- | --- | --- |
|  | Depression only no lifetime anxiety (n=42) | Depression with lifetime anxiety (N=15) |
| Fear Questionnaire | 8.6 (11.6) | 14.3 (12.2) |
| Beck Anxiety Inventory | 5.3 (5.7) | 6.3 (7.1) |

| **Table S3.** Multicollinearity diagnostic | |
| --- | --- |
|  | Variance Inflation Factor (VIF) |
| Not relaxed_t-1_ | 1.42 |
| Nervous_t-1_ | 1.35 |
| Irritated_t-1_ | 1.34 |
| Worrying_t-1_ | 1.35 |
| Not cheerful_t-1_ | 1.50 |
| Listless_t-1_ | 1.24 |
| Down_t-1_ | 1.49 |
| a. Dependent variable: Not relaxed_t_; VIFs are the same for when the other predictors function as dependent variable. | |

| **Table S4.** Standardized estimates and 95% credible intervals (CIs) of lag(1) associations between mental states for the comorbid, depression-only and anxiety-only group. | | | |
| --- | --- | --- | --- |
|  | Comorbid group | Depression only | Anxiety only |
|  | Estimate (CI) | Estimate (CI) | Estimate (CI) |
| Nervous _t_ |  |  |  |
| Not relaxed _t-1_ | 0.112* (0.081, 0.143) | 0.113* (0.057, 0.171) | 0.094* (0.032, 0.154) |
| Nervous_t-1_ | 0.073* (0.042, 0.104) | 0.028 (-0.033, 0.088) | 0.089* (0.03, 0.148) |
| Irritated_t-1_ | 0.012 (-0.019, 0.042) | -0.002 (-0.057, 0.054) | -0.071* (-0.127, -0.015) |
| Worrying_t-1_ | 0.033* (0.004, 0.062) | 0.043 (-0.009, 0.096) | 0.001 (-0.052, 0.055) |
| Not cheerful_t-1_ | 0.069* (0.038, 0.1) | 0.075* (0.022, 0.13) | 0.126* (0.067, 0.186) |
| Listless_t-1_ | 0 (-0.028, 0.029) | -0.025 (-0.077, 0.028) | 0.017 (-0.04, 0.074) |
| Down_t-1_ | 0.063* (0.031, 0.094) | 0.054 (-0.001, 0.111) | 0.105* (0.045, 0.165) |
| Nervous _t_ |  |  |  |
| Nervous _t_ |  |  |  |
| Not relaxed _t-1_ | 0.024 (-0.007, 0.055) | 0.006 (-0.053, 0.067) | 0.014 (-0.045, 0.072) |
| Nervous_t-1_ | 0.194* (0.158, 0.23) | 0.076* (0.002, 0.147) | 0.207* (0.137, 0.277) |
| Irritated_t-1_ | -0.005 (-0.036, 0.026) | 0.017 (-0.043, 0.076) | -0.009 (-0.065, 0.046) |
| Worrying_t-1_ | 0.089* (0.057, 0.122) | 0.061* (0.002, 0.12) | 0.094* (0.036, 0.151) |
| Not cheerful_t-1_ | 0.01 (-0.022, 0.043) | -0.014 (-0.075, 0.047) | -0.017 (-0.078, 0.044) |
| Listless_t-1_ | 0.013 (-0.017, 0.043) | 0.039 (-0.019, 0.098) | -0.016 (-0.073, 0.039) |
| Down_t-1_ | 0.041* (0.002, 0.079) | -0.001 (-0.071, 0.072) | 0.099* (0.029, 0.169) |
|  |  |  |  |
| Irritated _t_ |  |  |  |
| Not relaxed _t-1_ | 0.057* (0.027, 0.087) | -0.019 (-0.075, 0.035) | 0.029 (-0.032, 0.087) |
| Nervous_t-1_ | 0.058* (0.027, 0.09) | 0.026 (-0.036, 0.088) | 0.013 (-0.046, 0.072) |
| Irritated_t-1_ | 0.108* (0.075, 0.141) | 0.046 (-0.013, 0.106) | 0.057 (-0.002, 0.116) |
| Worrying_t-1_ | 0.026 (-0.004, 0.056) | 0.037 (-0.017, 0.091) | 0.024 (-0.032, 0.078) |
| Not cheerful_t-1_ | 0.014 (-0.019, 0.047) | 0.052 (-0.006, 0.11) | 0.027 (-0.035, 0.089) |
| Listless_t-1_ | 0.005 (-0.026, 0.036) | -0.024 (-0.083, 0.033) | 0.015 (-0.044, 0.076) |
| Down_t-1_ | 0.033 (-0.002, 0.069) | -0.017 (-0.081, 0.047) | 0.11* (0.043, 0.175) |
|  |  |  |  |
| Worrying _t_ |  |  |  |
| Not relaxed _t-1_ | -0.022 (-0.051, 0.007) | 0.048 (-0.004, 0.101) | -0.018 (-0.077, 0.042) |
| Nervous_t-1_ | 0.046* (0.013, 0.078) | 0.009 (-0.055, 0.073) | 0.021 (-0.045, 0.087) |
| Irritated_t-1_ | 0.007 (-0.021, 0.035) | -0.009 (-0.06, 0.043) | 0.004 (-0.051, 0.057) |
| Worrying_t-1_ | 0.263* (0.224, 0.301) | 0.214* (0.141, 0.285) | 0.356* (0.282, 0.429) |
| Not cheerful_t-1_ | 0.061* (0.029, 0.093) | 0.027 (-0.029, 0.083) | 0.026 (-0.038, 0.091) |
| Listless_t-1_ | 0.019 (-0.008, 0.047) | -0.006 (-0.058, 0.047) | 0 (-0.055, 0.056) |
| Down_t-1_ | 0.085* (0.05, 0.12) | 0.095* (0.032, 0.158) | 0.026 (-0.045, 0.097) |
|  |  |  |  |
| Not Cheerful _t_ |  |  |  |
| Not relaxed _t-1_ | 0.025 (-0.005, 0.055) | 0.084* (0.026, 0.14) | 0.045 (-0.014, 0.104) |
| Nervous_t-1_ | -0.002 (-0.031, 0.027) | -0.011 (-0.071, 0.049) | -0.012 (-0.07, 0.045) |
| Irritated_t-1_ | 0.021 (-0.008, 0.049) | -0.023 (-0.077, 0.03) | -0.028 (-0.079, 0.026) |
| Worrying_t-1_ | 0.041* (0.01, 0.072) | 0.064* (0.005, 0.123) | 0.064* (0.007, 0.122) |
| Not cheerful_t-1_ | 0.16* (0.127, 0.195) | 0.140* (0.079, 0.201) | 0.203* (0.137, 0.269) |
| Listless_t-1_ | 0.049* (0.02, 0.079) | 0.086* (0.029, 0.142) | 0.081* (0.024, 0.14) |
| Down_t-1_ | 0.104* (0.071, 0.138) | 0.011 (-0.051, 0.073) | 0.087* (0.023, 0.151) |
|  |  |  |  |
| Listless _t_ |  |  |  |
| Not relaxed _t-1_ | 0.008 (-0.023, 0.04) | -0.014 (-0.074, 0.046) | 0.014 (-0.047, 0.074) |
| Nervous_t-1_ | -0.003 (-0.033, 0.027) | -0.039 (-0.101, 0.022) | -0.025 (-0.082, 0.032) |
| Irritated_t-1_ | -0.024 (-0.056, 0.007) | 0.059 (-0.002, 0.119) | 0.039 (-0.018, 0.098) |
| Worrying_t-1_ | 0.03 (-0.002, 0.061) | 0.011 (-0.048, 0.071) | 0.018 (-0.039, 0.075) |
| Not cheerful_t-1_ | 0.076 (0.043, 0.109) | 0.118* (0.057, 0.18) | 0.08* (0.017, 0.144) |
| Listless_t-1_ | 0.164 (0.127, 0.201) | 0.078* (0.006, 0.15) | 0.171* (0.1, 0.242) |
| Down_t-1_ | 0.094 (0.058, 0.13) | 0.024 (-0.046, 0.094) | -0.011 (-0.081, 0.058) |
|  |  |  |  |
| Down _t_ |  |  |  |
| Not relaxed _t-1_ | -0.002 (-0.031, 0.026) | 0.057* (0.002, 0.111) | 0.021 (-0.037, 0.078) |
| Nervous_t-1_ | 0.017 (-0.014, 0.047) | 0.02 (-0.046, 0.085) | 0.034 (-0.027, 0.095) |
| Irritated_t-1_ | 0.018 (-0.01, 0.045) | -0.017 (-0.073, 0.038) | 0.046 (-0.007, 0.099) |
| Worrying_t-1_ | 0.092 (0.058, 0.125) | 0.022 (-0.041, 0.087) | 0.099* (0.036, 0.161) |
| Not cheerful_t-1_ | 0.095 (0.066, 0.126) | 0.057* (0.002, 0.114) | 0.061* (0.001, 0.121) |
| Listless_t-1_ | 0.044 (0.015, 0.073) | 0.003 (-0.057, 0.063) | 0.086* (0.029, 0.143) |
| Down_t-1_ | 0.182 (0.143, 0.221) | 0.198* (0.123, 0.271) | 0.072 (-0.005, 0.148) |

Note: estimates accompanied by an asterisk (*) were significantly different from zero for that outcome group.

| **Table S5.** Standard deviations, and range of lag(1) associations between mental states across groups. | | | | | | | | |
| --- | --- | --- | --- | --- | --- | --- | --- | --- |
|  | Not relaxed_t_  SD (range) | Not cheerful_t_  SD (range) | Irritated_t_  SD (range) | Listless_t_  SD (range) | Down_t_  SD (range) | Nervous_t_  SD (range) | Worrying_t_  SD (range) |  |
| Not relaxed_t-1_ | 0.030  (0.03-0.22) | 0.029  (-0.08-0.14) | 0.031  (-0.04-0.09) | 0.03  (-0.07-0.08) | 0.029  (-0.05-0.1) | 0.024  (-0.05-0.09) | 0.029  (-0.07-0.08) |  |
| Not cheerful_t-1_ | 0.030  (0.01-0.18) | 0.063  (-0.04-0.34) | 0.038  (-0.07-0.33) | 0.04  (-0.03-0.2) | 0.034  (-0.02-0.18) | 0.036  (-0.09-0.09) | 0.043  (-0.1-0.2) |  |
| Irritated_t-1_ | 0.045  (-0.13-0.14) | 0.025  (-0.06-0.05) | 0.055  (-0.08-0.21) | 0.052  (-0.09-0.21) | 0.022  (-0.05-0.06) | 0.031  (-0.09-0.09) | 0.019  (-0.05-0.06) |  |
| Listless_t-1_ | 0.031  (-0.06-0.09) | 0.036  (-0.04-0.18) | 0.045  (-0.12-0.14) | 0.105  (-0.09-0.4) | 0.042  (-0.12-0.19) | 0.034  (-0.09-0.18) | 0.023  (-0.09-0.08) |  |
| Down_t-1_ | 0.029  (-0.01-0.16) | 0.054  (-0.09-0.21) | 0.063  (-0.13-0.26) | 0.075  (-0.13-0.31) | 0.124  (-0.33-0.52) | 0.087  (-0.16-0.36) | 0.06  (-0.18-0.22) |  |
| Nervous_t-1_ | 0.053  (-0.14-0.23) | 0.039  (-0.14-0.13) | 0.051  (-0.08-0.17) | 0.035  (-0.12-0.11) | 0.06  (-0.1-0.36) | 0.112  (-0.1-0.61) | 0.071  (-0.15-0.29) |  |
| Worrying_t-1_ | 0.028  (-0.05-0.11) | 0.048  (-0.08-0.16) | 0.026  (-0.04-0.13) | 0.044  (-0.09-0.16) | 0.083  (-0.17-0.44) | 0.052  (-0.03-0.34) | 0.133  (-0.1-0.66) |  |

| **Table S6.** Posterior means and 95% Credible Intervals (CI) for the mean bridge effect (i.e., bridge effect divided by the number of indirect effects) of each mental state in the lowest severity tertile of the comorbid group, and the original depression-only and anxiety-only groups | | | |
| --- | --- | --- | --- |
|  | Comorbidity group (n=46) | Depression-only group (n=40) | Anxiety-only group (n=37) |
| Mental state | Estimate [CI] | Estimate [CI] | Estimate [CI] |
| Not Relaxed | 0.0009* [-0.0004, 0.0022] | 0.0005 [-0.0005, 0.0018] | 0.0004* [-0.001, 0.0019] |
| Nervous | 0.0004 [-0.0005, 0.0014] | 0.0002 [-0.0008, 0.0012] | 0.0007* [-0.0003, 0.0021] |
| Irritated | 0.0002* [-0.0007, 0.0012] | 0.0001* [-0.0006, 0.0007] | -0.0004* [-0.0019, 0.0008] |
| Worrying | 0.001* [-0.0001, 0.0023] | 0.0011 [0.0001, 0.0025] | 0.0004* [-0.0007, 0.0016] |
| Not Cheerful | 0.001 [-0.0003, 0.0025] | **0.0017 [0.0004, 0.0035]** | 0.0016 [0.0002, 0.0035] |
| Listless | 0.0002 [-0.0008, 0.0013] | -0.0001 [-0.0012, 0.0011] | 0.0004* [-0.0005, 0.0016] |
| Down | **0.0025 [0.0009, 0.0045]** | 0.0005 [-0.0003, 0.0019] | **0.0044 [0.002, 0.0073]** |
| Note: estimates accompanied by an asterisk (*) were significantly different from the bridge effect with the highest point estimate (bolded) within that group | | | |

| **Table S7.** Posterior means and 95% Credible Intervals (CI) for the mean bridge effect (i.e., bridge effect divided by the number of indirect effects) of each mental state in the comorbid group with a recent (<6-month) diagnosis, and the original depression-only and anxiety-only groups. | | | |
| --- | --- | --- | --- |
|  | Comorbidity group (n=75) | Depression-only group (n=40) | Anxiety-only group (n=37) |
| Mental state | Estimate [CI] | Estimate [CI] | Estimate [CI] |
| Not Relaxed | 0.0008* [-0.0004, 0.002] | 0.0005* [-0.0006, 0.0018] | 0.0004* [-0.0011, 0.0019] |
| Nervous | 0.0017 [0.0008, 0.0029] | 0.0001* [-0.0009, 0.0011] | 0.0006* [-0.0004, 0.0019] |
| Irritated | 0.0001* [-0.001, 0.0012] | 0 [-0.0005, 0.0006] | -0.0004* [-0.0019, 0.0008] |
| Worrying | 0.0018 [0.0009, 0.0029] | 0.0009 [0, 0.0022] | 0.0003* [-0.0007, 0.0014] |
| Not Cheerful | 0.0016 [0.0004, 0.0029] | **0.0016 [0.0004, 0.0033]** | 0.0016 [0.0001, 0.0033] |
| Listless | 0.0006* [-0.0003, 0.0016] | 0 [-0.0011, 0.0011] | 0.0003* [-0.0006, 0.0015] |
| Down | **0.0030 [0.0016, 0.0046]** | 0.0004 [-0.0003, 0.0016] | **0.0043 [0.0021, 0.0069]** |
| Note: estimates accompanied by an asterisk (*) were significantly different from the bridge effect with the highest point estimate (bolded) within that group | | | |

| **Table S8**. Posterior means and 95% Credible Intervals (CI) for the mean bridge effect (i.e., bridge effect divided by the number of indirect effects) of each mental state for the individuals without recent (i.e., 6-month diagnosis) in each outcome group. | | | |
| --- | --- | --- | --- |
|  | Comorbidity group (n=68) | Depression-only group (n=33) | Anxiety-only group (n=25) |
| Mental state | Estimate [CI] | Estimate [CI] | Estimate [CI] |
| Not Relaxed | 0.0007* [-0.0004, 0.0017] | 0.0004 [-0.0007, 0.0019] | 0.0004* [-0.0017, 0.0025] |
| Nervous | 0.0005* [-0.0004, 0.0014] | 0.0001 [-0.001, 0.0013] | 0.0007* [-0.0008, 0.0025] |
| Irritated | 0.0001* [-0.0006, 0.0009] | 0 [-0.0008, 0.0007] | -0.0005* [-0.0024, 0.0012] |
| Worrying | 0.0011 [0.0002, 0.0022] | 0.001 [0, 0.0026] | 0.0005* [-0.0013, 0.0024] |
| Not Cheerful | 0.0018 [0.0007, 0.0032] | **0.0014 [0.0001, 0.0032]** | 0.0018 [-0.0002, 0.0042] |
| Listless | 0* [-0.0007, 0.0009] | 0 [-0.001, 0.0011] | 0.0003 [-0.0012, 0.002] |
| Down | **0.0026 [0.0013, 0.0042]** | 0.0002 [-0.0008, 0.0015] | **0.0049 [0.0021, 0.0086]** |
| Note: estimates accompanied by an asterisk (*) were significantly different from the bridge effect with the highest point estimate (bolded) within that group | | | |

**Figure S1.** *Eight variable model.* Bridge effects with credible intervals (black lines) for the overlapping mental states ‘worrying’ and ‘feeling irritated’, in the comorbid, depression-only and anxiety-only groups. Bridge effects reflect the summed average within-person indirect effects for the mental state in each group.


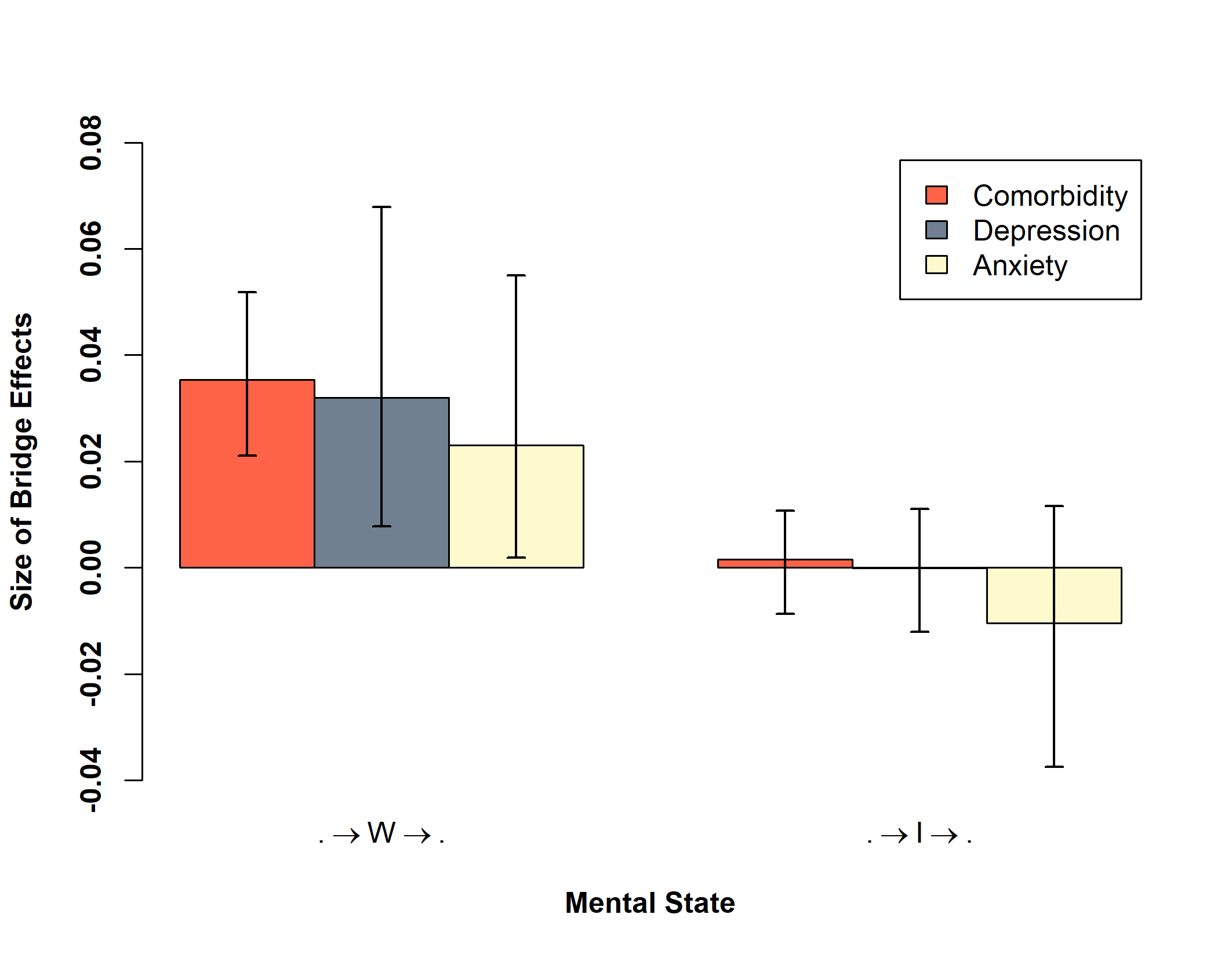


Worrying

Irritated

p = 0.22

p = 0.42

**Figure S2.** *Eight variable model.* Mean bridge effects (i.e., bridge effect divided by number of indirect effects) with credible intervals (black lines) for each of the eight mental states when treating that mental state as a bridge mental state, for the three groups separately.


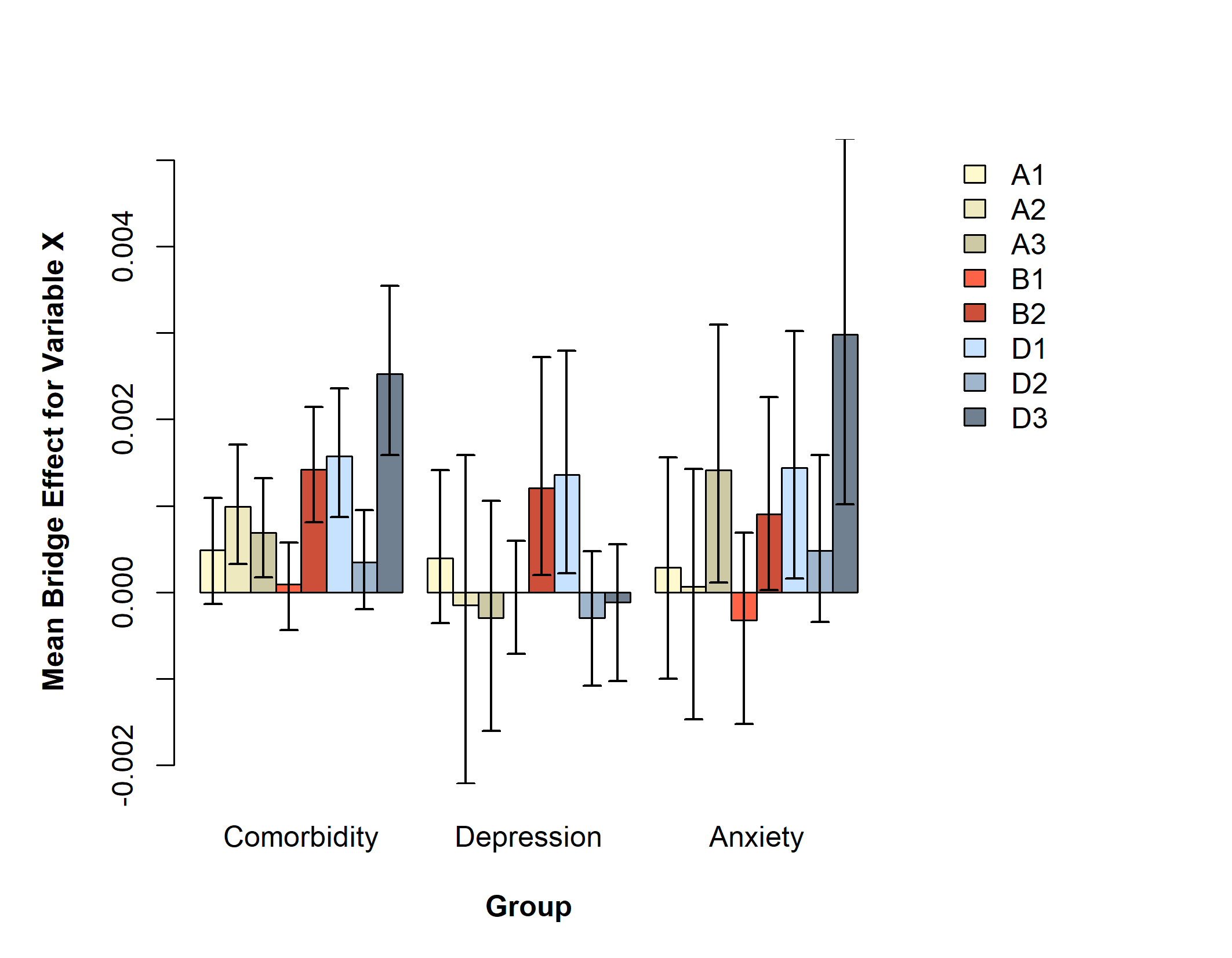


Not relaxed

Nervous

Anxious

Irritated

Worrying

Not cheerful

Listless

Down

FUCK DIT IS HET VERKEERDE FIGUUR>>>>>>>

**Figure S3.** *Detrended data.* Bridge effects with credible intervals (black lines) for the overlapping mental states ‘worrying’ and ‘feeling irritated’, in the comorbid, depression-only and anxiety-only groups obtained using prior-to-mplus detrended time-series. Bridge effects reflect the summed average within-person indirect effects for the mental state in each group.

**
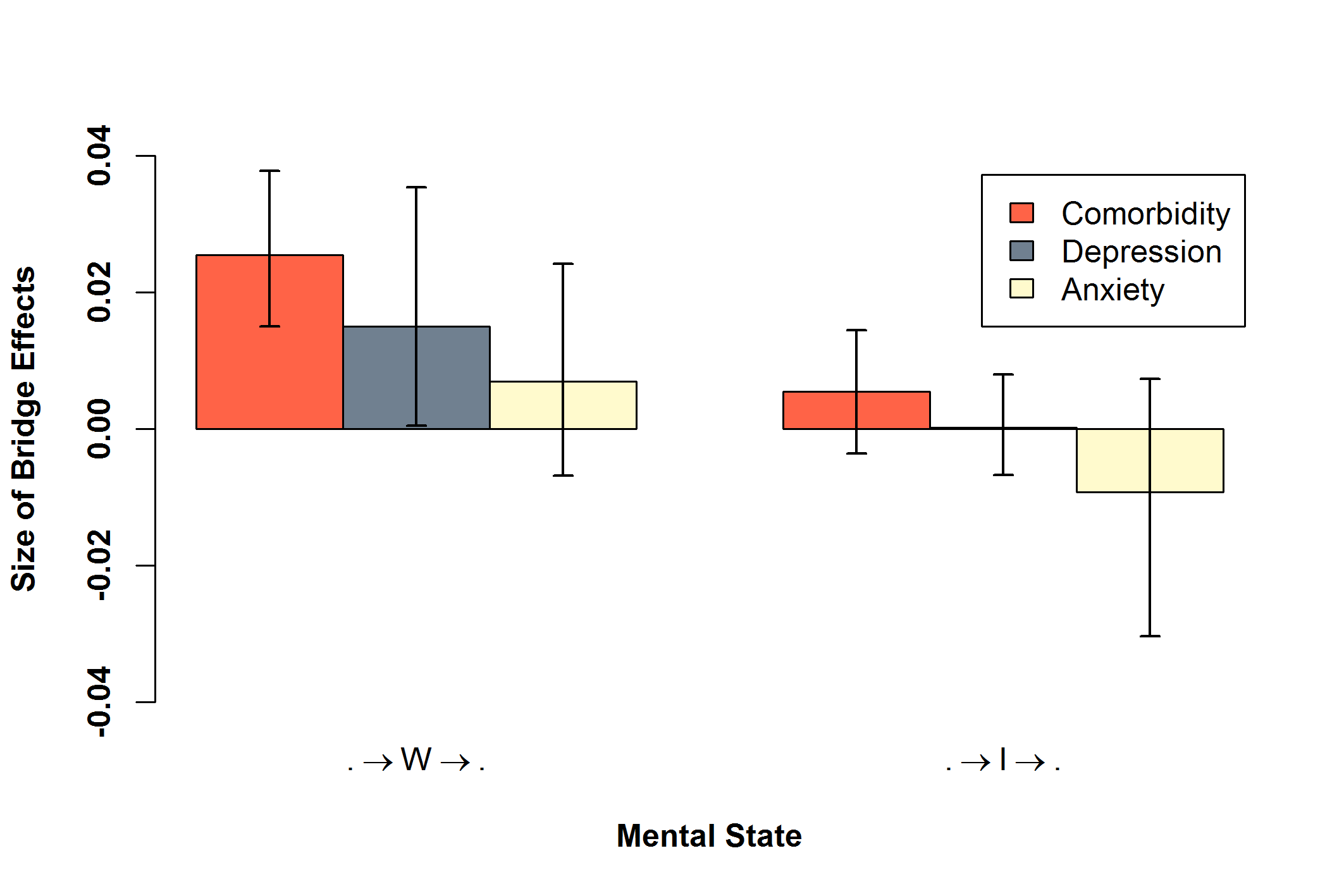
Figure S4.** *Detrended data.* Mean bridge effects (i.e., bridge effect divided by number of indirect effects) with credible intervals (black lines) for each of the seven mental states when treating that mental state as a bridge mental state, for the three groups separately. Results were obtained using prior-to-mplus detrended time-series.

p = 0.03

p = 0.17

Worrying

Irritated


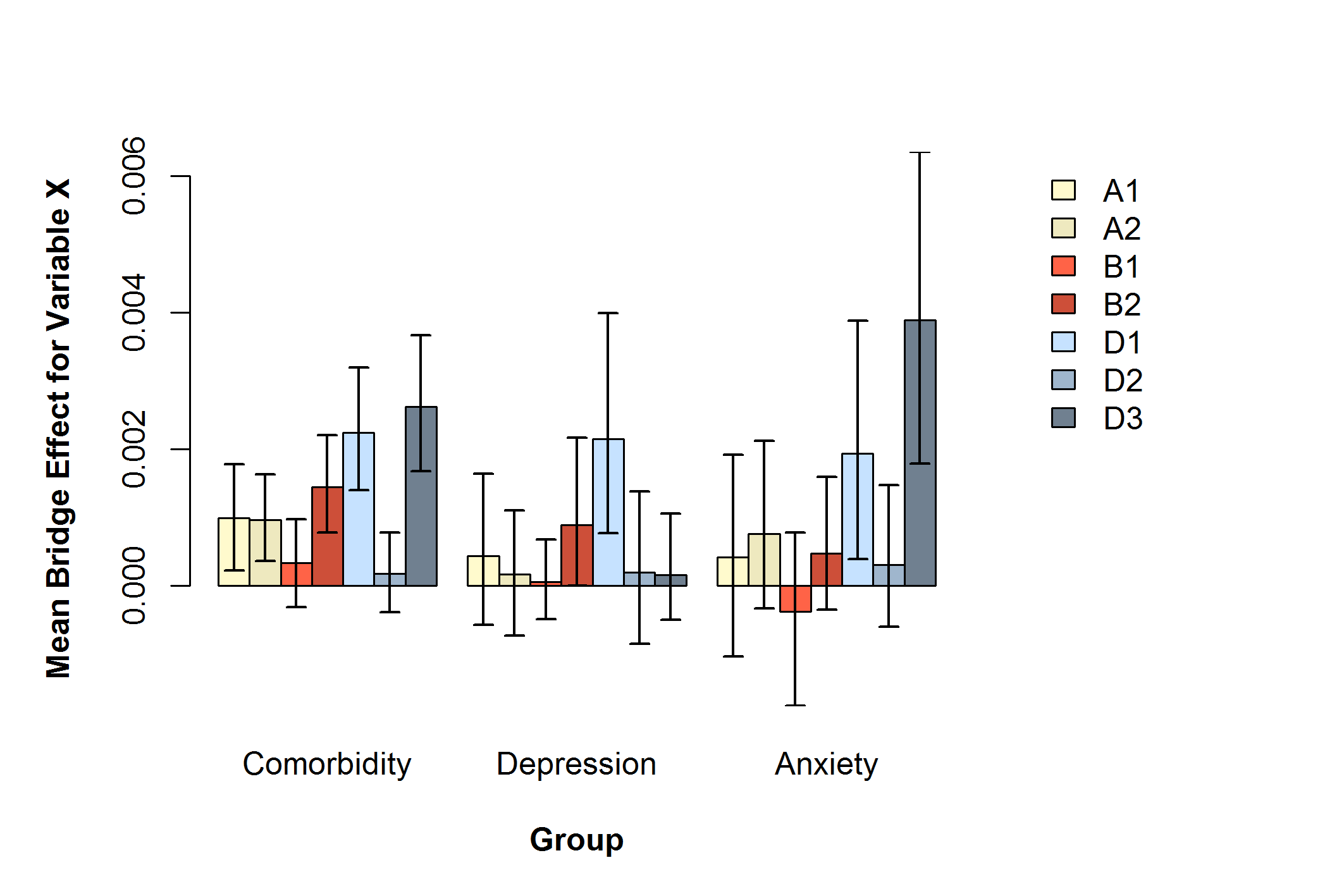


Not relaxed

Nervous

Irritated

Worrying

Not cheerful

Listless

Down

**Figure S5.** *Sensitivity Analysis 1.* Bridge effects with credible intervals (black lines) for the overlapping mental states ‘worrying’ and ‘feeling irritated’, in the lowest severity tertile of the comorbid group, and the original depression-only and anxiety-only groups. Bridge effects reflect the summed average within-person indirect effects for the mental state in each group.


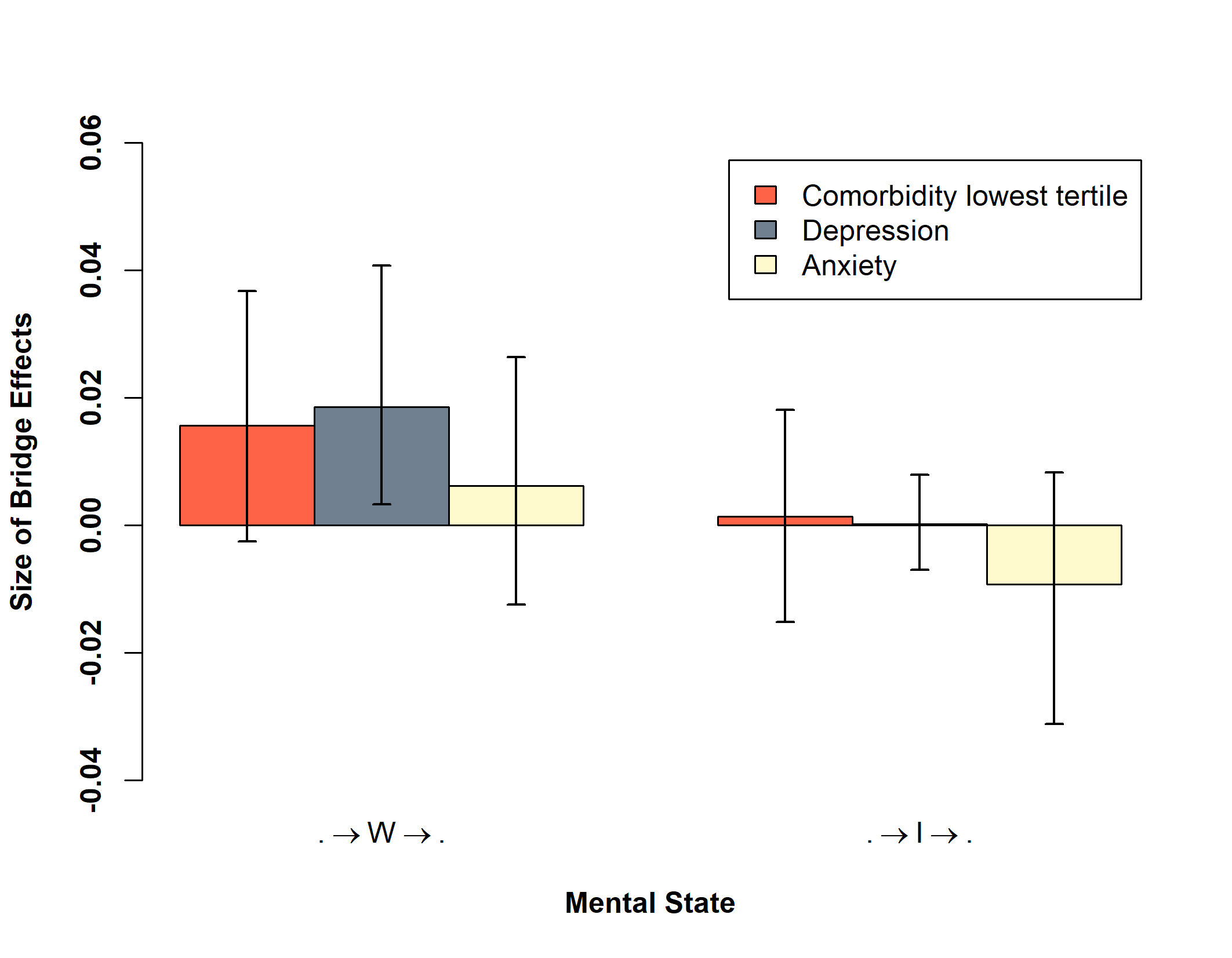


Worrying

Irritated

p = 0.24

p = 0.40

**Figure S6.** *Sensitivity Analysis 2.* Bridge effects with credible intervals (black lines) for the overlapping mental states ‘worrying’ and ‘feeling irritated’, in comorbid group with a recent (<6-month) diagnosis, and the original depression-only and anxiety-only groups. Bridge effects reflect the summed average within-person indirect effects for the mental state in each group.


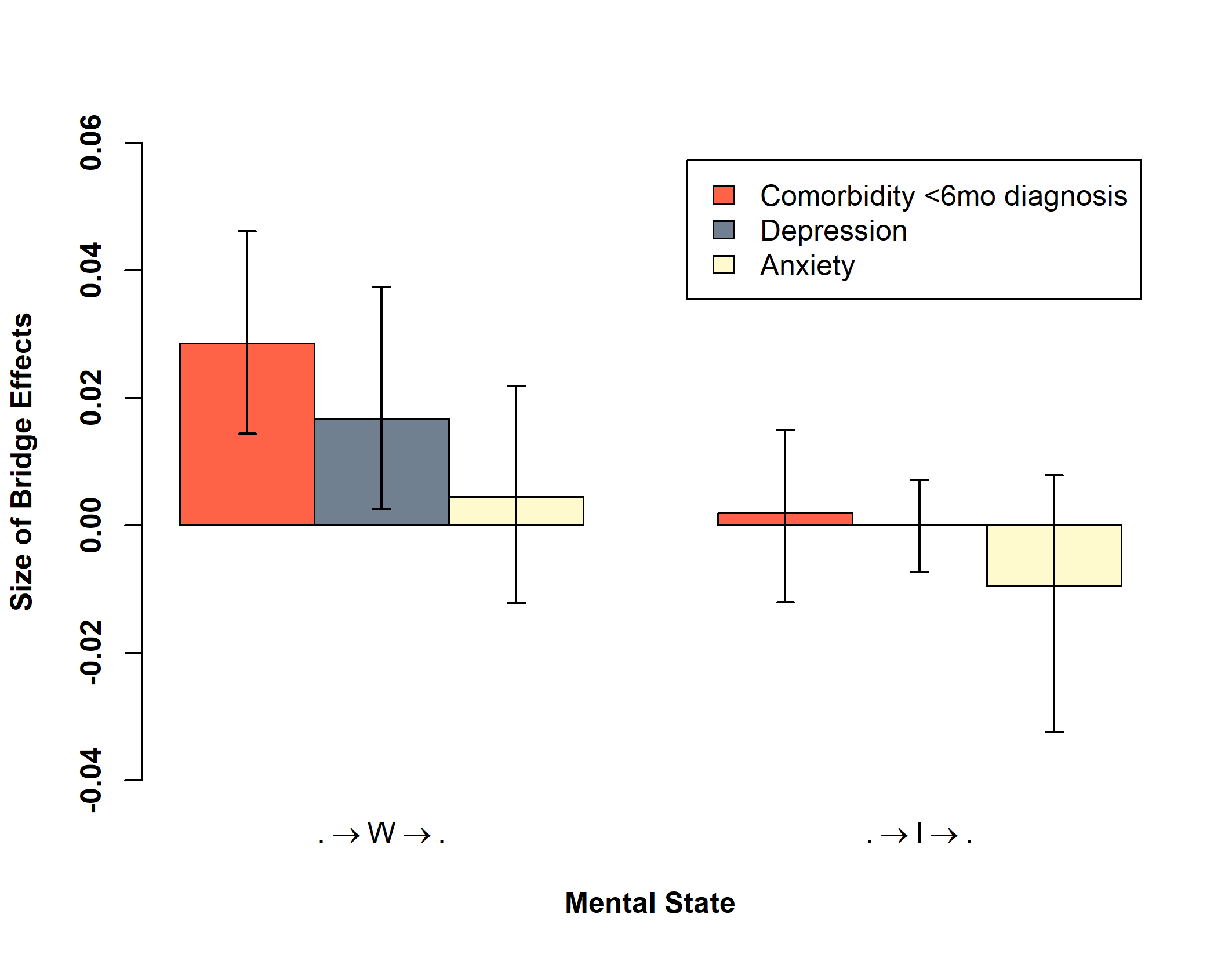


Irritated

Worrying

p = 0.02

p = 0.17

**Figure S7.** *Sensitivity Analysis 1.* Mean bridge effects (i.e., bridge effect divided by number of indirect effects) with credible intervals (black lines) for each of the mental states when treating that mental state as a bridge mental state, for the lowest tertile comorbidity group, depression-only and anxiety-only group.


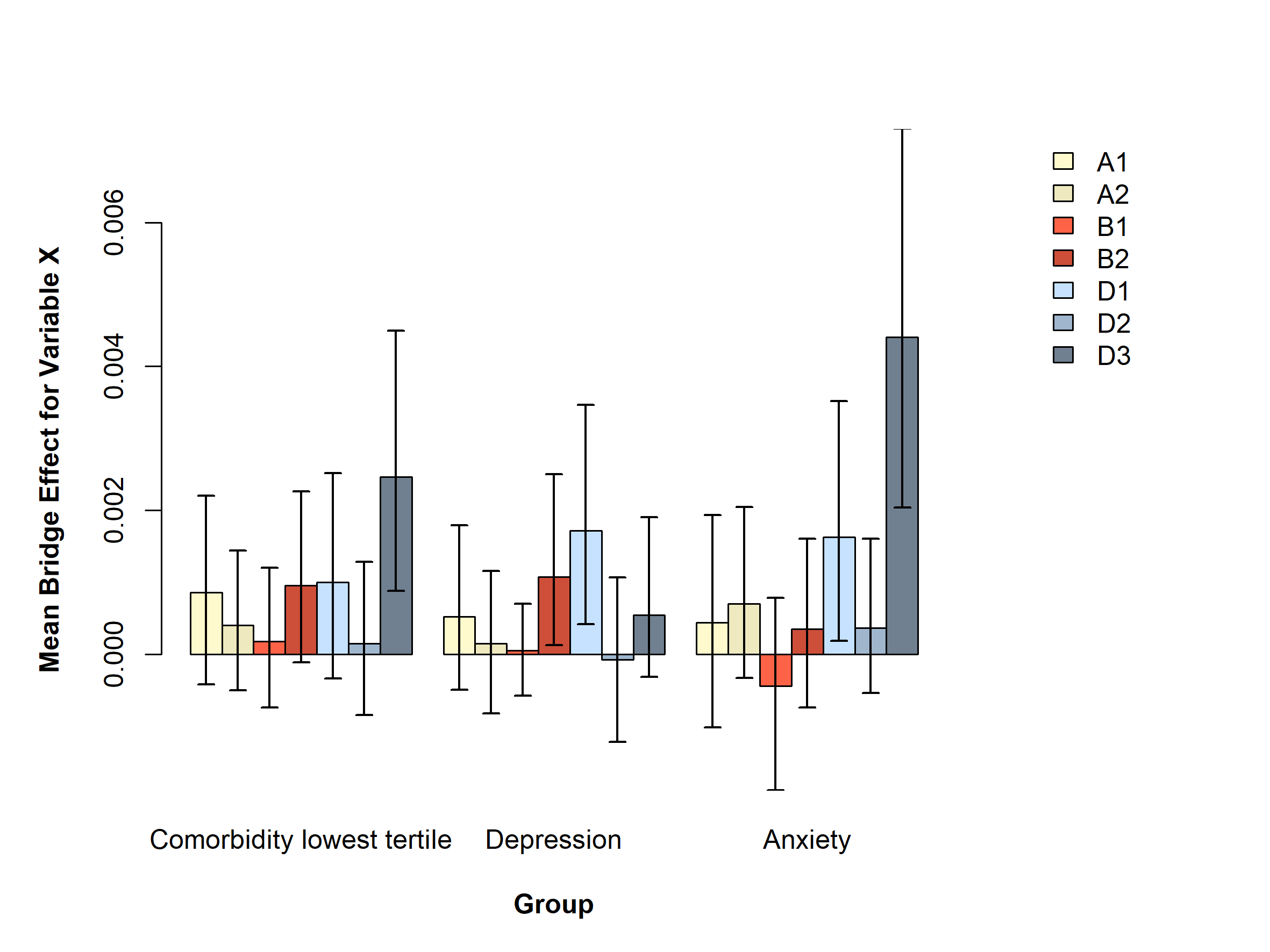


Not relaxed

Nervous

Irritated

Worrying

Not cheerful

Listless

Down

**Figure S8.** *Sensitivity Analysis 2.* Mean bridge effects (i.e., bridge effect divided by number of indirect effects) with credible intervals (black lines) for each of the mental states when treating that mental state as a bridge mental state, for participants with a recent (<6-month) diagnosis in the comorbidity group, and the original depression-only and anxiety-only groups.


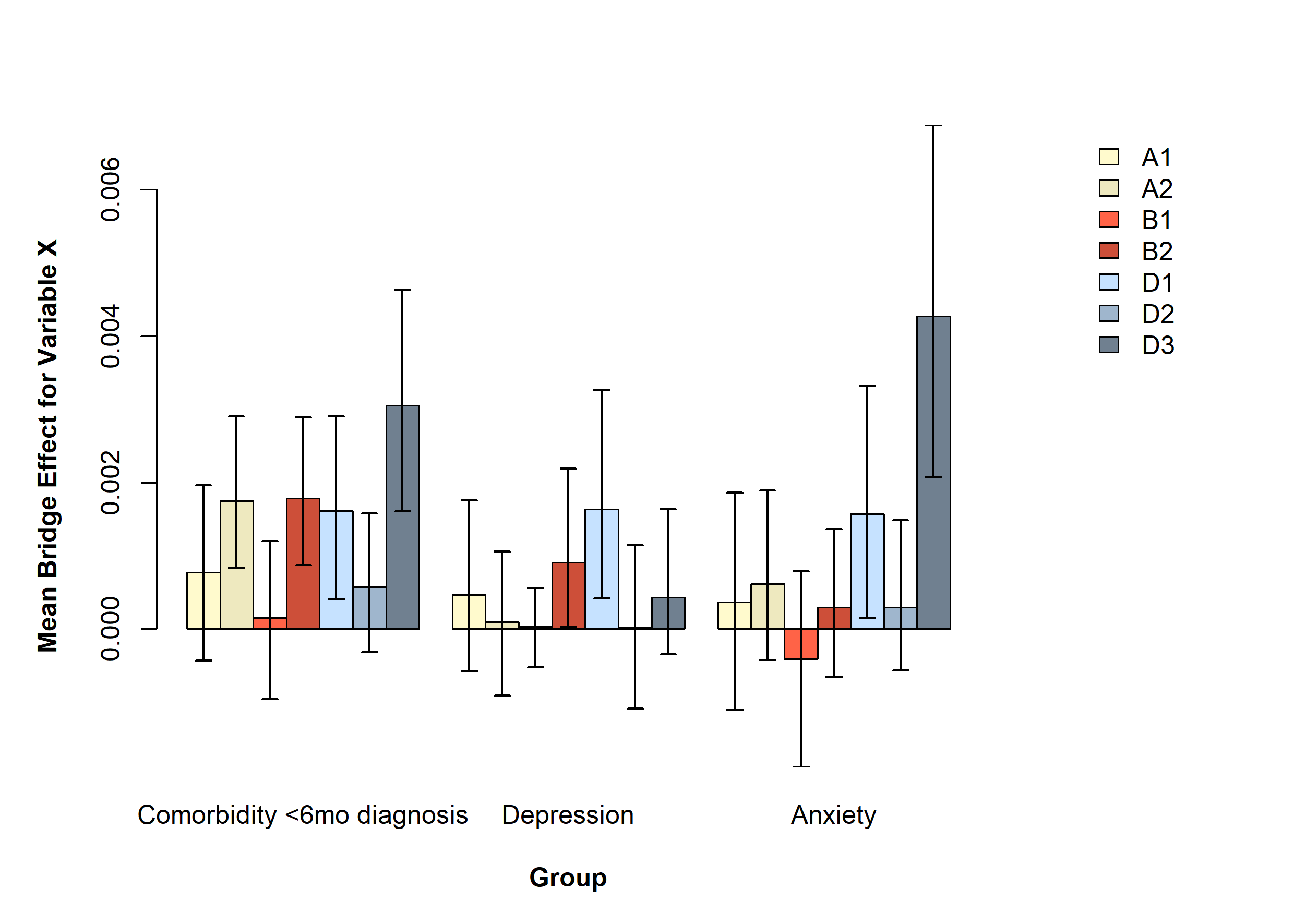


Not relaxed

Nervous

Irritated

Worrying

Not cheerful

Listless

Down

**Figure S9.** *Preregistered sensitivity analysis 2.* Bridge effects with credible intervals (black lines) for the overlapping mental states ‘worrying’ and ‘feeling irritated’, for the participants in the comorbidity, depression-only and anxiety-only groups without a recent (<6-month) diagnosis. Bridge effects reflect the summed average within-person indirect effects for the mental state in each group.


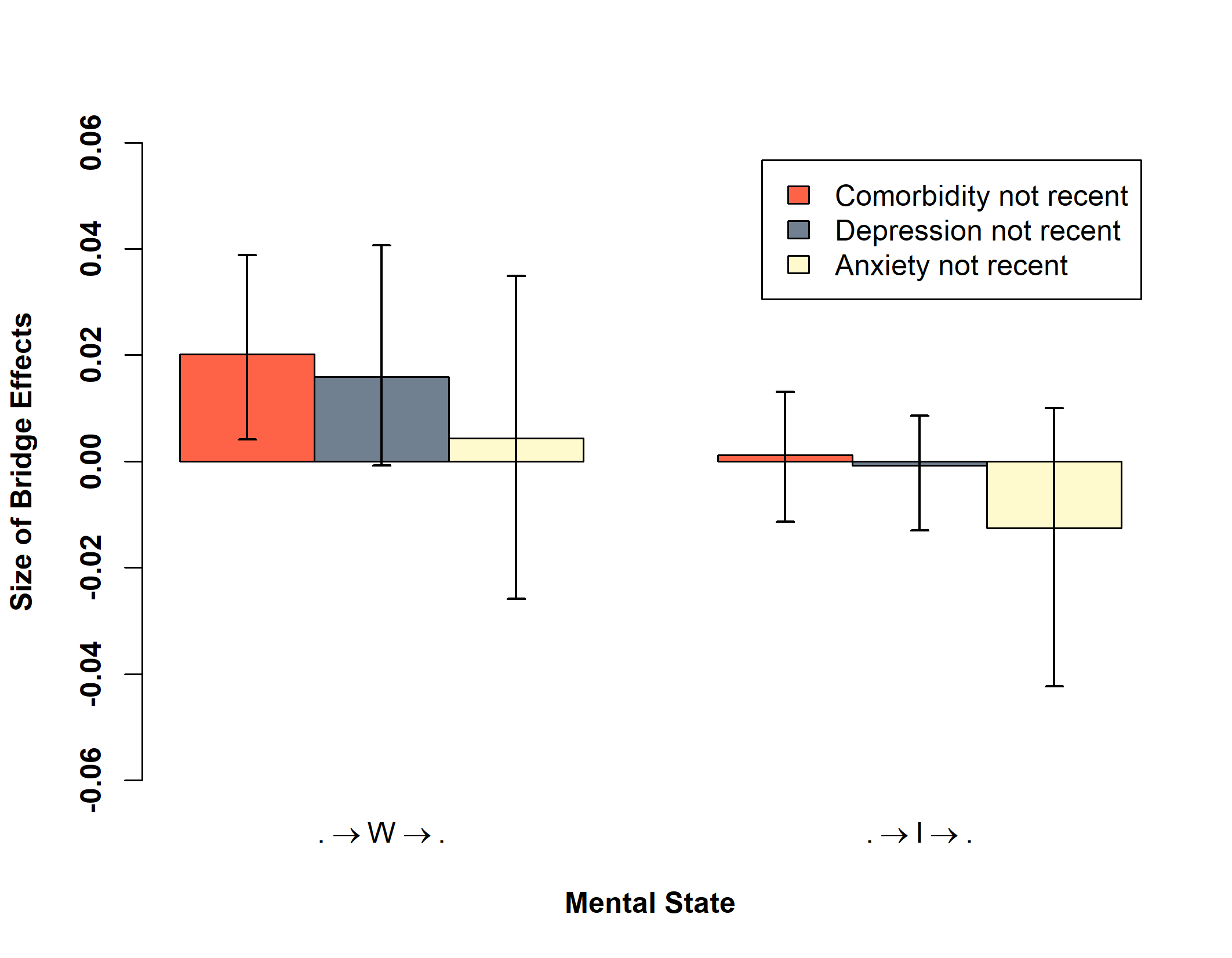


Worrying

Irritated

p = 0.17

p = 0.38

**Figure S10.** *Preregistered sensitivity analysis 2.* Mean bridge effects (i.e., bridge effect divided by number of indirect effects) with credible intervals (black lines) for each of the mental states when treating that mental state as a bridge mental state, for the participants in the comorbidity, depression-only and anxiety-only groups without a recent (<6-month) diagnosis.


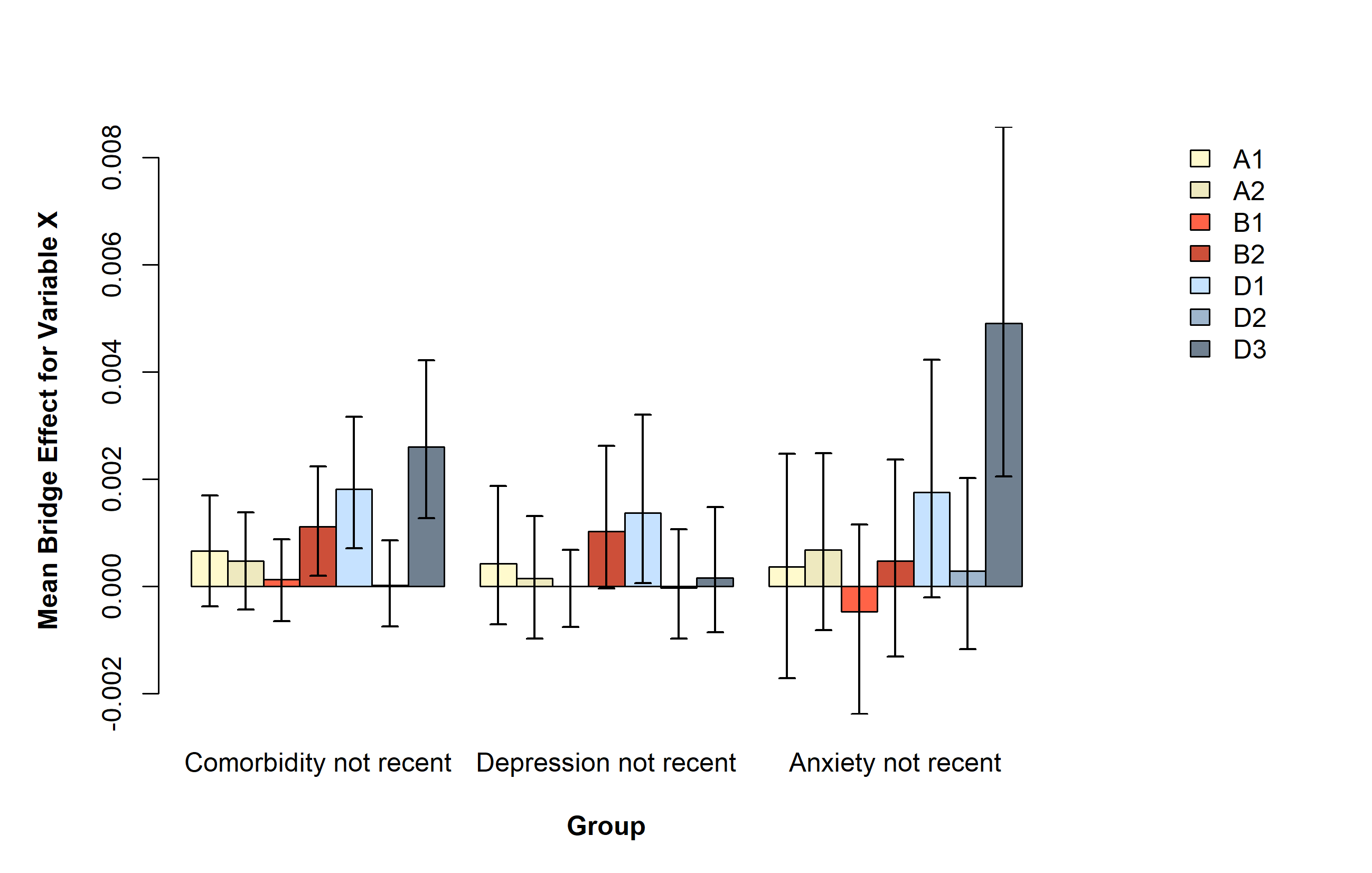


Not relaxed

Nervous

Irritated

Worrying

Not cheerful

Listless

Down
