# Supplementary material for: Comorbidity between depression and anxiety: assessing the role of bridge mental states in dynamic psychological networks
Source: BMC Med. 2020 Sep 29;18:308. doi: 10.1186/s12916-020-01738-z (PMC7523307; doi:10.1186/s12916-020-01738-z)
Supplement: Supplementary file 2 — Additional file 2. [file 12916_2020_1738_MOESM2_ESM.pdf]

ADDITIONAL FILE 2 – Mplus syntax main analysis

Comorbidity between depression and anxiety: assessing the role of bridge mental states in dynamic psychological networks.

Robin. N. Groen , Oisín Ryan, Johanna T.W. Wigman, Harriette Riese, Brenda W.J.H. Penninx, Erik J. Giltay, Marieke Wichers, Catharina A. Hartman

MPLUS SYNTAX – Main analysis

```
TITLE:    NESDA EMA comorbidity depression and anxiety bridge
  hypothesis - 7 variables main analysis.
  Variables standardized prior to Mplus ;
DATA:   FILE = "ema_std_7v_main.dat"; ! Important which file is used!
VARIABLE:          NAMES = pident outcome_group time_h time_m D_AC D_DC anx1
dep1 bridge1 dep2 dep3 anx2 bridge2;

MISSING=.;
CLUSTER     = pident;
usevar      =    anx1 dep1 bridge1 dep2 dep3 anx2 bridge2 D_DC D_AC;
between     = D_AC D_DC;
missing     =  all(-999);
lagged      = anx1 dep1 bridge1 dep2 dep3 anx2 bridge2 (1);
tinterval   =   time_m(180);

ANALYSIS: TYPE IS TWOLEVEL RANDOM; ! This allows for random slopes
ESTIMATOR = BAYES; ! DSEM requires Bayesian estimation
PROC = 3; ! Using 2 processors makes it faster
FBITER = (40000); !
THIN = 4;
BSEED = 1234; ! brackets allows extra iterations for convergence

MODEL:            %WITHIN%! Specify the (random) lagged relationships

A1A1 | anx1 ON anx1&1;
A1D1      | anx1 ON dep1&1; !anx1 is predicted by dep1
A1B1     | anx1 ON bridge1&1; !anx1 is predicted by bridge1
A1D2          | anx1 ON dep2&1;
A1D3      | anx1 ON dep3&1;
A1A2       | anx1 ON anx2&1;
A1B2      | anx1 ON bridge2&1;

D1A1     | dep1 ON anx1&1; !dep1 is predicted by anx1
D1D1       | dep1 ON dep1&1;
D1B1     | dep1 ON bridge1&1;
D1D2          | dep1 ON dep2&1;
D1D3           | dep1 ON dep3&1;
D1A2       | dep1 ON anx2&1;
D1B2      | dep1 ON bridge2&1;

B1A1 | bridge1 ON anx1&1; ! bridge 1 is predicted by anx1
B1D1      | bridge1 ON dep1&1;
B1B1     | bridge1 ON bridge1&1;
B1D2          | bridge1 ON dep2&1;
B1D3      | bridge1 ON dep3&1;
B1A2       | bridge1 ON anx2&1;
```

```
B1B2       | bridge1 ON bridge2&1;

D2A1 | dep2 ON anx1&1;
D2D1      | dep2 ON dep1&1;
D2B1     | dep2 ON bridge1&1;
D2D2          | dep2 ON dep2&1;
D2D3           | dep2 ON dep3&1;
D2A2       | dep2 ON anx2&1;
D2B2      | dep2 ON bridge2&1;

D3A1 | dep3 ON anx1&1;
D3D1      | dep3 ON dep1&1;
D3B1     | dep3 ON bridge1&1;
D3D2          | dep3 ON dep2&1;
D3D3      | dep3 ON dep3&1;
D3A2         | dep3 ON anx2&1;
D3B2      | dep3 ON bridge2&1;

A2A1 | anx2 ON anx1&1; !anx2 is predicted by anx1
A2D1      | anx2 ON dep1&1;
A2B1     | anx2 ON bridge1&1;
A2D2          | anx2 ON dep2&1;
A2D3      | anx2 ON dep3&1;
A2A2 | anx2 ON anx2&1;
A2B2      | anx2 ON bridge2&1;


B2A1 | bridge2 ON anx1&1;
B2D1      | bridge2 ON dep1&1; !anx1 is predicted by dep1
B2B1     | bridge2 ON bridge1&1; !anx1 is predicted by bridge1
B2D2          | bridge2 ON dep2&1;
B2D3      | bridge2 ON dep3&1;
B2A2       | bridge2 ON anx2&1;
B2B2      | bridge2 ON bridge2&1;



 %BETWEEN%

anx1 dep1 bridge1 dep2 dep3 anx2 bridge2 A1A1-B2B2; ! specify all variances

  anx1  WITH dep1 bridge1 dep2 dep3 anx2  bridge2; ! covariances random intercept
  dep1  WITH bridge1 dep2 dep3 anx2  bridge2; ! covariances random intercept
  bridge1 WITH dep2 dep3 anx2  bridge2; ! covariances random intercept
  dep2    WITH dep3 anx2  bridge2; ! covariances random intercept
  dep3    WITH anx2  bridge2; ! covariances random intercept
  anx2    WITH  bridge2; ! covariances random intercept
```

```
[A1A1 ] (A1A1_0);
A1A1  ON D_DC (A1A1_r1);
A1A1  ON D_AC (A1A1_r2);
[A1D1 ] (A1D1_0);
A1D1  ON D_DC (A1D1_r1);
A1D1  ON D_AC (A1D1_r2);
[A1B1 ] (A1B1_0);
A1B1  ON D_DC (A1B1_r1);
A1B1  ON D_AC (A1B1_r2);
[A1D2 ] (A1D2_0);
A1D2  ON D_DC (A1D2_r1);
A1D2  ON D_AC (A1D2_r2);
[A1D3 ] (A1D3_0);
A1D3  ON D_DC (A1D3_r1);
A1D3  ON D_AC (A1D3_r2);
[A1A2 ] (A1A2_0);
A1A2  ON D_DC (A1A2_r1);
A1A2  ON D_AC (A1A2_r2);
[A1B2 ] (A1B2_0);
A1B2  ON D_DC (A1B2_r1);
A1B2  ON D_AC (A1B2_r2);

[D1A1 ] (D1A1_0);
D1A1  ON D_DC (D1A1_r1);
D1A1  ON D_AC (D1A1_r2);
[D1D1 ] (D1D1_0);
D1D1  ON D_DC (D1D1_r1);
D1D1  ON D_AC (D1D1_r2);
[D1B1 ] (D1B1_0);
D1B1  ON D_DC (D1B1_r1);
D1B1  ON D_AC (D1B1_r2);
[D1D2 ] (D1D2_0);
D1D2  ON D_DC (D1D2_r1);
D1D2  ON D_AC (D1D2_r2);
[D1D3 ] (D1D3_0);
D1D3  ON D_DC (D1D3_r1);
D1D3  ON D_AC (D1D3_r2);
[D1A2 ] (D1A2_0);
D1A2  ON D_DC (D1A2_r1);
D1A2  ON D_AC (D1A2_r2);
[D1B2 ] (D1B2_0);
D1B2  ON D_DC (D1B2_r1);
D1B2  ON D_AC (D1B2_r2);
```

```
[B1A1 ] (B1A1_0);
B1A1  ON D_DC (B1A1_r1);
B1A1  ON D_AC (B1A1_r2);
[B1D1  ] (B1D1_0);
B1D1  ON D_DC (B1D1_r1);
B1D1  ON D_AC (B1D1_r2);
[B1B1 ] (B1B1_0);
B1B1  ON D_DC (B1B1_r1);
B1B1  ON D_AC (B1B1_r2);
[B1D2 ] (B1D2_0);
B1D2  ON D_DC (B1D2_r1);
B1D2  ON D_AC (B1D2_r2);
[B1D3 ] (B1D3_0);
B1D3  ON D_DC (B1D3_r1);
B1D3  ON D_AC (B1D3_r2);
[B1A2 ] (B1A2_0);
B1A2  ON D_DC (B1A2_r1);
B1A2  ON D_AC (B1A2_r2);
[B1B2 ] (B1B2_0);
B1B2  ON D_DC (B1B2_r1);
B1B2  ON D_AC (B1B2_r2);


[D2A1 ] (D2A1_0);
D2A1  ON D_DC (D2A1_r1);
D2A1  ON D_AC (D2A1_r2);
[D2D1  ] (D2D1_0);
D2D1  ON D_DC (D2D1_r1);
D2D1  ON D_AC (D2D1_r2);
[D2B1] (D2B1_0);
D2B1 ON D_DC (D2B1_r1);
D2B1 ON D_AC (D2B1_r2);
[D2D2 ] (D2D2_0);
D2D2  ON D_DC (D2D2_r1);
D2D2  ON D_AC (D2D2_r2);
[D2D3 ] (D2D3_0);
D2D3  ON D_DC (D2D3_r1);
D2D3  ON D_AC (D2D3_r2);
[D2A2 ] (D2A2_0);
D2A2  ON D_DC (D2A2_r1);
D2A2  ON D_AC (D2A2_r2);
[D2B2 ] (D2B2_0);
D2B2  ON D_DC (D2B2_r1);
D2B2  ON D_AC (D2B2_r2);

[D3A1 ] (D3A1_0);
D3A1  ON D_DC (D3A1_r1);
D3A1  ON D_AC (D3A1_r2);
```

[D3D1] (D3D1_0);
D3D1 ON D_DC (D3D1_r1);
D3D1 ON D_AC (D3D1_r2);
[D3B1] (D3B1_0);
D3B1 ON D_DC (D3B1_r1);
D3B1 ON D_AC (D3B1_r2);
[D3D2] (D3D2_0);
D3D2 ON D_DC (D3D2_r1);
D3D2 ON D_AC (D3D2_r2);
[D3D3] (D3D3_0);
D3D3 ON D_DC (D3D3_r1);
D3D3 ON D_AC (D3D3_r2);
[D3A2] (D3A2_0);
D3A2 ON D_DC (D3A2_r1);
D3A2 ON D_AC (D3A2_r2);
[D3B2] (D3B2_0);
D3B2 ON D_DC (D3B2_r1);
D3B2 ON D_AC (D3B2_r2);

[A2A1] (A2A1_0);
A2A1 ON D_DC (A2A1_r1);
A2A1 ON D_AC (A2A1_r2);
[A2D1] (A2D1_0);
A2D1 ON D_DC (A2D1_r1);
A2D1 ON D_AC (A2D1_r2);
[A2B1] (A2B1_0);
A2B1 ON D_DC (A2B1_r1);
A2B1 ON D_AC (A2B1_r2);
[A2D2] (A2D2_0);
A2D2 ON D_DC (A2D2_r1);
A2D2 ON D_AC (A2D2_r2);
[A2D3] (A2D3_0);
A2D3 ON D_DC (A2D3_r1);
A2D3 ON D_AC (A2D3_r2);
[A2A2] (A2A2_0);
A2A2 ON D_DC (A2A2_r1);
A2A2 ON D_AC (A2A2_r2);
[A2B2] (A2B2_0);
A2B2 ON D_DC (A2B2_r1);
A2B2 ON D_AC (A2B2_r2);

[B2A1] (B2A1_0);
B2A1 ON D_DC (B2A1_r1);
B2A1 ON D_AC (B2A1_r2);
[B2D1] (B2D1_0);
B2D1 ON D_DC (B2D1_r1);
B2D1 ON D_AC (B2D1_r2);
[B2B1] (B2B1_0);

```
B2B1 ON D_DC (B2B1_r1);
B2B1 ON D_AC (B2B1_r2);
[B2D2] (B2D2_0);
B2D2  ON D_DC (B2D2_r1);
B2D2 ON D_AC (B2D2_r2);
[B2D3] (B2D3_0);
B2D3  ON D_DC (B2D3_r1);
B2D3 ON D_AC (B2D3_r2);
[B2A2 ] (B2A2_0);
B2A2  ON D_DC (B2A2_r1);
B2A2  ON D_AC (B2A2_r2);
[B2B2 ] (B2B2_0);
B2B2  ON D_DC (B2B2_r1);
B2B2  ON D_AC (B2B2_r2);


Model Constraint:
! calculate seperate indirect effects Worrying for all three groups
! comorbid group
NEW(iB2_AD_0 iB2_DA_0 s_iB2_0);
iB2_AD_0 =  ((B2A1_0*D1B2_0)+ (B2A2_0*D1B2_0) +
(B2A1_0*D2B2_0)+ (B2A2_0*D2B2_0)+
(B2A1_0*D3B2_0)+ (B2A2_0*D3B2_0))*1000; ! indirect effect Anxiety to Bridge 2 to Dep
iB2_DA_0 =  ((B2D1_0*A1B2_0)+ (B2D2_0*A1B2_0)+ (B2D3_0*A1B2_0) +
(B2D1_0*A2B2_0)+ (B2D2_0*A2B2_0)+ (B2D3_0*A2B2_0))*1000; ! indirect effect Dep to Bridge 2 to
Anxiety
  ! indirect effect DEP to Bridge to ANX
s_iB2_0 = iB2_AD_0 + iB2_DA_0;


! depression group
NEW(iB2_AD_1 iB2_DA_1 s_iB2_1);
iB2_AD_1 = (((B2A1_0+B2A1_r1) *(D1B2_0+D1B2_r1))+
((B2A2_0+ B2A2_r1)*(D1B2_0 + D1B2_r1))+ ((B2A1_0+ B2A1_r1)*(D2B2_0+ D2B2_r1))
+ ((B2A2_0+B2A2_r1) *(D2B2_0+D2B2_r1))
 + ((B2A1_0+ B2A1_r1)*(D3B2_0+ D3B2_r1))+
((B2A2_0+ B2A2_r1)*(D3B2_0+ D3B2_r1)))*1000;
iB2_DA_1 = (((B2D1_0+B2D1_r1)*(A1B2_0+A1B2_r1)) +
((B2D2_0+ B2D2_r1)*(A1B2_0 + A1B2_r1))+ ((B2D3_0+ B2D3_r1)*(A1B2_0+ A1B2_r1)) +
((B2D1_0+ B2D1_r1)*(A2B2_0+ A2B2_r1))+ ((B2D2_0+B2D2_r1) *(A2B2_0+A2B2_r1))+
((B2D3_0+ B2D3_r1)*(A2B2_0+ A2B2_r1)))*1000;
s_iB2_1 = iB2_AD_1 + iB2_DA_1;

! anxiety group
NEW(iB2_AD_2 iB2_DA_2 s_iB2_2);
iB2_AD_2 = (((B2A1_0+B2A1_r2) *(D1B2_0+D1B2_r2))+
((B2A2_0+ B2A2_r2)*(D1B2_0 + D1B2_r2))
+ ((B2A1_0+ B2A1_r2)*(D2B2_0+ D2B2_r2))+ ((B2A2_0+B2A2_r2) *(D2B2_0+D2B2_r2))+
 ((B2A1_0+ B2A1_r2)*(D3B2_0+ D3B2_r2))+
```

```
((B2A2_0+ B2A2_r2)*(D3B2_0+ D3B2_r2)))*1000;
iB2_DA_2 = (((B2D1_0+B2D1_r2)*(A1B2_0+A1B2_r2)) +
((B2D2_0+ B2D2_r2)*(A1B2_0 + A1B2_r2))+ ((B2D3_0+ B2D3_r2)*(A1B2_0+ A1B2_r2)) +
((B2D1_0+ B2D1_r2)*(A2B2_0+ A2B2_r2))+ ((B2D2_0+B2D2_r2) *(A2B2_0+A2B2_r2))+
((B2D3_0+ B2D3_r2)*(A2B2_0+ A2B2_r2)))*1000;
s_iB2_2 = iB2_AD_2 + iB2_DA_2;



! Group differences seperate total indirect effects Worry
NEW(B2_AD_01 B2_AD_02 B2_AD_12 B2_DA_01 B2_DA_02 B2_DA_12 B2_s_01 B2_s_02 B2_s_12) ;

B2_AD_01 = iB2_AD_0 - iB2_AD_1;
B2_AD_02 = iB2_AD_0 - iB2_AD_2;
B2_AD_12 = iB2_AD_1 - iB2_AD_2;
B2_DA_01= iB2_DA_0 - iB2_DA_1;
B2_DA_02= iB2_DA_0 - iB2_DA_2;
B2_DA_12= iB2_DA_1 - iB2_DA_2;
B2_s_01 = s_iB2_0-s_iB2_1;
B2_s_02 = s_iB2_0 -s_iB2_2 ;
B2_s_12 = s_iB2_1 -s_iB2_2 ;


! calculate seperate indirect effects Irritated for all three groups
! comorbid group
NEW(iB1_AD_0 iB1_DA_0 s_iB1_0);
iB1_AD_0 =  ((B1A1_0*D1B1_0)+ (B1A2_0*D1B1_0)+
(B1A1_0*D2B1_0)+ (B1A2_0*D2B1_0) +
(B1A1_0*D3B1_0)+ (B1A2_0*D3B1_0))*1000; ! indirect effect Anxiety to Bridge 1 to Dep
iB1_DA_0 =  ((B1D1_0*A1B1_0)+ (B1D2_0*A1B1_0)+ (B1D3_0*A1B1_0) +
(B1D1_0*A2B1_0)+ (B1D2_0*A2B1_0)+ (B1D3_0*A2B1_0))*1000; ! indirect effect Dep to Bridge 1 to
Anxiety
 ! indirect effect DEP to Bridge to ANX
s_iB1_0 = iB1_AD_0 + iB1_DA_0;

! depression group
NEW(iB1_AD_1 iB1_DA_1 s_iB1_1);
iB1_AD_1 = (((B1A1_0+B1A1_r1) *(D1B1_0+D1B1_r1))+
((B1A2_0+ B1A2_r1)*(D1B1_0 + D1B1_r1))
+ ((B1A1_0+ B1A1_r1)*(D2B1_0+ D2B1_r1))+ ((B1A2_0+B1A2_r1) *(D2B1_0+D2B1_r1))+
 ((B1A1_0+ B1A1_r1)*(D3B1_0+ D3B1_r1))+
((B1A2_0+ B1A2_r1)*(D3B1_0+ D3B1_r1)) )*1000;
iB1_DA_1 = (((B1D1_0+B1D1_r1)*(A1B1_0+A1B1_r1)) +
((B1D2_0+ B1D2_r1)*(A1B1_0 + A1B1_r1))+ ((B1D3_0+ B1D3_r1)*(A1B1_0+ A1B1_r1)) +
((B1D1_0+ B1D1_r1)*(A2B1_0+ A2B1_r1))+ ((B1D2_0+B1D2_r1) *(A2B1_0+A2B1_r1))+
((B1D3_0+ B1D3_r1)*(A2B1_0+ A2B1_r1)) )*1000;
s_iB1_1 = iB1_AD_1 + iB1_DA_1;

! anxiety group
```

```
NEW(iB1_AD_2 iB1_DA_2 s_iB1_2);
iB1_AD_2 = (((B1A1_0+B1A1_r2) *(D1B1_0+D1B1_r2))+
((B1A2_0+ B1A2_r2)*(D1B1_0 + D1B1_r2))
+ ((B1A1_0+ B1A1_r2)*(D2B1_0+ D2B1_r2))+ ((B1A2_0+B1A2_r2) *(D2B1_0+D2B1_r2))+
 ((B1A1_0+ B1A1_r2)*(D3B1_0+ D3B1_r2))+
((B1A2_0+ B1A2_r2)*(D3B1_0+ D3B1_r2)))*1000;
iB1_DA_2 = (((B1D1_0+B1D1_r2)*(A1B1_0+A1B1_r2)) +
((B1D2_0+ B1D2_r2)*(A1B1_0 + A1B1_r2))+ ((B1D3_0+ B1D3_r2)*(A1B1_0+ A1B1_r2)) +
((B1D1_0+ B1D1_r2)*(A2B1_0+ A2B1_r2))+ ((B1D2_0+B1D2_r2) *(A2B1_0+A2B1_r2))+
((B1D3_0+ B1D3_r2)*(A2B1_0+ A2B1_r2)) )*1000;
s_iB1_2 = iB1_AD_2 + iB1_DA_2;

! Group differences seperate total indirect effects Irritated
NEW(B1_AD_01 B1_AD_02 B1_AD_12
 B1_DA_01 B1_DA_02 B1_DA_12
 B1_s_01 B1_s_02 B1_s_12);

B1_AD_01 = iB1_AD_0 - iB1_AD_1;
B1_AD_02 = iB1_AD_0 - iB1_AD_2;
B1_AD_12 = iB1_AD_1 - iB1_AD_2;
B1_DA_01= iB1_DA_0 - iB1_DA_1;
B1_DA_02= iB1_DA_0 - iB1_DA_2;
B1_DA_12= iB1_DA_1 - iB1_DA_2;
B1_s_01 = s_iB1_0-s_iB1_1;
B1_s_02 = s_iB1_0 -s_iB1_2 ;
B1_s_12 = s_iB1_1 -s_iB1_2 ;


 !Fixed Effects group 1 ;
 NEW(A1A1_1  A1A2_1
  A1D1_1  A1D2_1  A1D3_1  A1B1_1
  A1B2_1  A2A1_1  A2A2_1
  A2D1_1  A2D2_1  A2D3_1  A2B1_1
  A2B2_1  D1A1_1  D1A2_1
  D1D1_1  D1D2_1  D1D3_1  D1B1_1
  D1B2_1  D2A1_1  D2A2_1
  D2D1_1  D2D2_1  D2D3_1  D2B1_1
  D2B2_1  D3A1_1  D3A2_1
  D3D1_1  D3D2_1  D3D3_1  D3B1_1
  D3B2_1  B1A1_1  B1A2_1
  B1D1_1  B1D2_1  B1D3_1  B1B1_1
  B1B2_1  B2A1_1  B2A2_1
  B2D1_1  B2D2_1  B2D3_1  B2B1_1
  B2B2_1);

  A1A1_1 = A1A1_0 + A1A1_r1;
  A1A2_1 = A1A2_0 + A1A2_r1;
  A1D1_1 = A1D1_0 + A1D1_r1;
  A1D2_1 = A1D2_0 + A1D2_r1;
```

```
A1D3_1 = A1D3_0 + A1D3_r1;
A1B1_1 = A1B1_0 + A1B1_r1;
A1B2_1 = A1B2_0 + A1B2_r1;

A2A1_1 = A2A1_0 + A2A1_r1;
A2A2_1 = A2A2_0 + A2A2_r1;
A2D1_1 = A2D1_0 + A2D1_r1;
A2D2_1 = A2D2_0 + A2D2_r1;
A2D3_1 = A2D3_0 + A2D3_r1;
A2B1_1 = A2B1_0 + A2B1_r1;
A2B2_1 = A2B2_0 + A2B2_r1;

D1A1_1 = D1A1_0 + D1A1_r1;
D1A2_1 = D1A2_0 + D1A2_r1;
D1D1_1 = D1D1_0 + D1D1_r1;
D1D2_1 = D1D2_0 + D1D2_r1;
D1D3_1 = D1D3_0 + D1D3_r1;
D1B1_1 = D1B1_0 + D1B1_r1;
D1B2_1 = D1B2_0 + D1B2_r1;

D2A1_1 = D2A1_0 + D2A1_r1;
D2A2_1 = D2A2_0 + D2A2_r1;
D2D1_1 = D2D1_0 + D2D1_r1;
D2D2_1 = D2D2_0 + D2D2_r1;
D2D3_1 = D2D3_0 + D2D3_r1;
D2B1_1 = D2B1_0 + D2B1_r1;
D2B2_1 = D2B2_0 + D2B2_r1;

D3A1_1 = D3A1_0 + D3A1_r1;
D3A2_1 = D3A2_0 + D3A2_r1;
D3D1_1 = D3D1_0 + D3D1_r1;
D3D2_1 = D3D2_0 + D3D2_r1;
D3D3_1 = D3D3_0 + D3D3_r1;
D3B1_1 = D3B1_0 + D3B1_r1;
D3B2_1 = D3B2_0 + D3B2_r1;

B1A1_1 = B1A1_0 + B1A1_r1;
B1A2_1 = B1A2_0 + B1A2_r1;
B1D1_1 = B1D1_0 + B1D1_r1;
B1D2_1 = B1D2_0 + B1D2_r1;
B1D3_1 = B1D3_0 + B1D3_r1;
B1B1_1 = B1B1_0 + B1B1_r1;
B1B2_1 = B1B2_0 + B1B2_r1;

B2A1_1 = B2A1_0 + B2A1_r1;
B2A2_1 = B2A2_0 + B2A2_r1;
B2D1_1 = B2D1_0 + B2D1_r1;
B2D2_1 = B2D2_0 + B2D2_r1;
B2D3_1 = B2D3_0 + B2D3_r1;
```

```
 B2B1_1 = B2B1_0 + B2B1_r1;
 B2B2_1 = B2B2_0 + B2B2_r1;

! Fixed effects Dummy 2 (Anxiety);
NEW(A1A1_2  A1A2_2
  A1D1_2  A1D2_2  A1D3_2  A1B1_2
  A1B2_2  A2A1_2  A2A2_2
  A2D1_2  A2D2_2  A2D3_2  A2B1_2
  A2B2_2  D1A1_2  D1A2_2
  D1D1_2  D1D2_2  D1D3_2  D1B1_2
  D1B2_2  D2A1_2  D2A2_2
  D2D1_2  D2D2_2  D2D3_2  D2B1_2
  D2B2_2  D3A1_2  D3A2_2
  D3D1_2  D3D2_2  D3D3_2  D3B1_2
  D3B2_2  B1A1_2  B1A2_2
  B1D1_2  B1D2_2  B1D3_2  B1B1_2
  B1B2_2  B2A1_2  B2A2_2
  B2D1_2  B2D2_2  B2D3_2  B2B1_2
  B2B2_2);


 A1A1_2 = A1A1_0 + A1A1_r2;
 A1A2_2 = A1A2_0 + A1A2_r2;
 A1D1_2 = A1D1_0 + A1D1_r2;
 A1D2_2 = A1D2_0 + A1D2_r2;
 A1D3_2 = A1D3_0 + A1D3_r2;
 A1B1_2 = A1B1_0 + A1B1_r2;
 A1B2_2 = A1B2_0 + A1B2_r2;

 A2A1_2 = A2A1_0 + A2A1_r2;
 A2A2_2 = A2A2_0 + A2A2_r2;
 A2D1_2 = A2D1_0 + A2D1_r2;
 A2D2_2 = A2D2_0 + A2D2_r2;
 A2D3_2 = A2D3_0 + A2D3_r2;
 A2B1_2 = A2B1_0 + A2B1_r2;
 A2B2_2 = A2B2_0 + A2B2_r2;

 D1A1_2 = D1A1_0 + D1A1_r2;
 D1A2_2 = D1A2_0 + D1A2_r2;
 D1D1_2 = D1D1_0 + D1D1_r2;
 D1D2_2 = D1D2_0 + D1D2_r2;
 D1D3_2 = D1D3_0 + D1D3_r2;
 D1B1_2 = D1B1_0 + D1B1_r2;
 D1B2_2 = D1B2_0 + D1B2_r2;

 D2A1_2 = D2A1_0 + D2A1_r2;
 D2A2_2 = D2A2_0 + D2A2_r2;
 D2D1_2 = D2D1_0 + D2D1_r2;
 D2D2_2 = D2D2_0 + D2D2_r2;
```

```
D2D3_2 = D2D3_0 + D2D3_r2;
D2B1_2 = D2B1_0 + D2B1_r2;
D2B2_2 = D2B2_0 + D2B2_r2;

D3A1_2 = D3A1_0 + D3A1_r2;
D3A2_2 = D3A2_0 + D3A2_r2;
D3D1_2 = D3D1_0 + D3D1_r2;
D3D2_2 = D3D2_0 + D3D2_r2;
D3D3_2 = D3D3_0 + D3D3_r2;
D3B1_2 = D3B1_0 + D3B1_r2;
D3B2_2 = D3B2_0 + D3B2_r2;

B1A1_2 = B1A1_0 + B1A1_r2;
B1A2_2 = B1A2_0 + B1A2_r2;
B1D1_2 = B1D1_0 + B1D1_r2;
B1D2_2 = B1D2_0 + B1D2_r2;
B1D3_2 = B1D3_0 + B1D3_r2;
B1B1_2 = B1B1_0 + B1B1_r2;
B1B2_2 = B1B2_0 + B1B2_r2;

B2A1_2 = B2A1_0 + B2A1_r2;
B2A2_2 = B2A2_0 + B2A2_r2;
B2D1_2 = B2D1_0 + B2D1_r2;
B2D2_2 = B2D2_0 + B2D2_r2;
B2D3_2 = B2D3_0 + B2D3_r2;
B2B1_2 = B2B1_0 + B2B1_r2;
B2B2_2 = B2B2_0 + B2B2_r2;


! Define every indirect effect per group
! indirect effects A1 group 0
NEW(A1A1A1_0
A2A1A1_0
D1A1A1_0
D2A1A1_0
D3A1A1_0
B1A1A1_0
B2A1A1_0
A1A1A2_0
A2A1A2_0
D1A1A2_0
D2A1A2_0
D3A1A2_0
B1A1A2_0
B2A1A2_0
A1A1D1_0
A2A1D1_0
D1A1D1_0
D2A1D1_0
```

```
 D3A1D1_0
 B1A1D1_0
 B2A1D1_0
 A1A1D2_0
 A2A1D2_0
 D1A1D2_0
 D2A1D2_0
 D3A1D2_0
 B1A1D2_0
 B2A1D2_0
 A1A1D3_0
 A2A1D3_0
 D1A1D3_0
 D2A1D3_0
 D3A1D3_0
 B1A1D3_0
 B2A1D3_0
 A1A1B1_0
 A2A1B1_0
 D1A1B1_0
 D2A1B1_0
 D3A1B1_0
 B1A1B1_0
 B2A1B1_0
 A1A1B2_0
 A2A1B2_0
 D1A1B2_0
 D2A1B2_0
 D3A1B2_0
 B1A1B2_0
 B2A1B2_0);
A1A1A1_0 = ((A1A1_0)*(A1A1_0))*1000 ;
 A2A1A1_0 = ((A2A1_0)*(A1A1_0))*1000 ;
 D1A1A1_0 = ((D1A1_0)*(A1A1_0))*1000 ;
 D2A1A1_0 = ((D2A1_0)*(A1A1_0))*1000 ;
 D3A1A1_0 = ((D3A1_0)*(A1A1_0))*1000 ;
 B1A1A1_0 = ((B1A1_0)*(A1A1_0))*1000 ;
 B2A1A1_0 = ((B2A1_0)*(A1A1_0))*1000 ;
 A1A1A2_0 = ((A1A1_0)*(A1A2_0))*1000 ;
 A2A1A2_0 = ((A2A1_0)*(A1A2_0))*1000 ;
 D1A1A2_0 = ((D1A1_0)*(A1A2_0))*1000 ;
 D2A1A2_0 = ((D2A1_0)*(A1A2_0))*1000 ;
 D3A1A2_0 = ((D3A1_0)*(A1A2_0))*1000 ;
 B1A1A2_0 = ((B1A1_0)*(A1A2_0))*1000 ;
 B2A1A2_0 = ((B2A1_0)*(A1A2_0))*1000 ;
 A1A1D1_0 = ((A1A1_0)*(A1D1_0))*1000 ;
 A2A1D1_0 = ((A2A1_0)*(A1D1_0))*1000 ;
 D1A1D1_0 = ((D1A1_0)*(A1D1_0))*1000 ;
 D2A1D1_0 = ((D2A1_0)*(A1D1_0))*1000 ;
```

```
D3A1D1_0 = ((D3A1_0)*(A1D1_0))*1000 ;
B1A1D1_0 = ((B1A1_0)*(A1D1_0))*1000 ;
B2A1D1_0 = ((B2A1_0)*(A1D1_0))*1000 ;
A1A1D2_0 = ((A1A1_0)*(A1D2_0))*1000 ;
A2A1D2_0 = ((A2A1_0)*(A1D2_0))*1000 ;
D1A1D2_0 = ((D1A1_0)*(A1D2_0))*1000 ;
D2A1D2_0 = ((D2A1_0)*(A1D2_0))*1000 ;
D3A1D2_0 = ((D3A1_0)*(A1D2_0))*1000 ;
B1A1D2_0 = ((B1A1_0)*(A1D2_0))*1000 ;
B2A1D2_0 = ((B2A1_0)*(A1D2_0))*1000 ;
A1A1D3_0 = ((A1A1_0)*(A1D3_0))*1000 ;
A2A1D3_0 = ((A2A1_0)*(A1D3_0))*1000 ;
D1A1D3_0 = ((D1A1_0)*(A1D3_0))*1000 ;
D2A1D3_0 = ((D2A1_0)*(A1D3_0))*1000 ;
D3A1D3_0 = ((D3A1_0)*(A1D3_0))*1000 ;
B1A1D3_0 = ((B1A1_0)*(A1D3_0))*1000 ;
B2A1D3_0 = ((B2A1_0)*(A1D3_0))*1000 ;
A1A1B1_0 = ((A1A1_0)*(A1B1_0))*1000 ;
A2A1B1_0 = ((A2A1_0)*(A1B1_0))*1000 ;
D1A1B1_0 = ((D1A1_0)*(A1B1_0))*1000 ;
D2A1B1_0 = ((D2A1_0)*(A1B1_0))*1000 ;
D3A1B1_0 = ((D3A1_0)*(A1B1_0))*1000 ;
B1A1B1_0 = ((B1A1_0)*(A1B1_0))*1000 ;
B2A1B1_0 = ((B2A1_0)*(A1B1_0))*1000 ;
A1A1B2_0 = ((A1A1_0)*(A1B2_0))*1000 ;
A2A1B2_0 = ((A2A1_0)*(A1B2_0))*1000 ;
D1A1B2_0 = ((D1A1_0)*(A1B2_0))*1000 ;
D2A1B2_0 = ((D2A1_0)*(A1B2_0))*1000 ;
D3A1B2_0 = ((D3A1_0)*(A1B2_0))*1000 ;
B1A1B2_0 = ((B1A1_0)*(A1B2_0))*1000 ;
B2A1B2_0 = ((B2A1_0)*(A1B2_0))*1000 ;

! indirect effects A1 group 1
NEW(A1A1A1_1
A2A1A1_1
D1A1A1_1
D2A1A1_1
D3A1A1_1
B1A1A1_1
B2A1A1_1
A1A1A2_1
A2A1A2_1
D1A1A2_1
D2A1A2_1
D3A1A2_1
B1A1A2_1
B2A1A2_1
A1A1D1_1
A2A1D1_1
```

```
 D1A1D1_1
 D2A1D1_1
 D3A1D1_1
 B1A1D1_1
 B2A1D1_1
 A1A1D2_1
 A2A1D2_1
 D1A1D2_1
 D2A1D2_1
 D3A1D2_1
 B1A1D2_1
 B2A1D2_1
 A1A1D3_1
 A2A1D3_1
 D1A1D3_1
 D2A1D3_1
 D3A1D3_1
 B1A1D3_1
 B2A1D3_1
 A1A1B1_1
 A2A1B1_1
 D1A1B1_1
 D2A1B1_1
 D3A1B1_1
 B1A1B1_1
 B2A1B1_1
 A1A1B2_1
 A2A1B2_1
 D1A1B2_1
 D2A1B2_1
 D3A1B2_1
 B1A1B2_1
 B2A1B2_1);
A1A1A1_1 = ((A1A1_1)*(A1A1_1))*1000 ;
 A2A1A1_1 = ((A2A1_1)*(A1A1_1))*1000 ;
 D1A1A1_1 = ((D1A1_1)*(A1A1_1))*1000 ;
 D2A1A1_1 = ((D2A1_1)*(A1A1_1))*1000 ;
 D3A1A1_1 = ((D3A1_1)*(A1A1_1))*1000 ;
 B1A1A1_1 = ((B1A1_1)*(A1A1_1))*1000 ;
 B2A1A1_1 = ((B2A1_1)*(A1A1_1))*1000 ;
 A1A1A2_1 = ((A1A1_1)*(A1A2_1))*1000 ;
 A2A1A2_1 = ((A2A1_1)*(A1A2_1))*1000 ;
 D1A1A2_1 = ((D1A1_1)*(A1A2_1))*1000 ;
 D2A1A2_1 = ((D2A1_1)*(A1A2_1))*1000 ;
 D3A1A2_1 = ((D3A1_1)*(A1A2_1))*1000 ;
 B1A1A2_1 = ((B1A1_1)*(A1A2_1))*1000 ;
 B2A1A2_1 = ((B2A1_1)*(A1A2_1))*1000 ;
 A1A1D1_1 = ((A1A1_1)*(A1D1_1))*1000 ;
 A2A1D1_1 = ((A2A1_1)*(A1D1_1))*1000 ;
```

```
D1A1D1_1 = ((D1A1_1)*(A1D1_1))*1000 ;
D2A1D1_1 = ((D2A1_1)*(A1D1_1))*1000 ;
D3A1D1_1 = ((D3A1_1)*(A1D1_1))*1000 ;
B1A1D1_1 = ((B1A1_1)*(A1D1_1))*1000 ;
B2A1D1_1 = ((B2A1_1)*(A1D1_1))*1000 ;
A1A1D2_1 = ((A1A1_1)*(A1D2_1))*1000 ;
A2A1D2_1 = ((A2A1_1)*(A1D2_1))*1000 ;
D1A1D2_1 = ((D1A1_1)*(A1D2_1))*1000 ;
D2A1D2_1 = ((D2A1_1)*(A1D2_1))*1000 ;
D3A1D2_1 = ((D3A1_1)*(A1D2_1))*1000 ;
B1A1D2_1 = ((B1A1_1)*(A1D2_1))*1000 ;
B2A1D2_1 = ((B2A1_1)*(A1D2_1))*1000 ;
A1A1D3_1 = ((A1A1_1)*(A1D3_1))*1000 ;
A2A1D3_1 = ((A2A1_1)*(A1D3_1))*1000 ;
D1A1D3_1 = ((D1A1_1)*(A1D3_1))*1000 ;
D2A1D3_1 = ((D2A1_1)*(A1D3_1))*1000 ;
D3A1D3_1 = ((D3A1_1)*(A1D3_1))*1000 ;
B1A1D3_1 = ((B1A1_1)*(A1D3_1))*1000 ;
B2A1D3_1 = ((B2A1_1)*(A1D3_1))*1000 ;
A1A1B1_1 = ((A1A1_1)*(A1B1_1))*1000 ;
A2A1B1_1 = ((A2A1_1)*(A1B1_1))*1000 ;
D1A1B1_1 = ((D1A1_1)*(A1B1_1))*1000 ;
D2A1B1_1 = ((D2A1_1)*(A1B1_1))*1000 ;
D3A1B1_1 = ((D3A1_1)*(A1B1_1))*1000 ;
B1A1B1_1 = ((B1A1_1)*(A1B1_1))*1000 ;
B2A1B1_1 = ((B2A1_1)*(A1B1_1))*1000 ;
A1A1B2_1 = ((A1A1_1)*(A1B2_1))*1000 ;
A2A1B2_1 = ((A2A1_1)*(A1B2_1))*1000 ;
D1A1B2_1 = ((D1A1_1)*(A1B2_1))*1000 ;
D2A1B2_1 = ((D2A1_1)*(A1B2_1))*1000 ;
D3A1B2_1 = ((D3A1_1)*(A1B2_1))*1000 ;
B1A1B2_1 = ((B1A1_1)*(A1B2_1))*1000 ;
B2A1B2_1 = ((B2A1_1)*(A1B2_1))*1000 ;

! indirect effects A1 group 2
NEW(A1A1A1_2
A2A1A1_2
D1A1A1_2
D2A1A1_2
D3A1A1_2
B1A1A1_2
B2A1A1_2
A1A1A2_2
A2A1A2_2
D1A1A2_2
D2A1A2_2
D3A1A2_2
B1A1A2_2
B2A1A2_2
```

```
 A1A1D1_2
 A2A1D1_2
 D1A1D1_2
 D2A1D1_2
 D3A1D1_2
 B1A1D1_2
 B2A1D1_2
 A1A1D2_2
 A2A1D2_2
 D1A1D2_2
 D2A1D2_2
 D3A1D2_2
 B1A1D2_2
 B2A1D2_2
 A1A1D3_2
 A2A1D3_2
 D1A1D3_2
 D2A1D3_2
 D3A1D3_2
 B1A1D3_2
 B2A1D3_2
 A1A1B1_2
 A2A1B1_2
 D1A1B1_2
 D2A1B1_2
 D3A1B1_2
 B1A1B1_2
 B2A1B1_2
 A1A1B2_2
 A2A1B2_2
 D1A1B2_2
 D2A1B2_2
 D3A1B2_2
 B1A1B2_2
 B2A1B2_2);
A1A1A1_2 = ((A1A1_2)*(A1A1_2))*1000 ;
 A2A1A1_2 = ((A2A1_2)*(A1A1_2))*1000 ;
 D1A1A1_2 = ((D1A1_2)*(A1A1_2))*1000 ;
 D2A1A1_2 = ((D2A1_2)*(A1A1_2))*1000 ;
 D3A1A1_2 = ((D3A1_2)*(A1A1_2))*1000 ;
 B1A1A1_2 = ((B1A1_2)*(A1A1_2))*1000 ;
 B2A1A1_2 = ((B2A1_2)*(A1A1_2))*1000 ;
 A1A1A2_2 = ((A1A1_2)*(A1A2_2))*1000 ;
 A2A1A2_2 = ((A2A1_2)*(A1A2_2))*1000 ;
 D1A1A2_2 = ((D1A1_2)*(A1A2_2))*1000 ;
 D2A1A2_2 = ((D2A1_2)*(A1A2_2))*1000 ;
 D3A1A2_2 = ((D3A1_2)*(A1A2_2))*1000 ;
 B1A1A2_2 = ((B1A1_2)*(A1A2_2))*1000 ;
 B2A1A2_2 = ((B2A1_2)*(A1A2_2))*1000 ;
```

```
A1A1D1_2 = ((A1A1_2)*(A1D1_2))*1000 ;
A2A1D1_2 = ((A2A1_2)*(A1D1_2))*1000 ;
D1A1D1_2 = ((D1A1_2)*(A1D1_2))*1000 ;
D2A1D1_2 = ((D2A1_2)*(A1D1_2))*1000 ;
D3A1D1_2 = ((D3A1_2)*(A1D1_2))*1000 ;
B1A1D1_2 = ((B1A1_2)*(A1D1_2))*1000 ;
B2A1D1_2 = ((B2A1_2)*(A1D1_2))*1000 ;
A1A1D2_2 = ((A1A1_2)*(A1D2_2))*1000 ;
A2A1D2_2 = ((A2A1_2)*(A1D2_2))*1000 ;
D1A1D2_2 = ((D1A1_2)*(A1D2_2))*1000 ;
D2A1D2_2 = ((D2A1_2)*(A1D2_2))*1000 ;
D3A1D2_2 = ((D3A1_2)*(A1D2_2))*1000 ;
B1A1D2_2 = ((B1A1_2)*(A1D2_2))*1000 ;
B2A1D2_2 = ((B2A1_2)*(A1D2_2))*1000 ;
A1A1D3_2 = ((A1A1_2)*(A1D3_2))*1000 ;
A2A1D3_2 = ((A2A1_2)*(A1D3_2))*1000 ;
D1A1D3_2 = ((D1A1_2)*(A1D3_2))*1000 ;
D2A1D3_2 = ((D2A1_2)*(A1D3_2))*1000 ;
D3A1D3_2 = ((D3A1_2)*(A1D3_2))*1000 ;
B1A1D3_2 = ((B1A1_2)*(A1D3_2))*1000 ;
B2A1D3_2 = ((B2A1_2)*(A1D3_2))*1000 ;
A1A1B1_2 = ((A1A1_2)*(A1B1_2))*1000 ;
A2A1B1_2 = ((A2A1_2)*(A1B1_2))*1000 ;
D1A1B1_2 = ((D1A1_2)*(A1B1_2))*1000 ;
D2A1B1_2 = ((D2A1_2)*(A1B1_2))*1000 ;
D3A1B1_2 = ((D3A1_2)*(A1B1_2))*1000 ;
B1A1B1_2 = ((B1A1_2)*(A1B1_2))*1000 ;
B2A1B1_2 = ((B2A1_2)*(A1B1_2))*1000 ;
A1A1B2_2 = ((A1A1_2)*(A1B2_2))*1000 ;
A2A1B2_2 = ((A2A1_2)*(A1B2_2))*1000 ;
D1A1B2_2 = ((D1A1_2)*(A1B2_2))*1000 ;
D2A1B2_2 = ((D2A1_2)*(A1B2_2))*1000 ;
D3A1B2_2 = ((D3A1_2)*(A1B2_2))*1000 ;
B1A1B2_2 = ((B1A1_2)*(A1B2_2))*1000 ;
B2A1B2_2 = ((B2A1_2)*(A1B2_2))*1000 ;

! indirect effects A2 group 0
NEW(A1A2A1_0
A2A2A1_0
D1A2A1_0
D2A2A1_0
D3A2A1_0
B1A2A1_0
B2A2A1_0
A1A2A2_0
A2A2A2_0
D1A2A2_0
D2A2A2_0
D3A2A2_0
```

```
 B1A2A2_0
 B2A2A2_0
 A1A2D1_0
 A2A2D1_0
 D1A2D1_0
 D2A2D1_0
 D3A2D1_0
 B1A2D1_0
 B2A2D1_0
 A1A2D2_0
 A2A2D2_0
 D1A2D2_0
 D2A2D2_0
 D3A2D2_0
 B1A2D2_0
 B2A2D2_0
 A1A2D3_0
 A2A2D3_0
 D1A2D3_0
 D2A2D3_0
 D3A2D3_0
 B1A2D3_0
 B2A2D3_0
 A1A2B1_0
 A2A2B1_0
 D1A2B1_0
 D2A2B1_0
 D3A2B1_0
 B1A2B1_0
 B2A2B1_0
 A1A2B2_0
 A2A2B2_0
 D1A2B2_0
 D2A2B2_0
 D3A2B2_0
 B1A2B2_0
 B2A2B2_0);
A1A2A1_0 = ((A1A2_0)*(A2A1_0))*1000 ;
 A2A2A1_0 = ((A2A2_0)*(A2A1_0))*1000 ;
 D1A2A1_0 = ((D1A2_0)*(A2A1_0))*1000 ;
 D2A2A1_0 = ((D2A2_0)*(A2A1_0))*1000 ;
 D3A2A1_0 = ((D3A2_0)*(A2A1_0))*1000 ;
 B1A2A1_0 = ((B1A2_0)*(A2A1_0))*1000 ;
 B2A2A1_0 = ((B2A2_0)*(A2A1_0))*1000 ;
 A1A2A2_0 = ((A1A2_0)*(A2A2_0))*1000 ;
 A2A2A2_0 = ((A2A2_0)*(A2A2_0))*1000 ;
 D1A2A2_0 = ((D1A2_0)*(A2A2_0))*1000 ;
 D2A2A2_0 = ((D2A2_0)*(A2A2_0))*1000 ;
 D3A2A2_0 = ((D3A2_0)*(A2A2_0))*1000 ;
```

```
B1A2A2_0 = ((B1A2_0)*(A2A2_0))*1000 ;
B2A2A2_0 = ((B2A2_0)*(A2A2_0))*1000 ;
A1A2D1_0 = ((A1A2_0)*(A2D1_0))*1000 ;
A2A2D1_0 = ((A2A2_0)*(A2D1_0))*1000 ;
D1A2D1_0 = ((D1A2_0)*(A2D1_0))*1000 ;
D2A2D1_0 = ((D2A2_0)*(A2D1_0))*1000 ;
D3A2D1_0 = ((D3A2_0)*(A2D1_0))*1000 ;
B1A2D1_0 = ((B1A2_0)*(A2D1_0))*1000 ;
B2A2D1_0 = ((B2A2_0)*(A2D1_0))*1000 ;
A1A2D2_0 = ((A1A2_0)*(A2D2_0))*1000 ;
A2A2D2_0 = ((A2A2_0)*(A2D2_0))*1000 ;
D1A2D2_0 = ((D1A2_0)*(A2D2_0))*1000 ;
D2A2D2_0 = ((D2A2_0)*(A2D2_0))*1000 ;
D3A2D2_0 = ((D3A2_0)*(A2D2_0))*1000 ;
B1A2D2_0 = ((B1A2_0)*(A2D2_0))*1000 ;
B2A2D2_0 = ((B2A2_0)*(A2D2_0))*1000 ;
A1A2D3_0 = ((A1A2_0)*(A2D3_0))*1000 ;
A2A2D3_0 = ((A2A2_0)*(A2D3_0))*1000 ;
D1A2D3_0 = ((D1A2_0)*(A2D3_0))*1000 ;
D2A2D3_0 = ((D2A2_0)*(A2D3_0))*1000 ;
D3A2D3_0 = ((D3A2_0)*(A2D3_0))*1000 ;
B1A2D3_0 = ((B1A2_0)*(A2D3_0))*1000 ;
B2A2D3_0 = ((B2A2_0)*(A2D3_0))*1000 ;
A1A2B1_0 = ((A1A2_0)*(A2B1_0))*1000 ;
A2A2B1_0 = ((A2A2_0)*(A2B1_0))*1000 ;
D1A2B1_0 = ((D1A2_0)*(A2B1_0))*1000 ;
D2A2B1_0 = ((D2A2_0)*(A2B1_0))*1000 ;
D3A2B1_0 = ((D3A2_0)*(A2B1_0))*1000 ;
B1A2B1_0 = ((B1A2_0)*(A2B1_0))*1000 ;
B2A2B1_0 = ((B2A2_0)*(A2B1_0))*1000 ;
A1A2B2_0 = ((A1A2_0)*(A2B2_0))*1000 ;
A2A2B2_0 = ((A2A2_0)*(A2B2_0))*1000 ;
D1A2B2_0 = ((D1A2_0)*(A2B2_0))*1000 ;
D2A2B2_0 = ((D2A2_0)*(A2B2_0))*1000 ;
D3A2B2_0 = ((D3A2_0)*(A2B2_0))*1000 ;
B1A2B2_0 = ((B1A2_0)*(A2B2_0))*1000 ;
B2A2B2_0 = ((B2A2_0)*(A2B2_0))*1000 ;

! indirect effects A2 group 1
NEW(A1A2A1_1
A2A2A1_1
D1A2A1_1
D2A2A1_1
D3A2A1_1
B1A2A1_1
B2A2A1_1
A1A2A2_1
A2A2A2_1
D1A2A2_1
```

```
 D2A2A2_1
 D3A2A2_1
 B1A2A2_1
 B2A2A2_1
 A1A2D1_1
 A2A2D1_1
 D1A2D1_1
 D2A2D1_1
 D3A2D1_1
 B1A2D1_1
 B2A2D1_1
 A1A2D2_1
 A2A2D2_1
 D1A2D2_1
 D2A2D2_1
 D3A2D2_1
 B1A2D2_1
 B2A2D2_1
 A1A2D3_1
 A2A2D3_1
 D1A2D3_1
 D2A2D3_1
 D3A2D3_1
 B1A2D3_1
 B2A2D3_1
 A1A2B1_1
 A2A2B1_1
 D1A2B1_1
 D2A2B1_1
 D3A2B1_1
 B1A2B1_1
 B2A2B1_1
 A1A2B2_1
 A2A2B2_1
 D1A2B2_1
 D2A2B2_1
 D3A2B2_1
 B1A2B2_1
 B2A2B2_1);
A1A2A1_1 = ((A1A2_1)*(A2A1_1))*1000 ;
 A2A2A1_1 = ((A2A2_1)*(A2A1_1))*1000 ;
 D1A2A1_1 = ((D1A2_1)*(A2A1_1))*1000 ;
 D2A2A1_1 = ((D2A2_1)*(A2A1_1))*1000 ;
 D3A2A1_1 = ((D3A2_1)*(A2A1_1))*1000 ;
 B1A2A1_1 = ((B1A2_1)*(A2A1_1))*1000 ;
 B2A2A1_1 = ((B2A2_1)*(A2A1_1))*1000 ;
 A1A2A2_1 = ((A1A2_1)*(A2A2_1))*1000 ;
 A2A2A2_1 = ((A2A2_1)*(A2A2_1))*1000 ;
 D1A2A2_1 = ((D1A2_1)*(A2A2_1))*1000 ;
```

```
D2A2A2_1 = ((D2A2_1)*(A2A2_1))*1000 ;
D3A2A2_1 = ((D3A2_1)*(A2A2_1))*1000 ;
B1A2A2_1 = ((B1A2_1)*(A2A2_1))*1000 ;
B2A2A2_1 = ((B2A2_1)*(A2A2_1))*1000 ;
A1A2D1_1 = ((A1A2_1)*(A2D1_1))*1000 ;
A2A2D1_1 = ((A2A2_1)*(A2D1_1))*1000 ;
D1A2D1_1 = ((D1A2_1)*(A2D1_1))*1000 ;
D2A2D1_1 = ((D2A2_1)*(A2D1_1))*1000 ;
D3A2D1_1 = ((D3A2_1)*(A2D1_1))*1000 ;
B1A2D1_1 = ((B1A2_1)*(A2D1_1))*1000 ;
B2A2D1_1 = ((B2A2_1)*(A2D1_1))*1000 ;
A1A2D2_1 = ((A1A2_1)*(A2D2_1))*1000 ;
A2A2D2_1 = ((A2A2_1)*(A2D2_1))*1000 ;
D1A2D2_1 = ((D1A2_1)*(A2D2_1))*1000 ;
D2A2D2_1 = ((D2A2_1)*(A2D2_1))*1000 ;
D3A2D2_1 = ((D3A2_1)*(A2D2_1))*1000 ;
B1A2D2_1 = ((B1A2_1)*(A2D2_1))*1000 ;
B2A2D2_1 = ((B2A2_1)*(A2D2_1))*1000 ;
A1A2D3_1 = ((A1A2_1)*(A2D3_1))*1000 ;
A2A2D3_1 = ((A2A2_1)*(A2D3_1))*1000 ;
D1A2D3_1 = ((D1A2_1)*(A2D3_1))*1000 ;
D2A2D3_1 = ((D2A2_1)*(A2D3_1))*1000 ;
D3A2D3_1 = ((D3A2_1)*(A2D3_1))*1000 ;
B1A2D3_1 = ((B1A2_1)*(A2D3_1))*1000 ;
B2A2D3_1 = ((B2A2_1)*(A2D3_1))*1000 ;
A1A2B1_1 = ((A1A2_1)*(A2B1_1))*1000 ;
A2A2B1_1 = ((A2A2_1)*(A2B1_1))*1000 ;
D1A2B1_1 = ((D1A2_1)*(A2B1_1))*1000 ;
D2A2B1_1 = ((D2A2_1)*(A2B1_1))*1000 ;
D3A2B1_1 = ((D3A2_1)*(A2B1_1))*1000 ;
B1A2B1_1 = ((B1A2_1)*(A2B1_1))*1000 ;
B2A2B1_1 = ((B2A2_1)*(A2B1_1))*1000 ;
A1A2B2_1 = ((A1A2_1)*(A2B2_1))*1000 ;
A2A2B2_1 = ((A2A2_1)*(A2B2_1))*1000 ;
D1A2B2_1 = ((D1A2_1)*(A2B2_1))*1000 ;
D2A2B2_1 = ((D2A2_1)*(A2B2_1))*1000 ;
D3A2B2_1 = ((D3A2_1)*(A2B2_1))*1000 ;
B1A2B2_1 = ((B1A2_1)*(A2B2_1))*1000 ;
B2A2B2_1 = ((B2A2_1)*(A2B2_1))*1000 ;

! indirect effects A2 group 2
NEW(A1A2A1_2
A2A2A1_2
D1A2A1_2
D2A2A1_2
D3A2A1_2
B1A2A1_2
B2A2A1_2
A1A2A2_2
```

```
 A2A2A2_2
 D1A2A2_2
 D2A2A2_2
 D3A2A2_2
 B1A2A2_2
 B2A2A2_2
 A1A2D1_2
 A2A2D1_2
 D1A2D1_2
 D2A2D1_2
 D3A2D1_2
 B1A2D1_2
 B2A2D1_2
 A1A2D2_2
 A2A2D2_2
 D1A2D2_2
 D2A2D2_2
 D3A2D2_2
 B1A2D2_2
 B2A2D2_2
 A1A2D3_2
 A2A2D3_2
 D1A2D3_2
 D2A2D3_2
 D3A2D3_2
 B1A2D3_2
 B2A2D3_2
 A1A2B1_2
 A2A2B1_2
 D1A2B1_2
 D2A2B1_2
 D3A2B1_2
 B1A2B1_2
 B2A2B1_2
 A1A2B2_2
 A2A2B2_2
 D1A2B2_2
 D2A2B2_2
 D3A2B2_2
 B1A2B2_2
 B2A2B2_2);
A1A2A1_2 = ((A1A2_2)*(A2A1_2))*1000 ;
 A2A2A1_2 = ((A2A2_2)*(A2A1_2))*1000 ;
 D1A2A1_2 = ((D1A2_2)*(A2A1_2))*1000 ;
 D2A2A1_2 = ((D2A2_2)*(A2A1_2))*1000 ;
 D3A2A1_2 = ((D3A2_2)*(A2A1_2))*1000 ;
 B1A2A1_2 = ((B1A2_2)*(A2A1_2))*1000 ;
 B2A2A1_2 = ((B2A2_2)*(A2A1_2))*1000 ;
 A1A2A2_2 = ((A1A2_2)*(A2A2_2))*1000 ;
```

```
A2A2A2_2 = ((A2A2_2)*(A2A2_2))*1000 ;
D1A2A2_2 = ((D1A2_2)*(A2A2_2))*1000 ;
D2A2A2_2 = ((D2A2_2)*(A2A2_2))*1000 ;
D3A2A2_2 = ((D3A2_2)*(A2A2_2))*1000 ;
B1A2A2_2 = ((B1A2_2)*(A2A2_2))*1000 ;
B2A2A2_2 = ((B2A2_2)*(A2A2_2))*1000 ;
A1A2D1_2 = ((A1A2_2)*(A2D1_2))*1000 ;
A2A2D1_2 = ((A2A2_2)*(A2D1_2))*1000 ;
D1A2D1_2 = ((D1A2_2)*(A2D1_2))*1000 ;
D2A2D1_2 = ((D2A2_2)*(A2D1_2))*1000 ;
D3A2D1_2 = ((D3A2_2)*(A2D1_2))*1000 ;
B1A2D1_2 = ((B1A2_2)*(A2D1_2))*1000 ;
B2A2D1_2 = ((B2A2_2)*(A2D1_2))*1000 ;
A1A2D2_2 = ((A1A2_2)*(A2D2_2))*1000 ;
A2A2D2_2 = ((A2A2_2)*(A2D2_2))*1000 ;
D1A2D2_2 = ((D1A2_2)*(A2D2_2))*1000 ;
D2A2D2_2 = ((D2A2_2)*(A2D2_2))*1000 ;
D3A2D2_2 = ((D3A2_2)*(A2D2_2))*1000 ;
B1A2D2_2 = ((B1A2_2)*(A2D2_2))*1000 ;
B2A2D2_2 = ((B2A2_2)*(A2D2_2))*1000 ;
A1A2D3_2 = ((A1A2_2)*(A2D3_2))*1000 ;
A2A2D3_2 = ((A2A2_2)*(A2D3_2))*1000 ;
D1A2D3_2 = ((D1A2_2)*(A2D3_2))*1000 ;
D2A2D3_2 = ((D2A2_2)*(A2D3_2))*1000 ;
D3A2D3_2 = ((D3A2_2)*(A2D3_2))*1000 ;
B1A2D3_2 = ((B1A2_2)*(A2D3_2))*1000 ;
B2A2D3_2 = ((B2A2_2)*(A2D3_2))*1000 ;
A1A2B1_2 = ((A1A2_2)*(A2B1_2))*1000 ;
A2A2B1_2 = ((A2A2_2)*(A2B1_2))*1000 ;
D1A2B1_2 = ((D1A2_2)*(A2B1_2))*1000 ;
D2A2B1_2 = ((D2A2_2)*(A2B1_2))*1000 ;
D3A2B1_2 = ((D3A2_2)*(A2B1_2))*1000 ;
B1A2B1_2 = ((B1A2_2)*(A2B1_2))*1000 ;
B2A2B1_2 = ((B2A2_2)*(A2B1_2))*1000 ;
A1A2B2_2 = ((A1A2_2)*(A2B2_2))*1000 ;
A2A2B2_2 = ((A2A2_2)*(A2B2_2))*1000 ;
D1A2B2_2 = ((D1A2_2)*(A2B2_2))*1000 ;
D2A2B2_2 = ((D2A2_2)*(A2B2_2))*1000 ;
D3A2B2_2 = ((D3A2_2)*(A2B2_2))*1000 ;
B1A2B2_2 = ((B1A2_2)*(A2B2_2))*1000 ;
B2A2B2_2 = ((B2A2_2)*(A2B2_2))*1000 ;

! indirect effects D1 group 0
NEW(A1D1A1_0
A2D1A1_0
D1D1A1_0
D2D1A1_0
D3D1A1_0
B1D1A1_0
```

```
 B2D1A1_0
 A1D1A2_0
 A2D1A2_0
 D1D1A2_0
 D2D1A2_0
 D3D1A2_0
 B1D1A2_0
 B2D1A2_0
 A1D1D1_0
 A2D1D1_0
 D1D1D1_0
 D2D1D1_0
 D3D1D1_0
 B1D1D1_0
 B2D1D1_0
 A1D1D2_0
 A2D1D2_0
 D1D1D2_0
 D2D1D2_0
 D3D1D2_0
 B1D1D2_0
 B2D1D2_0
 A1D1D3_0
 A2D1D3_0
 D1D1D3_0
 D2D1D3_0
 D3D1D3_0
 B1D1D3_0
 B2D1D3_0
 A1D1B1_0
 A2D1B1_0
 D1D1B1_0
 D2D1B1_0
 D3D1B1_0
 B1D1B1_0
 B2D1B1_0
 A1D1B2_0
 A2D1B2_0
 D1D1B2_0
 D2D1B2_0
 D3D1B2_0
 B1D1B2_0
 B2D1B2_0);
A1D1A1_0 = ((A1D1_0)*(D1A1_0))*1000 ;
 A2D1A1_0 = ((A2D1_0)*(D1A1_0))*1000 ;
 D1D1A1_0 = ((D1D1_0)*(D1A1_0))*1000 ;
 D2D1A1_0 = ((D2D1_0)*(D1A1_0))*1000 ;
 D3D1A1_0 = ((D3D1_0)*(D1A1_0))*1000 ;
 B1D1A1_0 = ((B1D1_0)*(D1A1_0))*1000 ;
```

```
B2D1A1_0 = ((B2D1_0)*(D1A1_0))*1000 ;
A1D1A2_0 = ((A1D1_0)*(D1A2_0))*1000 ;
A2D1A2_0 = ((A2D1_0)*(D1A2_0))*1000 ;
D1D1A2_0 = ((D1D1_0)*(D1A2_0))*1000 ;
D2D1A2_0 = ((D2D1_0)*(D1A2_0))*1000 ;
D3D1A2_0 = ((D3D1_0)*(D1A2_0))*1000 ;
B1D1A2_0 = ((B1D1_0)*(D1A2_0))*1000 ;
B2D1A2_0 = ((B2D1_0)*(D1A2_0))*1000 ;
A1D1D1_0 = ((A1D1_0)*(D1D1_0))*1000 ;
A2D1D1_0 = ((A2D1_0)*(D1D1_0))*1000 ;
D1D1D1_0 = ((D1D1_0)*(D1D1_0))*1000 ;
D2D1D1_0 = ((D2D1_0)*(D1D1_0))*1000 ;
D3D1D1_0 = ((D3D1_0)*(D1D1_0))*1000 ;
B1D1D1_0 = ((B1D1_0)*(D1D1_0))*1000 ;
B2D1D1_0 = ((B2D1_0)*(D1D1_0))*1000 ;
A1D1D2_0 = ((A1D1_0)*(D1D2_0))*1000 ;
A2D1D2_0 = ((A2D1_0)*(D1D2_0))*1000 ;
D1D1D2_0 = ((D1D1_0)*(D1D2_0))*1000 ;
D2D1D2_0 = ((D2D1_0)*(D1D2_0))*1000 ;
D3D1D2_0 = ((D3D1_0)*(D1D2_0))*1000 ;
B1D1D2_0 = ((B1D1_0)*(D1D2_0))*1000 ;
B2D1D2_0 = ((B2D1_0)*(D1D2_0))*1000 ;
A1D1D3_0 = ((A1D1_0)*(D1D3_0))*1000 ;
A2D1D3_0 = ((A2D1_0)*(D1D3_0))*1000 ;
D1D1D3_0 = ((D1D1_0)*(D1D3_0))*1000 ;
D2D1D3_0 = ((D2D1_0)*(D1D3_0))*1000 ;
D3D1D3_0 = ((D3D1_0)*(D1D3_0))*1000 ;
B1D1D3_0 = ((B1D1_0)*(D1D3_0))*1000 ;
B2D1D3_0 = ((B2D1_0)*(D1D3_0))*1000 ;
A1D1B1_0 = ((A1D1_0)*(D1B1_0))*1000 ;
A2D1B1_0 = ((A2D1_0)*(D1B1_0))*1000 ;
D1D1B1_0 = ((D1D1_0)*(D1B1_0))*1000 ;
D2D1B1_0 = ((D2D1_0)*(D1B1_0))*1000 ;
D3D1B1_0 = ((D3D1_0)*(D1B1_0))*1000 ;
B1D1B1_0 = ((B1D1_0)*(D1B1_0))*1000 ;
B2D1B1_0 = ((B2D1_0)*(D1B1_0))*1000 ;
A1D1B2_0 = ((A1D1_0)*(D1B2_0))*1000 ;
A2D1B2_0 = ((A2D1_0)*(D1B2_0))*1000 ;
D1D1B2_0 = ((D1D1_0)*(D1B2_0))*1000 ;
D2D1B2_0 = ((D2D1_0)*(D1B2_0))*1000 ;
D3D1B2_0 = ((D3D1_0)*(D1B2_0))*1000 ;
B1D1B2_0 = ((B1D1_0)*(D1B2_0))*1000 ;
B2D1B2_0 = ((B2D1_0)*(D1B2_0))*1000 ;

! indirect effects D1 group 1
NEW(A1D1A1_1
A2D1A1_1
D1D1A1_1
D2D1A1_1
```

```
 D3D1A1_1
 B1D1A1_1
 B2D1A1_1
 A1D1A2_1
 A2D1A2_1
 D1D1A2_1
 D2D1A2_1
 D3D1A2_1
 B1D1A2_1
 B2D1A2_1
 A1D1D1_1
 A2D1D1_1
 D1D1D1_1
 D2D1D1_1
 D3D1D1_1
 B1D1D1_1
 B2D1D1_1
 A1D1D2_1
 A2D1D2_1
 D1D1D2_1
 D2D1D2_1
 D3D1D2_1
 B1D1D2_1
 B2D1D2_1
 A1D1D3_1
 A2D1D3_1
 D1D1D3_1
 D2D1D3_1
 D3D1D3_1
 B1D1D3_1
 B2D1D3_1
 A1D1B1_1
 A2D1B1_1
 D1D1B1_1
 D2D1B1_1
 D3D1B1_1
 B1D1B1_1
 B2D1B1_1
 A1D1B2_1
 A2D1B2_1
 D1D1B2_1
 D2D1B2_1
 D3D1B2_1
 B1D1B2_1
 B2D1B2_1);
A1D1A1_1 = ((A1D1_1)*(D1A1_1))*1000 ;
 A2D1A1_1 = ((A2D1_1)*(D1A1_1))*1000 ;
 D1D1A1_1 = ((D1D1_1)*(D1A1_1))*1000 ;
 D2D1A1_1 = ((D2D1_1)*(D1A1_1))*1000 ;
```

```
D3D1A1_1 = ((D3D1_1)*(D1A1_1))*1000 ;
B1D1A1_1 = ((B1D1_1)*(D1A1_1))*1000 ;
B2D1A1_1 = ((B2D1_1)*(D1A1_1))*1000 ;
A1D1A2_1 = ((A1D1_1)*(D1A2_1))*1000 ;
A2D1A2_1 = ((A2D1_1)*(D1A2_1))*1000 ;
D1D1A2_1 = ((D1D1_1)*(D1A2_1))*1000 ;
D2D1A2_1 = ((D2D1_1)*(D1A2_1))*1000 ;
D3D1A2_1 = ((D3D1_1)*(D1A2_1))*1000 ;
B1D1A2_1 = ((B1D1_1)*(D1A2_1))*1000 ;
B2D1A2_1 = ((B2D1_1)*(D1A2_1))*1000 ;
A1D1D1_1 = ((A1D1_1)*(D1D1_1))*1000 ;
A2D1D1_1 = ((A2D1_1)*(D1D1_1))*1000 ;
D1D1D1_1 = ((D1D1_1)*(D1D1_1))*1000 ;
D2D1D1_1 = ((D2D1_1)*(D1D1_1))*1000 ;
D3D1D1_1 = ((D3D1_1)*(D1D1_1))*1000 ;
B1D1D1_1 = ((B1D1_1)*(D1D1_1))*1000 ;
B2D1D1_1 = ((B2D1_1)*(D1D1_1))*1000 ;
A1D1D2_1 = ((A1D1_1)*(D1D2_1))*1000 ;
A2D1D2_1 = ((A2D1_1)*(D1D2_1))*1000 ;
D1D1D2_1 = ((D1D1_1)*(D1D2_1))*1000 ;
D2D1D2_1 = ((D2D1_1)*(D1D2_1))*1000 ;
D3D1D2_1 = ((D3D1_1)*(D1D2_1))*1000 ;
B1D1D2_1 = ((B1D1_1)*(D1D2_1))*1000 ;
B2D1D2_1 = ((B2D1_1)*(D1D2_1))*1000 ;
A1D1D3_1 = ((A1D1_1)*(D1D3_1))*1000 ;
A2D1D3_1 = ((A2D1_1)*(D1D3_1))*1000 ;
D1D1D3_1 = ((D1D1_1)*(D1D3_1))*1000 ;
D2D1D3_1 = ((D2D1_1)*(D1D3_1))*1000 ;
D3D1D3_1 = ((D3D1_1)*(D1D3_1))*1000 ;
B1D1D3_1 = ((B1D1_1)*(D1D3_1))*1000 ;
B2D1D3_1 = ((B2D1_1)*(D1D3_1))*1000 ;
A1D1B1_1 = ((A1D1_1)*(D1B1_1))*1000 ;
A2D1B1_1 = ((A2D1_1)*(D1B1_1))*1000 ;
D1D1B1_1 = ((D1D1_1)*(D1B1_1))*1000 ;
D2D1B1_1 = ((D2D1_1)*(D1B1_1))*1000 ;
D3D1B1_1 = ((D3D1_1)*(D1B1_1))*1000 ;
B1D1B1_1 = ((B1D1_1)*(D1B1_1))*1000 ;
B2D1B1_1 = ((B2D1_1)*(D1B1_1))*1000 ;
A1D1B2_1 = ((A1D1_1)*(D1B2_1))*1000 ;
A2D1B2_1 = ((A2D1_1)*(D1B2_1))*1000 ;
D1D1B2_1 = ((D1D1_1)*(D1B2_1))*1000 ;
D2D1B2_1 = ((D2D1_1)*(D1B2_1))*1000 ;
D3D1B2_1 = ((D3D1_1)*(D1B2_1))*1000 ;
B1D1B2_1 = ((B1D1_1)*(D1B2_1))*1000 ;
B2D1B2_1 = ((B2D1_1)*(D1B2_1))*1000 ;

! indirect effects D1 group 2
NEW(A1D1A1_2
A2D1A1_2
```

```
 D1D1A1_2
 D2D1A1_2
 D3D1A1_2
 B1D1A1_2
 B2D1A1_2
 A1D1A2_2
 A2D1A2_2
 D1D1A2_2
 D2D1A2_2
 D3D1A2_2
 B1D1A2_2
 B2D1A2_2
 A1D1D1_2
 A2D1D1_2
 D1D1D1_2
 D2D1D1_2
 D3D1D1_2
 B1D1D1_2
 B2D1D1_2
 A1D1D2_2
 A2D1D2_2
 D1D1D2_2
 D2D1D2_2
 D3D1D2_2
 B1D1D2_2
 B2D1D2_2
 A1D1D3_2
 A2D1D3_2
 D1D1D3_2
 D2D1D3_2
 D3D1D3_2
 B1D1D3_2
 B2D1D3_2
 A1D1B1_2
 A2D1B1_2
 D1D1B1_2
 D2D1B1_2
 D3D1B1_2
 B1D1B1_2
 B2D1B1_2
 A1D1B2_2
 A2D1B2_2
 D1D1B2_2
 D2D1B2_2
 D3D1B2_2
 B1D1B2_2
 B2D1B2_2);
A1D1A1_2 = ((A1D1_2)*(D1A1_2))*1000 ;
 A2D1A1_2 = ((A2D1_2)*(D1A1_2))*1000 ;
```

```
D1D1A1_2 = ((D1D1_2)*(D1A1_2))*1000 ;
D2D1A1_2 = ((D2D1_2)*(D1A1_2))*1000 ;
D3D1A1_2 = ((D3D1_2)*(D1A1_2))*1000 ;
B1D1A1_2 = ((B1D1_2)*(D1A1_2))*1000 ;
B2D1A1_2 = ((B2D1_2)*(D1A1_2))*1000 ;
A1D1A2_2 = ((A1D1_2)*(D1A2_2))*1000 ;
A2D1A2_2 = ((A2D1_2)*(D1A2_2))*1000 ;
D1D1A2_2 = ((D1D1_2)*(D1A2_2))*1000 ;
D2D1A2_2 = ((D2D1_2)*(D1A2_2))*1000 ;
D3D1A2_2 = ((D3D1_2)*(D1A2_2))*1000 ;
B1D1A2_2 = ((B1D1_2)*(D1A2_2))*1000 ;
B2D1A2_2 = ((B2D1_2)*(D1A2_2))*1000 ;
A1D1D1_2 = ((A1D1_2)*(D1D1_2))*1000 ;
A2D1D1_2 = ((A2D1_2)*(D1D1_2))*1000 ;
D1D1D1_2 = ((D1D1_2)*(D1D1_2))*1000 ;
D2D1D1_2 = ((D2D1_2)*(D1D1_2))*1000 ;
D3D1D1_2 = ((D3D1_2)*(D1D1_2))*1000 ;
B1D1D1_2 = ((B1D1_2)*(D1D1_2))*1000 ;
B2D1D1_2 = ((B2D1_2)*(D1D1_2))*1000 ;
A1D1D2_2 = ((A1D1_2)*(D1D2_2))*1000 ;
A2D1D2_2 = ((A2D1_2)*(D1D2_2))*1000 ;
D1D1D2_2 = ((D1D1_2)*(D1D2_2))*1000 ;
D2D1D2_2 = ((D2D1_2)*(D1D2_2))*1000 ;
D3D1D2_2 = ((D3D1_2)*(D1D2_2))*1000 ;
B1D1D2_2 = ((B1D1_2)*(D1D2_2))*1000 ;
B2D1D2_2 = ((B2D1_2)*(D1D2_2))*1000 ;
A1D1D3_2 = ((A1D1_2)*(D1D3_2))*1000 ;
A2D1D3_2 = ((A2D1_2)*(D1D3_2))*1000 ;
D1D1D3_2 = ((D1D1_2)*(D1D3_2))*1000 ;
D2D1D3_2 = ((D2D1_2)*(D1D3_2))*1000 ;
D3D1D3_2 = ((D3D1_2)*(D1D3_2))*1000 ;
B1D1D3_2 = ((B1D1_2)*(D1D3_2))*1000 ;
B2D1D3_2 = ((B2D1_2)*(D1D3_2))*1000 ;
A1D1B1_2 = ((A1D1_2)*(D1B1_2))*1000 ;
A2D1B1_2 = ((A2D1_2)*(D1B1_2))*1000 ;
D1D1B1_2 = ((D1D1_2)*(D1B1_2))*1000 ;
D2D1B1_2 = ((D2D1_2)*(D1B1_2))*1000 ;
D3D1B1_2 = ((D3D1_2)*(D1B1_2))*1000 ;
B1D1B1_2 = ((B1D1_2)*(D1B1_2))*1000 ;
B2D1B1_2 = ((B2D1_2)*(D1B1_2))*1000 ;
A1D1B2_2 = ((A1D1_2)*(D1B2_2))*1000 ;
A2D1B2_2 = ((A2D1_2)*(D1B2_2))*1000 ;
D1D1B2_2 = ((D1D1_2)*(D1B2_2))*1000 ;
D2D1B2_2 = ((D2D1_2)*(D1B2_2))*1000 ;
D3D1B2_2 = ((D3D1_2)*(D1B2_2))*1000 ;
B1D1B2_2 = ((B1D1_2)*(D1B2_2))*1000 ;
B2D1B2_2 = ((B2D1_2)*(D1B2_2))*1000 ;

! indirect effects D2 group 0
```

NEW(A1D2A1_0
A2D2A1_0
D1D2A1_0
D2D2A1_0
D3D2A1_0
B1D2A1_0
B2D2A1_0
A1D2A2_0
A2D2A2_0
D1D2A2_0
D2D2A2_0
D3D2A2_0
B1D2A2_0
B2D2A2_0
A1D2D1_0
A2D2D1_0
D1D2D1_0
D2D2D1_0
D3D2D1_0
B1D2D1_0
B2D2D1_0
A1D2D2_0
A2D2D2_0
D1D2D2_0
D2D2D2_0
D3D2D2_0
B1D2D2_0
B2D2D2_0
A1D2D3_0
A2D2D3_0
D1D2D3_0
D2D2D3_0
D3D2D3_0
B1D2D3_0
B2D2D3_0
A1D2B1_0
A2D2B1_0
D1D2B1_0
D2D2B1_0
D3D2B1_0
B1D2B1_0
B2D2B1_0
A1D2B2_0
A2D2B2_0
D1D2B2_0
D2D2B2_0
D3D2B2_0
B1D2B2_0
B2D2B2_0);

```
A1D2A1_0 = ((A1D2_0)*(D2A1_0))*1000 ;
A2D2A1_0 = ((A2D2_0)*(D2A1_0))*1000 ;
D1D2A1_0 = ((D1D2_0)*(D2A1_0))*1000 ;
D2D2A1_0 = ((D2D2_0)*(D2A1_0))*1000 ;
D3D2A1_0 = ((D3D2_0)*(D2A1_0))*1000 ;
B1D2A1_0 = ((B1D2_0)*(D2A1_0))*1000 ;
B2D2A1_0 = ((B2D2_0)*(D2A1_0))*1000 ;
A1D2A2_0 = ((A1D2_0)*(D2A2_0))*1000 ;
A2D2A2_0 = ((A2D2_0)*(D2A2_0))*1000 ;
D1D2A2_0 = ((D1D2_0)*(D2A2_0))*1000 ;
D2D2A2_0 = ((D2D2_0)*(D2A2_0))*1000 ;
D3D2A2_0 = ((D3D2_0)*(D2A2_0))*1000 ;
B1D2A2_0 = ((B1D2_0)*(D2A2_0))*1000 ;
B2D2A2_0 = ((B2D2_0)*(D2A2_0))*1000 ;
A1D2D1_0 = ((A1D2_0)*(D2D1_0))*1000 ;
A2D2D1_0 = ((A2D2_0)*(D2D1_0))*1000 ;
D1D2D1_0 = ((D1D2_0)*(D2D1_0))*1000 ;
D2D2D1_0 = ((D2D2_0)*(D2D1_0))*1000 ;
D3D2D1_0 = ((D3D2_0)*(D2D1_0))*1000 ;
B1D2D1_0 = ((B1D2_0)*(D2D1_0))*1000 ;
B2D2D1_0 = ((B2D2_0)*(D2D1_0))*1000 ;
A1D2D2_0 = ((A1D2_0)*(D2D2_0))*1000 ;
A2D2D2_0 = ((A2D2_0)*(D2D2_0))*1000 ;
D1D2D2_0 = ((D1D2_0)*(D2D2_0))*1000 ;
D2D2D2_0 = ((D2D2_0)*(D2D2_0))*1000 ;
D3D2D2_0 = ((D3D2_0)*(D2D2_0))*1000 ;
B1D2D2_0 = ((B1D2_0)*(D2D2_0))*1000 ;
B2D2D2_0 = ((B2D2_0)*(D2D2_0))*1000 ;
A1D2D3_0 = ((A1D2_0)*(D2D3_0))*1000 ;
A2D2D3_0 = ((A2D2_0)*(D2D3_0))*1000 ;
D1D2D3_0 = ((D1D2_0)*(D2D3_0))*1000 ;
D2D2D3_0 = ((D2D2_0)*(D2D3_0))*1000 ;
D3D2D3_0 = ((D3D2_0)*(D2D3_0))*1000 ;
B1D2D3_0 = ((B1D2_0)*(D2D3_0))*1000 ;
B2D2D3_0 = ((B2D2_0)*(D2D3_0))*1000 ;
A1D2B1_0 = ((A1D2_0)*(D2B1_0))*1000 ;
A2D2B1_0 = ((A2D2_0)*(D2B1_0))*1000 ;
D1D2B1_0 = ((D1D2_0)*(D2B1_0))*1000 ;
D2D2B1_0 = ((D2D2_0)*(D2B1_0))*1000 ;
D3D2B1_0 = ((D3D2_0)*(D2B1_0))*1000 ;
B1D2B1_0 = ((B1D2_0)*(D2B1_0))*1000 ;
B2D2B1_0 = ((B2D2_0)*(D2B1_0))*1000 ;
A1D2B2_0 = ((A1D2_0)*(D2B2_0))*1000 ;
A2D2B2_0 = ((A2D2_0)*(D2B2_0))*1000 ;
D1D2B2_0 = ((D1D2_0)*(D2B2_0))*1000 ;
D2D2B2_0 = ((D2D2_0)*(D2B2_0))*1000 ;
D3D2B2_0 = ((D3D2_0)*(D2B2_0))*1000 ;
B1D2B2_0 = ((B1D2_0)*(D2B2_0))*1000 ;
B2D2B2_0 = ((B2D2_0)*(D2B2_0))*1000 ;
```

! indirect effects D2 group 1
NEW(A1D2A1_1
A2D2A1_1
D1D2A1_1
D2D2A1_1
D3D2A1_1
B1D2A1_1
B2D2A1_1
A1D2A2_1
A2D2A2_1
D1D2A2_1
D2D2A2_1
D3D2A2_1
B1D2A2_1
B2D2A2_1
A1D2D1_1
A2D2D1_1
D1D2D1_1
D2D2D1_1
D3D2D1_1
B1D2D1_1
B2D2D1_1
A1D2D2_1
A2D2D2_1
D1D2D2_1
D2D2D2_1
D3D2D2_1
B1D2D2_1
B2D2D2_1
A1D2D3_1
A2D2D3_1
D1D2D3_1
D2D2D3_1
D3D2D3_1
B1D2D3_1
B2D2D3_1
A1D2B1_1
A2D2B1_1
D1D2B1_1
D2D2B1_1
D3D2B1_1
B1D2B1_1
B2D2B1_1
A1D2B2_1
A2D2B2_1
D1D2B2_1
D2D2B2_1
D3D2B2_1

```
 B1D2B2_1
 B2D2B2_1);
A1D2A1_1 = ((A1D2_1)*(D2A1_1))*1000 ;
 A2D2A1_1 = ((A2D2_1)*(D2A1_1))*1000 ;
 D1D2A1_1 = ((D1D2_1)*(D2A1_1))*1000 ;
 D2D2A1_1 = ((D2D2_1)*(D2A1_1))*1000 ;
 D3D2A1_1 = ((D3D2_1)*(D2A1_1))*1000 ;
 B1D2A1_1 = ((B1D2_1)*(D2A1_1))*1000 ;
 B2D2A1_1 = ((B2D2_1)*(D2A1_1))*1000 ;
 A1D2A2_1 = ((A1D2_1)*(D2A2_1))*1000 ;
 A2D2A2_1 = ((A2D2_1)*(D2A2_1))*1000 ;
 D1D2A2_1 = ((D1D2_1)*(D2A2_1))*1000 ;
 D2D2A2_1 = ((D2D2_1)*(D2A2_1))*1000 ;
 D3D2A2_1 = ((D3D2_1)*(D2A2_1))*1000 ;
 B1D2A2_1 = ((B1D2_1)*(D2A2_1))*1000 ;
 B2D2A2_1 = ((B2D2_1)*(D2A2_1))*1000 ;
 A1D2D1_1 = ((A1D2_1)*(D2D1_1))*1000 ;
 A2D2D1_1 = ((A2D2_1)*(D2D1_1))*1000 ;
 D1D2D1_1 = ((D1D2_1)*(D2D1_1))*1000 ;
 D2D2D1_1 = ((D2D2_1)*(D2D1_1))*1000 ;
 D3D2D1_1 = ((D3D2_1)*(D2D1_1))*1000 ;
 B1D2D1_1 = ((B1D2_1)*(D2D1_1))*1000 ;
 B2D2D1_1 = ((B2D2_1)*(D2D1_1))*1000 ;
 A1D2D2_1 = ((A1D2_1)*(D2D2_1))*1000 ;
 A2D2D2_1 = ((A2D2_1)*(D2D2_1))*1000 ;
 D1D2D2_1 = ((D1D2_1)*(D2D2_1))*1000 ;
 D2D2D2_1 = ((D2D2_1)*(D2D2_1))*1000 ;
 D3D2D2_1 = ((D3D2_1)*(D2D2_1))*1000 ;
 B1D2D2_1 = ((B1D2_1)*(D2D2_1))*1000 ;
 B2D2D2_1 = ((B2D2_1)*(D2D2_1))*1000 ;
 A1D2D3_1 = ((A1D2_1)*(D2D3_1))*1000 ;
 A2D2D3_1 = ((A2D2_1)*(D2D3_1))*1000 ;
 D1D2D3_1 = ((D1D2_1)*(D2D3_1))*1000 ;
 D2D2D3_1 = ((D2D2_1)*(D2D3_1))*1000 ;
 D3D2D3_1 = ((D3D2_1)*(D2D3_1))*1000 ;
 B1D2D3_1 = ((B1D2_1)*(D2D3_1))*1000 ;
 B2D2D3_1 = ((B2D2_1)*(D2D3_1))*1000 ;
 A1D2B1_1 = ((A1D2_1)*(D2B1_1))*1000 ;
 A2D2B1_1 = ((A2D2_1)*(D2B1_1))*1000 ;
 D1D2B1_1 = ((D1D2_1)*(D2B1_1))*1000 ;
 D2D2B1_1 = ((D2D2_1)*(D2B1_1))*1000 ;
 D3D2B1_1 = ((D3D2_1)*(D2B1_1))*1000 ;
 B1D2B1_1 = ((B1D2_1)*(D2B1_1))*1000 ;
 B2D2B1_1 = ((B2D2_1)*(D2B1_1))*1000 ;
 A1D2B2_1 = ((A1D2_1)*(D2B2_1))*1000 ;
 A2D2B2_1 = ((A2D2_1)*(D2B2_1))*1000 ;
 D1D2B2_1 = ((D1D2_1)*(D2B2_1))*1000 ;
 D2D2B2_1 = ((D2D2_1)*(D2B2_1))*1000 ;
 D3D2B2_1 = ((D3D2_1)*(D2B2_1))*1000 ;
```

```
B1D2B2_1 = ((B1D2_1)*(D2B2_1))*1000 ;
B2D2B2_1 = ((B2D2_1)*(D2B2_1))*1000 ;

! indirect effects D2 group 2
NEW(A1D2A1_2
A2D2A1_2
D1D2A1_2
D2D2A1_2
D3D2A1_2
B1D2A1_2
B2D2A1_2
A1D2A2_2
A2D2A2_2
D1D2A2_2
D2D2A2_2
D3D2A2_2
B1D2A2_2
B2D2A2_2
A1D2D1_2
A2D2D1_2
D1D2D1_2
D2D2D1_2
D3D2D1_2
B1D2D1_2
B2D2D1_2
A1D2D2_2
A2D2D2_2
D1D2D2_2
D2D2D2_2
D3D2D2_2
B1D2D2_2
B2D2D2_2
A1D2D3_2
A2D2D3_2
D1D2D3_2
D2D2D3_2
D3D2D3_2
B1D2D3_2
B2D2D3_2
A1D2B1_2
A2D2B1_2
D1D2B1_2
D2D2B1_2
D3D2B1_2
B1D2B1_2
B2D2B1_2
A1D2B2_2
A2D2B2_2
D1D2B2_2
```

```
 D2D2B2_2
 D3D2B2_2
 B1D2B2_2
 B2D2B2_2);
A1D2A1_2 = ((A1D2_2)*(D2A1_2))*1000 ;
 A2D2A1_2 = ((A2D2_2)*(D2A1_2))*1000 ;
 D1D2A1_2 = ((D1D2_2)*(D2A1_2))*1000 ;
 D2D2A1_2 = ((D2D2_2)*(D2A1_2))*1000 ;
 D3D2A1_2 = ((D3D2_2)*(D2A1_2))*1000 ;
 B1D2A1_2 = ((B1D2_2)*(D2A1_2))*1000 ;
 B2D2A1_2 = ((B2D2_2)*(D2A1_2))*1000 ;
 A1D2A2_2 = ((A1D2_2)*(D2A2_2))*1000 ;
 A2D2A2_2 = ((A2D2_2)*(D2A2_2))*1000 ;
 D1D2A2_2 = ((D1D2_2)*(D2A2_2))*1000 ;
 D2D2A2_2 = ((D2D2_2)*(D2A2_2))*1000 ;
 D3D2A2_2 = ((D3D2_2)*(D2A2_2))*1000 ;
 B1D2A2_2 = ((B1D2_2)*(D2A2_2))*1000 ;
 B2D2A2_2 = ((B2D2_2)*(D2A2_2))*1000 ;
 A1D2D1_2 = ((A1D2_2)*(D2D1_2))*1000 ;
 A2D2D1_2 = ((A2D2_2)*(D2D1_2))*1000 ;
 D1D2D1_2 = ((D1D2_2)*(D2D1_2))*1000 ;
 D2D2D1_2 = ((D2D2_2)*(D2D1_2))*1000 ;
 D3D2D1_2 = ((D3D2_2)*(D2D1_2))*1000 ;
 B1D2D1_2 = ((B1D2_2)*(D2D1_2))*1000 ;
 B2D2D1_2 = ((B2D2_2)*(D2D1_2))*1000 ;
 A1D2D2_2 = ((A1D2_2)*(D2D2_2))*1000 ;
 A2D2D2_2 = ((A2D2_2)*(D2D2_2))*1000 ;
 D1D2D2_2 = ((D1D2_2)*(D2D2_2))*1000 ;
 D2D2D2_2 = ((D2D2_2)*(D2D2_2))*1000 ;
 D3D2D2_2 = ((D3D2_2)*(D2D2_2))*1000 ;
 B1D2D2_2 = ((B1D2_2)*(D2D2_2))*1000 ;
 B2D2D2_2 = ((B2D2_2)*(D2D2_2))*1000 ;
 A1D2D3_2 = ((A1D2_2)*(D2D3_2))*1000 ;
 A2D2D3_2 = ((A2D2_2)*(D2D3_2))*1000 ;
 D1D2D3_2 = ((D1D2_2)*(D2D3_2))*1000 ;
 D2D2D3_2 = ((D2D2_2)*(D2D3_2))*1000 ;
 D3D2D3_2 = ((D3D2_2)*(D2D3_2))*1000 ;
 B1D2D3_2 = ((B1D2_2)*(D2D3_2))*1000 ;
 B2D2D3_2 = ((B2D2_2)*(D2D3_2))*1000 ;
 A1D2B1_2 = ((A1D2_2)*(D2B1_2))*1000 ;
 A2D2B1_2 = ((A2D2_2)*(D2B1_2))*1000 ;
 D1D2B1_2 = ((D1D2_2)*(D2B1_2))*1000 ;
 D2D2B1_2 = ((D2D2_2)*(D2B1_2))*1000 ;
 D3D2B1_2 = ((D3D2_2)*(D2B1_2))*1000 ;
 B1D2B1_2 = ((B1D2_2)*(D2B1_2))*1000 ;
 B2D2B1_2 = ((B2D2_2)*(D2B1_2))*1000 ;
 A1D2B2_2 = ((A1D2_2)*(D2B2_2))*1000 ;
 A2D2B2_2 = ((A2D2_2)*(D2B2_2))*1000 ;
 D1D2B2_2 = ((D1D2_2)*(D2B2_2))*1000 ;
```

```
D2D2B2_2 = ((D2D2_2)*(D2B2_2))*1000 ;
D3D2B2_2 = ((D3D2_2)*(D2B2_2))*1000 ;
B1D2B2_2 = ((B1D2_2)*(D2B2_2))*1000 ;
B2D2B2_2 = ((B2D2_2)*(D2B2_2))*1000 ;

! indirect effects D3 group 0
NEW(A1D3A1_0
A2D3A1_0
D1D3A1_0
D2D3A1_0
D3D3A1_0
B1D3A1_0
B2D3A1_0
A1D3A2_0
A2D3A2_0
D1D3A2_0
D2D3A2_0
D3D3A2_0
B1D3A2_0
B2D3A2_0
A1D3D1_0
A2D3D1_0
D1D3D1_0
D2D3D1_0
D3D3D1_0
B1D3D1_0
B2D3D1_0
A1D3D2_0
A2D3D2_0
D1D3D2_0
D2D3D2_0
D3D3D2_0
B1D3D2_0
B2D3D2_0
A1D3D3_0
A2D3D3_0
D1D3D3_0
D2D3D3_0
D3D3D3_0
B1D3D3_0
B2D3D3_0
A1D3B1_0
A2D3B1_0
D1D3B1_0
D2D3B1_0
D3D3B1_0
B1D3B1_0
B2D3B1_0
A1D3B2_0
```

```
 A2D3B2_0
 D1D3B2_0
 D2D3B2_0
 D3D3B2_0
 B1D3B2_0
 B2D3B2_0);
A1D3A1_0 = ((A1D3_0)*(D3A1_0))*1000 ;
 A2D3A1_0 = ((A2D3_0)*(D3A1_0))*1000 ;
 D1D3A1_0 = ((D1D3_0)*(D3A1_0))*1000 ;
 D2D3A1_0 = ((D2D3_0)*(D3A1_0))*1000 ;
 D3D3A1_0 = ((D3D3_0)*(D3A1_0))*1000 ;
 B1D3A1_0 = ((B1D3_0)*(D3A1_0))*1000 ;
 B2D3A1_0 = ((B2D3_0)*(D3A1_0))*1000 ;
 A1D3A2_0 = ((A1D3_0)*(D3A2_0))*1000 ;
 A2D3A2_0 = ((A2D3_0)*(D3A2_0))*1000 ;
 D1D3A2_0 = ((D1D3_0)*(D3A2_0))*1000 ;
 D2D3A2_0 = ((D2D3_0)*(D3A2_0))*1000 ;
 D3D3A2_0 = ((D3D3_0)*(D3A2_0))*1000 ;
 B1D3A2_0 = ((B1D3_0)*(D3A2_0))*1000 ;
 B2D3A2_0 = ((B2D3_0)*(D3A2_0))*1000 ;
 A1D3D1_0 = ((A1D3_0)*(D3D1_0))*1000 ;
 A2D3D1_0 = ((A2D3_0)*(D3D1_0))*1000 ;
 D1D3D1_0 = ((D1D3_0)*(D3D1_0))*1000 ;
 D2D3D1_0 = ((D2D3_0)*(D3D1_0))*1000 ;
 D3D3D1_0 = ((D3D3_0)*(D3D1_0))*1000 ;
 B1D3D1_0 = ((B1D3_0)*(D3D1_0))*1000 ;
 B2D3D1_0 = ((B2D3_0)*(D3D1_0))*1000 ;
 A1D3D2_0 = ((A1D3_0)*(D3D2_0))*1000 ;
 A2D3D2_0 = ((A2D3_0)*(D3D2_0))*1000 ;
 D1D3D2_0 = ((D1D3_0)*(D3D2_0))*1000 ;
 D2D3D2_0 = ((D2D3_0)*(D3D2_0))*1000 ;
 D3D3D2_0 = ((D3D3_0)*(D3D2_0))*1000 ;
 B1D3D2_0 = ((B1D3_0)*(D3D2_0))*1000 ;
 B2D3D2_0 = ((B2D3_0)*(D3D2_0))*1000 ;
 A1D3D3_0 = ((A1D3_0)*(D3D3_0))*1000 ;
 A2D3D3_0 = ((A2D3_0)*(D3D3_0))*1000 ;
 D1D3D3_0 = ((D1D3_0)*(D3D3_0))*1000 ;
 D2D3D3_0 = ((D2D3_0)*(D3D3_0))*1000 ;
 D3D3D3_0 = ((D3D3_0)*(D3D3_0))*1000 ;
 B1D3D3_0 = ((B1D3_0)*(D3D3_0))*1000 ;
 B2D3D3_0 = ((B2D3_0)*(D3D3_0))*1000 ;
 A1D3B1_0 = ((A1D3_0)*(D3B1_0))*1000 ;
 A2D3B1_0 = ((A2D3_0)*(D3B1_0))*1000 ;
 D1D3B1_0 = ((D1D3_0)*(D3B1_0))*1000 ;
 D2D3B1_0 = ((D2D3_0)*(D3B1_0))*1000 ;
 D3D3B1_0 = ((D3D3_0)*(D3B1_0))*1000 ;
 B1D3B1_0 = ((B1D3_0)*(D3B1_0))*1000 ;
 B2D3B1_0 = ((B2D3_0)*(D3B1_0))*1000 ;
 A1D3B2_0 = ((A1D3_0)*(D3B2_0))*1000 ;
```

```
A2D3B2_0 = ((A2D3_0)*(D3B2_0))*1000 ;
D1D3B2_0 = ((D1D3_0)*(D3B2_0))*1000 ;
D2D3B2_0 = ((D2D3_0)*(D3B2_0))*1000 ;
D3D3B2_0 = ((D3D3_0)*(D3B2_0))*1000 ;
B1D3B2_0 = ((B1D3_0)*(D3B2_0))*1000 ;
B2D3B2_0 = ((B2D3_0)*(D3B2_0))*1000 ;

! indirect effects D3 group 1
NEW(A1D3A1_1
A2D3A1_1
D1D3A1_1
D2D3A1_1
D3D3A1_1
B1D3A1_1
B2D3A1_1
A1D3A2_1
A2D3A2_1
D1D3A2_1
D2D3A2_1
D3D3A2_1
B1D3A2_1
B2D3A2_1
A1D3D1_1
A2D3D1_1
D1D3D1_1
D2D3D1_1
D3D3D1_1
B1D3D1_1
B2D3D1_1
A1D3D2_1
A2D3D2_1
D1D3D2_1
D2D3D2_1
D3D3D2_1
B1D3D2_1
B2D3D2_1
A1D3D3_1
A2D3D3_1
D1D3D3_1
D2D3D3_1
D3D3D3_1
B1D3D3_1
B2D3D3_1
A1D3B1_1
A2D3B1_1
D1D3B1_1
D2D3B1_1
D3D3B1_1
B1D3B1_1
```

```
 B2D3B1_1
 A1D3B2_1
 A2D3B2_1
 D1D3B2_1
 D2D3B2_1
 D3D3B2_1
 B1D3B2_1
 B2D3B2_1);
A1D3A1_1 = ((A1D3_1)*(D3A1_1))*1000 ;
 A2D3A1_1 = ((A2D3_1)*(D3A1_1))*1000 ;
 D1D3A1_1 = ((D1D3_1)*(D3A1_1))*1000 ;
 D2D3A1_1 = ((D2D3_1)*(D3A1_1))*1000 ;
 D3D3A1_1 = ((D3D3_1)*(D3A1_1))*1000 ;
 B1D3A1_1 = ((B1D3_1)*(D3A1_1))*1000 ;
 B2D3A1_1 = ((B2D3_1)*(D3A1_1))*1000 ;
 A1D3A2_1 = ((A1D3_1)*(D3A2_1))*1000 ;
 A2D3A2_1 = ((A2D3_1)*(D3A2_1))*1000 ;
 D1D3A2_1 = ((D1D3_1)*(D3A2_1))*1000 ;
 D2D3A2_1 = ((D2D3_1)*(D3A2_1))*1000 ;
 D3D3A2_1 = ((D3D3_1)*(D3A2_1))*1000 ;
 B1D3A2_1 = ((B1D3_1)*(D3A2_1))*1000 ;
 B2D3A2_1 = ((B2D3_1)*(D3A2_1))*1000 ;
 A1D3D1_1 = ((A1D3_1)*(D3D1_1))*1000 ;
 A2D3D1_1 = ((A2D3_1)*(D3D1_1))*1000 ;
 D1D3D1_1 = ((D1D3_1)*(D3D1_1))*1000 ;
 D2D3D1_1 = ((D2D3_1)*(D3D1_1))*1000 ;
 D3D3D1_1 = ((D3D3_1)*(D3D1_1))*1000 ;
 B1D3D1_1 = ((B1D3_1)*(D3D1_1))*1000 ;
 B2D3D1_1 = ((B2D3_1)*(D3D1_1))*1000 ;
 A1D3D2_1 = ((A1D3_1)*(D3D2_1))*1000 ;
 A2D3D2_1 = ((A2D3_1)*(D3D2_1))*1000 ;
 D1D3D2_1 = ((D1D3_1)*(D3D2_1))*1000 ;
 D2D3D2_1 = ((D2D3_1)*(D3D2_1))*1000 ;
 D3D3D2_1 = ((D3D3_1)*(D3D2_1))*1000 ;
 B1D3D2_1 = ((B1D3_1)*(D3D2_1))*1000 ;
 B2D3D2_1 = ((B2D3_1)*(D3D2_1))*1000 ;
 A1D3D3_1 = ((A1D3_1)*(D3D3_1))*1000 ;
 A2D3D3_1 = ((A2D3_1)*(D3D3_1))*1000 ;
 D1D3D3_1 = ((D1D3_1)*(D3D3_1))*1000 ;
 D2D3D3_1 = ((D2D3_1)*(D3D3_1))*1000 ;
 D3D3D3_1 = ((D3D3_1)*(D3D3_1))*1000 ;
 B1D3D3_1 = ((B1D3_1)*(D3D3_1))*1000 ;
 B2D3D3_1 = ((B2D3_1)*(D3D3_1))*1000 ;
 A1D3B1_1 = ((A1D3_1)*(D3B1_1))*1000 ;
 A2D3B1_1 = ((A2D3_1)*(D3B1_1))*1000 ;
 D1D3B1_1 = ((D1D3_1)*(D3B1_1))*1000 ;
 D2D3B1_1 = ((D2D3_1)*(D3B1_1))*1000 ;
 D3D3B1_1 = ((D3D3_1)*(D3B1_1))*1000 ;
 B1D3B1_1 = ((B1D3_1)*(D3B1_1))*1000 ;
```

```
B2D3B1_1 = ((B2D3_1)*(D3B1_1))*1000 ;
A1D3B2_1 = ((A1D3_1)*(D3B2_1))*1000 ;
A2D3B2_1 = ((A2D3_1)*(D3B2_1))*1000 ;
D1D3B2_1 = ((D1D3_1)*(D3B2_1))*1000 ;
D2D3B2_1 = ((D2D3_1)*(D3B2_1))*1000 ;
D3D3B2_1 = ((D3D3_1)*(D3B2_1))*1000 ;
B1D3B2_1 = ((B1D3_1)*(D3B2_1))*1000 ;
B2D3B2_1 = ((B2D3_1)*(D3B2_1))*1000 ;

! indirect effects D3 group 2
NEW(A1D3A1_2
A2D3A1_2
D1D3A1_2
D2D3A1_2
D3D3A1_2
B1D3A1_2
B2D3A1_2
A1D3A2_2
A2D3A2_2
D1D3A2_2
D2D3A2_2
D3D3A2_2
B1D3A2_2
B2D3A2_2
A1D3D1_2
A2D3D1_2
D1D3D1_2
D2D3D1_2
D3D3D1_2
B1D3D1_2
B2D3D1_2
A1D3D2_2
A2D3D2_2
D1D3D2_2
D2D3D2_2
D3D3D2_2
B1D3D2_2
B2D3D2_2
A1D3D3_2
A2D3D3_2
D1D3D3_2
D2D3D3_2
D3D3D3_2
B1D3D3_2
B2D3D3_2
A1D3B1_2
A2D3B1_2
D1D3B1_2
D2D3B1_2
```

```
 D3D3B1_2
 B1D3B1_2
 B2D3B1_2
 A1D3B2_2
 A2D3B2_2
 D1D3B2_2
 D2D3B2_2
 D3D3B2_2
 B1D3B2_2
 B2D3B2_2);
A1D3A1_2 = ((A1D3_2)*(D3A1_2))*1000 ;
 A2D3A1_2 = ((A2D3_2)*(D3A1_2))*1000 ;
 D1D3A1_2 = ((D1D3_2)*(D3A1_2))*1000 ;
 D2D3A1_2 = ((D2D3_2)*(D3A1_2))*1000 ;
 D3D3A1_2 = ((D3D3_2)*(D3A1_2))*1000 ;
 B1D3A1_2 = ((B1D3_2)*(D3A1_2))*1000 ;
 B2D3A1_2 = ((B2D3_2)*(D3A1_2))*1000 ;
 A1D3A2_2 = ((A1D3_2)*(D3A2_2))*1000 ;
 A2D3A2_2 = ((A2D3_2)*(D3A2_2))*1000 ;
 D1D3A2_2 = ((D1D3_2)*(D3A2_2))*1000 ;
 D2D3A2_2 = ((D2D3_2)*(D3A2_2))*1000 ;
 D3D3A2_2 = ((D3D3_2)*(D3A2_2))*1000 ;
 B1D3A2_2 = ((B1D3_2)*(D3A2_2))*1000 ;
 B2D3A2_2 = ((B2D3_2)*(D3A2_2))*1000 ;
 A1D3D1_2 = ((A1D3_2)*(D3D1_2))*1000 ;
 A2D3D1_2 = ((A2D3_2)*(D3D1_2))*1000 ;
 D1D3D1_2 = ((D1D3_2)*(D3D1_2))*1000 ;
 D2D3D1_2 = ((D2D3_2)*(D3D1_2))*1000 ;
 D3D3D1_2 = ((D3D3_2)*(D3D1_2))*1000 ;
 B1D3D1_2 = ((B1D3_2)*(D3D1_2))*1000 ;
 B2D3D1_2 = ((B2D3_2)*(D3D1_2))*1000 ;
 A1D3D2_2 = ((A1D3_2)*(D3D2_2))*1000 ;
 A2D3D2_2 = ((A2D3_2)*(D3D2_2))*1000 ;
 D1D3D2_2 = ((D1D3_2)*(D3D2_2))*1000 ;
 D2D3D2_2 = ((D2D3_2)*(D3D2_2))*1000 ;
 D3D3D2_2 = ((D3D3_2)*(D3D2_2))*1000 ;
 B1D3D2_2 = ((B1D3_2)*(D3D2_2))*1000 ;
 B2D3D2_2 = ((B2D3_2)*(D3D2_2))*1000 ;
 A1D3D3_2 = ((A1D3_2)*(D3D3_2))*1000 ;
 A2D3D3_2 = ((A2D3_2)*(D3D3_2))*1000 ;
 D1D3D3_2 = ((D1D3_2)*(D3D3_2))*1000 ;
 D2D3D3_2 = ((D2D3_2)*(D3D3_2))*1000 ;
 D3D3D3_2 = ((D3D3_2)*(D3D3_2))*1000 ;
 B1D3D3_2 = ((B1D3_2)*(D3D3_2))*1000 ;
 B2D3D3_2 = ((B2D3_2)*(D3D3_2))*1000 ;
 A1D3B1_2 = ((A1D3_2)*(D3B1_2))*1000 ;
 A2D3B1_2 = ((A2D3_2)*(D3B1_2))*1000 ;
 D1D3B1_2 = ((D1D3_2)*(D3B1_2))*1000 ;
 D2D3B1_2 = ((D2D3_2)*(D3B1_2))*1000 ;
```

```
D3D3B1_2 = ((D3D3_2)*(D3B1_2))*1000 ;
B1D3B1_2 = ((B1D3_2)*(D3B1_2))*1000 ;
B2D3B1_2 = ((B2D3_2)*(D3B1_2))*1000 ;
A1D3B2_2 = ((A1D3_2)*(D3B2_2))*1000 ;
A2D3B2_2 = ((A2D3_2)*(D3B2_2))*1000 ;
D1D3B2_2 = ((D1D3_2)*(D3B2_2))*1000 ;
D2D3B2_2 = ((D2D3_2)*(D3B2_2))*1000 ;
D3D3B2_2 = ((D3D3_2)*(D3B2_2))*1000 ;
B1D3B2_2 = ((B1D3_2)*(D3B2_2))*1000 ;
B2D3B2_2 = ((B2D3_2)*(D3B2_2))*1000 ;

! indirect effects B1 group 0
NEW(A1B1A1_0
A2B1A1_0
D1B1A1_0
D2B1A1_0
D3B1A1_0
B1B1A1_0
B2B1A1_0
A1B1A2_0
A2B1A2_0
D1B1A2_0
D2B1A2_0
D3B1A2_0
B1B1A2_0
B2B1A2_0
A1B1D1_0
A2B1D1_0
D1B1D1_0
D2B1D1_0
D3B1D1_0
B1B1D1_0
B2B1D1_0
A1B1D2_0
A2B1D2_0
D1B1D2_0
D2B1D2_0
D3B1D2_0
B1B1D2_0
B2B1D2_0
A1B1D3_0
A2B1D3_0
D1B1D3_0
D2B1D3_0
D3B1D3_0
B1B1D3_0
B2B1D3_0
A1B1B1_0
A2B1B1_0
```

```
 D1B1B1_0
 D2B1B1_0
 D3B1B1_0
 B1B1B1_0
 B2B1B1_0
 A1B1B2_0
 A2B1B2_0
 D1B1B2_0
 D2B1B2_0
 D3B1B2_0
 B1B1B2_0
 B2B1B2_0);
A1B1A1_0 = ((A1B1_0)*(B1A1_0))*1000 ;
 A2B1A1_0 = ((A2B1_0)*(B1A1_0))*1000 ;
 D1B1A1_0 = ((D1B1_0)*(B1A1_0))*1000 ;
 D2B1A1_0 = ((D2B1_0)*(B1A1_0))*1000 ;
 D3B1A1_0 = ((D3B1_0)*(B1A1_0))*1000 ;
 B1B1A1_0 = ((B1B1_0)*(B1A1_0))*1000 ;
 B2B1A1_0 = ((B2B1_0)*(B1A1_0))*1000 ;
 A1B1A2_0 = ((A1B1_0)*(B1A2_0))*1000 ;
 A2B1A2_0 = ((A2B1_0)*(B1A2_0))*1000 ;
 D1B1A2_0 = ((D1B1_0)*(B1A2_0))*1000 ;
 D2B1A2_0 = ((D2B1_0)*(B1A2_0))*1000 ;
 D3B1A2_0 = ((D3B1_0)*(B1A2_0))*1000 ;
 B1B1A2_0 = ((B1B1_0)*(B1A2_0))*1000 ;
 B2B1A2_0 = ((B2B1_0)*(B1A2_0))*1000 ;
 A1B1D1_0 = ((A1B1_0)*(B1D1_0))*1000 ;
 A2B1D1_0 = ((A2B1_0)*(B1D1_0))*1000 ;
 D1B1D1_0 = ((D1B1_0)*(B1D1_0))*1000 ;
 D2B1D1_0 = ((D2B1_0)*(B1D1_0))*1000 ;
 D3B1D1_0 = ((D3B1_0)*(B1D1_0))*1000 ;
 B1B1D1_0 = ((B1B1_0)*(B1D1_0))*1000 ;
 B2B1D1_0 = ((B2B1_0)*(B1D1_0))*1000 ;
 A1B1D2_0 = ((A1B1_0)*(B1D2_0))*1000 ;
 A2B1D2_0 = ((A2B1_0)*(B1D2_0))*1000 ;
 D1B1D2_0 = ((D1B1_0)*(B1D2_0))*1000 ;
 D2B1D2_0 = ((D2B1_0)*(B1D2_0))*1000 ;
 D3B1D2_0 = ((D3B1_0)*(B1D2_0))*1000 ;
 B1B1D2_0 = ((B1B1_0)*(B1D2_0))*1000 ;
 B2B1D2_0 = ((B2B1_0)*(B1D2_0))*1000 ;
 A1B1D3_0 = ((A1B1_0)*(B1D3_0))*1000 ;
 A2B1D3_0 = ((A2B1_0)*(B1D3_0))*1000 ;
 D1B1D3_0 = ((D1B1_0)*(B1D3_0))*1000 ;
 D2B1D3_0 = ((D2B1_0)*(B1D3_0))*1000 ;
 D3B1D3_0 = ((D3B1_0)*(B1D3_0))*1000 ;
 B1B1D3_0 = ((B1B1_0)*(B1D3_0))*1000 ;
 B2B1D3_0 = ((B2B1_0)*(B1D3_0))*1000 ;
 A1B1B1_0 = ((A1B1_0)*(B1B1_0))*1000 ;
 A2B1B1_0 = ((A2B1_0)*(B1B1_0))*1000 ;
```

```
D1B1B1_0 = ((D1B1_0)*(B1B1_0))*1000 ;
D2B1B1_0 = ((D2B1_0)*(B1B1_0))*1000 ;
D3B1B1_0 = ((D3B1_0)*(B1B1_0))*1000 ;
B1B1B1_0 = ((B1B1_0)*(B1B1_0))*1000 ;
B2B1B1_0 = ((B2B1_0)*(B1B1_0))*1000 ;
A1B1B2_0 = ((A1B1_0)*(B1B2_0))*1000 ;
A2B1B2_0 = ((A2B1_0)*(B1B2_0))*1000 ;
D1B1B2_0 = ((D1B1_0)*(B1B2_0))*1000 ;
D2B1B2_0 = ((D2B1_0)*(B1B2_0))*1000 ;
D3B1B2_0 = ((D3B1_0)*(B1B2_0))*1000 ;
B1B1B2_0 = ((B1B1_0)*(B1B2_0))*1000 ;
B2B1B2_0 = ((B2B1_0)*(B1B2_0))*1000 ;

! indirect effects B1 group 1
NEW(A1B1A1_1
A2B1A1_1
D1B1A1_1
D2B1A1_1
D3B1A1_1
B1B1A1_1
B2B1A1_1
A1B1A2_1
A2B1A2_1
D1B1A2_1
D2B1A2_1
D3B1A2_1
B1B1A2_1
B2B1A2_1
A1B1D1_1
A2B1D1_1
D1B1D1_1
D2B1D1_1
D3B1D1_1
B1B1D1_1
B2B1D1_1
A1B1D2_1
A2B1D2_1
D1B1D2_1
D2B1D2_1
D3B1D2_1
B1B1D2_1
B2B1D2_1
A1B1D3_1
A2B1D3_1
D1B1D3_1
D2B1D3_1
D3B1D3_1
B1B1D3_1
B2B1D3_1
```

```
 A1B1B1_1
 A2B1B1_1
 D1B1B1_1
 D2B1B1_1
 D3B1B1_1
 B1B1B1_1
 B2B1B1_1
 A1B1B2_1
 A2B1B2_1
 D1B1B2_1
 D2B1B2_1
 D3B1B2_1
 B1B1B2_1
 B2B1B2_1);
A1B1A1_1 = ((A1B1_1)*(B1A1_1))*1000 ;
 A2B1A1_1 = ((A2B1_1)*(B1A1_1))*1000 ;
 D1B1A1_1 = ((D1B1_1)*(B1A1_1))*1000 ;
 D2B1A1_1 = ((D2B1_1)*(B1A1_1))*1000 ;
 D3B1A1_1 = ((D3B1_1)*(B1A1_1))*1000 ;
 B1B1A1_1 = ((B1B1_1)*(B1A1_1))*1000 ;
 B2B1A1_1 = ((B2B1_1)*(B1A1_1))*1000 ;
 A1B1A2_1 = ((A1B1_1)*(B1A2_1))*1000 ;
 A2B1A2_1 = ((A2B1_1)*(B1A2_1))*1000 ;
 D1B1A2_1 = ((D1B1_1)*(B1A2_1))*1000 ;
 D2B1A2_1 = ((D2B1_1)*(B1A2_1))*1000 ;
 D3B1A2_1 = ((D3B1_1)*(B1A2_1))*1000 ;
 B1B1A2_1 = ((B1B1_1)*(B1A2_1))*1000 ;
 B2B1A2_1 = ((B2B1_1)*(B1A2_1))*1000 ;
 A1B1D1_1 = ((A1B1_1)*(B1D1_1))*1000 ;
 A2B1D1_1 = ((A2B1_1)*(B1D1_1))*1000 ;
 D1B1D1_1 = ((D1B1_1)*(B1D1_1))*1000 ;
 D2B1D1_1 = ((D2B1_1)*(B1D1_1))*1000 ;
 D3B1D1_1 = ((D3B1_1)*(B1D1_1))*1000 ;
 B1B1D1_1 = ((B1B1_1)*(B1D1_1))*1000 ;
 B2B1D1_1 = ((B2B1_1)*(B1D1_1))*1000 ;
 A1B1D2_1 = ((A1B1_1)*(B1D2_1))*1000 ;
 A2B1D2_1 = ((A2B1_1)*(B1D2_1))*1000 ;
 D1B1D2_1 = ((D1B1_1)*(B1D2_1))*1000 ;
 D2B1D2_1 = ((D2B1_1)*(B1D2_1))*1000 ;
 D3B1D2_1 = ((D3B1_1)*(B1D2_1))*1000 ;
 B1B1D2_1 = ((B1B1_1)*(B1D2_1))*1000 ;
 B2B1D2_1 = ((B2B1_1)*(B1D2_1))*1000 ;
 A1B1D3_1 = ((A1B1_1)*(B1D3_1))*1000 ;
 A2B1D3_1 = ((A2B1_1)*(B1D3_1))*1000 ;
 D1B1D3_1 = ((D1B1_1)*(B1D3_1))*1000 ;
 D2B1D3_1 = ((D2B1_1)*(B1D3_1))*1000 ;
 D3B1D3_1 = ((D3B1_1)*(B1D3_1))*1000 ;
 B1B1D3_1 = ((B1B1_1)*(B1D3_1))*1000 ;
 B2B1D3_1 = ((B2B1_1)*(B1D3_1))*1000 ;
```

```
A1B1B1_1 = ((A1B1_1)*(B1B1_1))*1000 ;
A2B1B1_1 = ((A2B1_1)*(B1B1_1))*1000 ;
D1B1B1_1 = ((D1B1_1)*(B1B1_1))*1000 ;
D2B1B1_1 = ((D2B1_1)*(B1B1_1))*1000 ;
D3B1B1_1 = ((D3B1_1)*(B1B1_1))*1000 ;
B1B1B1_1 = ((B1B1_1)*(B1B1_1))*1000 ;
B2B1B1_1 = ((B2B1_1)*(B1B1_1))*1000 ;
A1B1B2_1 = ((A1B1_1)*(B1B2_1))*1000 ;
A2B1B2_1 = ((A2B1_1)*(B1B2_1))*1000 ;
D1B1B2_1 = ((D1B1_1)*(B1B2_1))*1000 ;
D2B1B2_1 = ((D2B1_1)*(B1B2_1))*1000 ;
D3B1B2_1 = ((D3B1_1)*(B1B2_1))*1000 ;
B1B1B2_1 = ((B1B1_1)*(B1B2_1))*1000 ;
B2B1B2_1 = ((B2B1_1)*(B1B2_1))*1000 ;

! indirect effects B1 group 2
NEW(A1B1A1_2
A2B1A1_2
D1B1A1_2
D2B1A1_2
D3B1A1_2
B1B1A1_2
B2B1A1_2
A1B1A2_2
A2B1A2_2
D1B1A2_2
D2B1A2_2
D3B1A2_2
B1B1A2_2
B2B1A2_2
A1B1D1_2
A2B1D1_2
D1B1D1_2
D2B1D1_2
D3B1D1_2
B1B1D1_2
B2B1D1_2
A1B1D2_2
A2B1D2_2
D1B1D2_2
D2B1D2_2
D3B1D2_2
B1B1D2_2
B2B1D2_2
A1B1D3_2
A2B1D3_2
D1B1D3_2
D2B1D3_2
D3B1D3_2
```

```
 B1B1D3_2
 B2B1D3_2
 A1B1B1_2
 A2B1B1_2
 D1B1B1_2
 D2B1B1_2
 D3B1B1_2
 B1B1B1_2
 B2B1B1_2
 A1B1B2_2
 A2B1B2_2
 D1B1B2_2
 D2B1B2_2
 D3B1B2_2
 B1B1B2_2
 B2B1B2_2);
A1B1A1_2 = ((A1B1_2)*(B1A1_2))*1000 ;
 A2B1A1_2 = ((A2B1_2)*(B1A1_2))*1000 ;
 D1B1A1_2 = ((D1B1_2)*(B1A1_2))*1000 ;
 D2B1A1_2 = ((D2B1_2)*(B1A1_2))*1000 ;
 D3B1A1_2 = ((D3B1_2)*(B1A1_2))*1000 ;
 B1B1A1_2 = ((B1B1_2)*(B1A1_2))*1000 ;
 B2B1A1_2 = ((B2B1_2)*(B1A1_2))*1000 ;
 A1B1A2_2 = ((A1B1_2)*(B1A2_2))*1000 ;
 A2B1A2_2 = ((A2B1_2)*(B1A2_2))*1000 ;
 D1B1A2_2 = ((D1B1_2)*(B1A2_2))*1000 ;
 D2B1A2_2 = ((D2B1_2)*(B1A2_2))*1000 ;
 D3B1A2_2 = ((D3B1_2)*(B1A2_2))*1000 ;
 B1B1A2_2 = ((B1B1_2)*(B1A2_2))*1000 ;
 B2B1A2_2 = ((B2B1_2)*(B1A2_2))*1000 ;
 A1B1D1_2 = ((A1B1_2)*(B1D1_2))*1000 ;
 A2B1D1_2 = ((A2B1_2)*(B1D1_2))*1000 ;
 D1B1D1_2 = ((D1B1_2)*(B1D1_2))*1000 ;
 D2B1D1_2 = ((D2B1_2)*(B1D1_2))*1000 ;
 D3B1D1_2 = ((D3B1_2)*(B1D1_2))*1000 ;
 B1B1D1_2 = ((B1B1_2)*(B1D1_2))*1000 ;
 B2B1D1_2 = ((B2B1_2)*(B1D1_2))*1000 ;
 A1B1D2_2 = ((A1B1_2)*(B1D2_2))*1000 ;
 A2B1D2_2 = ((A2B1_2)*(B1D2_2))*1000 ;
 D1B1D2_2 = ((D1B1_2)*(B1D2_2))*1000 ;
 D2B1D2_2 = ((D2B1_2)*(B1D2_2))*1000 ;
 D3B1D2_2 = ((D3B1_2)*(B1D2_2))*1000 ;
 B1B1D2_2 = ((B1B1_2)*(B1D2_2))*1000 ;
 B2B1D2_2 = ((B2B1_2)*(B1D2_2))*1000 ;
 A1B1D3_2 = ((A1B1_2)*(B1D3_2))*1000 ;
 A2B1D3_2 = ((A2B1_2)*(B1D3_2))*1000 ;
 D1B1D3_2 = ((D1B1_2)*(B1D3_2))*1000 ;
 D2B1D3_2 = ((D2B1_2)*(B1D3_2))*1000 ;
 D3B1D3_2 = ((D3B1_2)*(B1D3_2))*1000 ;
```

```
B1B1D3_2 = ((B1B1_2)*(B1D3_2))*1000 ;
B2B1D3_2 = ((B2B1_2)*(B1D3_2))*1000 ;
A1B1B1_2 = ((A1B1_2)*(B1B1_2))*1000 ;
A2B1B1_2 = ((A2B1_2)*(B1B1_2))*1000 ;
D1B1B1_2 = ((D1B1_2)*(B1B1_2))*1000 ;
D2B1B1_2 = ((D2B1_2)*(B1B1_2))*1000 ;
D3B1B1_2 = ((D3B1_2)*(B1B1_2))*1000 ;
B1B1B1_2 = ((B1B1_2)*(B1B1_2))*1000 ;
B2B1B1_2 = ((B2B1_2)*(B1B1_2))*1000 ;
A1B1B2_2 = ((A1B1_2)*(B1B2_2))*1000 ;
A2B1B2_2 = ((A2B1_2)*(B1B2_2))*1000 ;
D1B1B2_2 = ((D1B1_2)*(B1B2_2))*1000 ;
D2B1B2_2 = ((D2B1_2)*(B1B2_2))*1000 ;
D3B1B2_2 = ((D3B1_2)*(B1B2_2))*1000 ;
B1B1B2_2 = ((B1B1_2)*(B1B2_2))*1000 ;
B2B1B2_2 = ((B2B1_2)*(B1B2_2))*1000 ;

! indirect effects B2 group 0
NEW(A1B2A1_0
A2B2A1_0
D1B2A1_0
D2B2A1_0
D3B2A1_0
B1B2A1_0
B2B2A1_0
A1B2A2_0
A2B2A2_0
D1B2A2_0
D2B2A2_0
D3B2A2_0
B1B2A2_0
B2B2A2_0
A1B2D1_0
A2B2D1_0
D1B2D1_0
D2B2D1_0
D3B2D1_0
B1B2D1_0
B2B2D1_0
A1B2D2_0
A2B2D2_0
D1B2D2_0
D2B2D2_0
D3B2D2_0
B1B2D2_0
B2B2D2_0
A1B2D3_0
A2B2D3_0
D1B2D3_0
```

```
 D2B2D3_0
 D3B2D3_0
 B1B2D3_0
 B2B2D3_0
 A1B2B1_0
 A2B2B1_0
 D1B2B1_0
 D2B2B1_0
 D3B2B1_0
 B1B2B1_0
 B2B2B1_0
 A1B2B2_0
 A2B2B2_0
 D1B2B2_0
 D2B2B2_0
 D3B2B2_0
 B1B2B2_0
 B2B2B2_0);
A1B2A1_0 = ((A1B2_0)*(B2A1_0))*1000 ;
 A2B2A1_0 = ((A2B2_0)*(B2A1_0))*1000 ;
 D1B2A1_0 = ((D1B2_0)*(B2A1_0))*1000 ;
 D2B2A1_0 = ((D2B2_0)*(B2A1_0))*1000 ;
 D3B2A1_0 = ((D3B2_0)*(B2A1_0))*1000 ;
 B1B2A1_0 = ((B1B2_0)*(B2A1_0))*1000 ;
 B2B2A1_0 = ((B2B2_0)*(B2A1_0))*1000 ;
 A1B2A2_0 = ((A1B2_0)*(B2A2_0))*1000 ;
 A2B2A2_0 = ((A2B2_0)*(B2A2_0))*1000 ;
 D1B2A2_0 = ((D1B2_0)*(B2A2_0))*1000 ;
 D2B2A2_0 = ((D2B2_0)*(B2A2_0))*1000 ;
 D3B2A2_0 = ((D3B2_0)*(B2A2_0))*1000 ;
 B1B2A2_0 = ((B1B2_0)*(B2A2_0))*1000 ;
 B2B2A2_0 = ((B2B2_0)*(B2A2_0))*1000 ;
 A1B2D1_0 = ((A1B2_0)*(B2D1_0))*1000 ;
 A2B2D1_0 = ((A2B2_0)*(B2D1_0))*1000 ;
 D1B2D1_0 = ((D1B2_0)*(B2D1_0))*1000 ;
 D2B2D1_0 = ((D2B2_0)*(B2D1_0))*1000 ;
 D3B2D1_0 = ((D3B2_0)*(B2D1_0))*1000 ;
 B1B2D1_0 = ((B1B2_0)*(B2D1_0))*1000 ;
 B2B2D1_0 = ((B2B2_0)*(B2D1_0))*1000 ;
 A1B2D2_0 = ((A1B2_0)*(B2D2_0))*1000 ;
 A2B2D2_0 = ((A2B2_0)*(B2D2_0))*1000 ;
 D1B2D2_0 = ((D1B2_0)*(B2D2_0))*1000 ;
 D2B2D2_0 = ((D2B2_0)*(B2D2_0))*1000 ;
 D3B2D2_0 = ((D3B2_0)*(B2D2_0))*1000 ;
 B1B2D2_0 = ((B1B2_0)*(B2D2_0))*1000 ;
 B2B2D2_0 = ((B2B2_0)*(B2D2_0))*1000 ;
 A1B2D3_0 = ((A1B2_0)*(B2D3_0))*1000 ;
 A2B2D3_0 = ((A2B2_0)*(B2D3_0))*1000 ;
 D1B2D3_0 = ((D1B2_0)*(B2D3_0))*1000 ;
```

```
D2B2D3_0 = ((D2B2_0)*(B2D3_0))*1000 ;
D3B2D3_0 = ((D3B2_0)*(B2D3_0))*1000 ;
B1B2D3_0 = ((B1B2_0)*(B2D3_0))*1000 ;
B2B2D3_0 = ((B2B2_0)*(B2D3_0))*1000 ;
A1B2B1_0 = ((A1B2_0)*(B2B1_0))*1000 ;
A2B2B1_0 = ((A2B2_0)*(B2B1_0))*1000 ;
D1B2B1_0 = ((D1B2_0)*(B2B1_0))*1000 ;
D2B2B1_0 = ((D2B2_0)*(B2B1_0))*1000 ;
D3B2B1_0 = ((D3B2_0)*(B2B1_0))*1000 ;
B1B2B1_0 = ((B1B2_0)*(B2B1_0))*1000 ;
B2B2B1_0 = ((B2B2_0)*(B2B1_0))*1000 ;
A1B2B2_0 = ((A1B2_0)*(B2B2_0))*1000 ;
A2B2B2_0 = ((A2B2_0)*(B2B2_0))*1000 ;
D1B2B2_0 = ((D1B2_0)*(B2B2_0))*1000 ;
D2B2B2_0 = ((D2B2_0)*(B2B2_0))*1000 ;
D3B2B2_0 = ((D3B2_0)*(B2B2_0))*1000 ;
B1B2B2_0 = ((B1B2_0)*(B2B2_0))*1000 ;
B2B2B2_0 = ((B2B2_0)*(B2B2_0))*1000 ;

! indirect effects B2 group 1
NEW(A1B2A1_1
A2B2A1_1
D1B2A1_1
D2B2A1_1
D3B2A1_1
B1B2A1_1
B2B2A1_1
A1B2A2_1
A2B2A2_1
D1B2A2_1
D2B2A2_1
D3B2A2_1
B1B2A2_1
B2B2A2_1
A1B2D1_1
A2B2D1_1
D1B2D1_1
D2B2D1_1
D3B2D1_1
B1B2D1_1
B2B2D1_1
A1B2D2_1
A2B2D2_1
D1B2D2_1
D2B2D2_1
D3B2D2_1
B1B2D2_1
B2B2D2_1
A1B2D3_1
```

```
 A2B2D3_1
 D1B2D3_1
 D2B2D3_1
 D3B2D3_1
 B1B2D3_1
 B2B2D3_1
 A1B2B1_1
 A2B2B1_1
 D1B2B1_1
 D2B2B1_1
 D3B2B1_1
 B1B2B1_1
 B2B2B1_1
 A1B2B2_1
 A2B2B2_1
 D1B2B2_1
 D2B2B2_1
 D3B2B2_1
 B1B2B2_1
 B2B2B2_1);
A1B2A1_1 = ((A1B2_1)*(B2A1_1))*1000 ;
 A2B2A1_1 = ((A2B2_1)*(B2A1_1))*1000 ;
 D1B2A1_1 = ((D1B2_1)*(B2A1_1))*1000 ;
 D2B2A1_1 = ((D2B2_1)*(B2A1_1))*1000 ;
 D3B2A1_1 = ((D3B2_1)*(B2A1_1))*1000 ;
 B1B2A1_1 = ((B1B2_1)*(B2A1_1))*1000 ;
 B2B2A1_1 = ((B2B2_1)*(B2A1_1))*1000 ;
 A1B2A2_1 = ((A1B2_1)*(B2A2_1))*1000 ;
 A2B2A2_1 = ((A2B2_1)*(B2A2_1))*1000 ;
 D1B2A2_1 = ((D1B2_1)*(B2A2_1))*1000 ;
 D2B2A2_1 = ((D2B2_1)*(B2A2_1))*1000 ;
 D3B2A2_1 = ((D3B2_1)*(B2A2_1))*1000 ;
 B1B2A2_1 = ((B1B2_1)*(B2A2_1))*1000 ;
 B2B2A2_1 = ((B2B2_1)*(B2A2_1))*1000 ;
 A1B2D1_1 = ((A1B2_1)*(B2D1_1))*1000 ;
 A2B2D1_1 = ((A2B2_1)*(B2D1_1))*1000 ;
 D1B2D1_1 = ((D1B2_1)*(B2D1_1))*1000 ;
 D2B2D1_1 = ((D2B2_1)*(B2D1_1))*1000 ;
 D3B2D1_1 = ((D3B2_1)*(B2D1_1))*1000 ;
 B1B2D1_1 = ((B1B2_1)*(B2D1_1))*1000 ;
 B2B2D1_1 = ((B2B2_1)*(B2D1_1))*1000 ;
 A1B2D2_1 = ((A1B2_1)*(B2D2_1))*1000 ;
 A2B2D2_1 = ((A2B2_1)*(B2D2_1))*1000 ;
 D1B2D2_1 = ((D1B2_1)*(B2D2_1))*1000 ;
 D2B2D2_1 = ((D2B2_1)*(B2D2_1))*1000 ;
 D3B2D2_1 = ((D3B2_1)*(B2D2_1))*1000 ;
 B1B2D2_1 = ((B1B2_1)*(B2D2_1))*1000 ;
 B2B2D2_1 = ((B2B2_1)*(B2D2_1))*1000 ;
 A1B2D3_1 = ((A1B2_1)*(B2D3_1))*1000 ;
```

```
A2B2D3_1 = ((A2B2_1)*(B2D3_1))*1000 ;
D1B2D3_1 = ((D1B2_1)*(B2D3_1))*1000 ;
D2B2D3_1 = ((D2B2_1)*(B2D3_1))*1000 ;
D3B2D3_1 = ((D3B2_1)*(B2D3_1))*1000 ;
B1B2D3_1 = ((B1B2_1)*(B2D3_1))*1000 ;
B2B2D3_1 = ((B2B2_1)*(B2D3_1))*1000 ;
A1B2B1_1 = ((A1B2_1)*(B2B1_1))*1000 ;
A2B2B1_1 = ((A2B2_1)*(B2B1_1))*1000 ;
D1B2B1_1 = ((D1B2_1)*(B2B1_1))*1000 ;
D2B2B1_1 = ((D2B2_1)*(B2B1_1))*1000 ;
D3B2B1_1 = ((D3B2_1)*(B2B1_1))*1000 ;
B1B2B1_1 = ((B1B2_1)*(B2B1_1))*1000 ;
B2B2B1_1 = ((B2B2_1)*(B2B1_1))*1000 ;
A1B2B2_1 = ((A1B2_1)*(B2B2_1))*1000 ;
A2B2B2_1 = ((A2B2_1)*(B2B2_1))*1000 ;
D1B2B2_1 = ((D1B2_1)*(B2B2_1))*1000 ;
D2B2B2_1 = ((D2B2_1)*(B2B2_1))*1000 ;
D3B2B2_1 = ((D3B2_1)*(B2B2_1))*1000 ;
B1B2B2_1 = ((B1B2_1)*(B2B2_1))*1000 ;
B2B2B2_1 = ((B2B2_1)*(B2B2_1))*1000 ;

! indirect effects B2 group 2
NEW(A1B2A1_2
A2B2A1_2
D1B2A1_2
D2B2A1_2
D3B2A1_2
B1B2A1_2
B2B2A1_2
A1B2A2_2
A2B2A2_2
D1B2A2_2
D2B2A2_2
D3B2A2_2
B1B2A2_2
B2B2A2_2
A1B2D1_2
A2B2D1_2
D1B2D1_2
D2B2D1_2
D3B2D1_2
B1B2D1_2
B2B2D1_2
A1B2D2_2
A2B2D2_2
D1B2D2_2
D2B2D2_2
D3B2D2_2
B1B2D2_2
```

```
 B2B2D2_2
 A1B2D3_2
 A2B2D3_2
 D1B2D3_2
 D2B2D3_2
 D3B2D3_2
 B1B2D3_2
 B2B2D3_2
 A1B2B1_2
 A2B2B1_2
 D1B2B1_2
 D2B2B1_2
 D3B2B1_2
 B1B2B1_2
 B2B2B1_2
 A1B2B2_2
 A2B2B2_2
 D1B2B2_2
 D2B2B2_2
 D3B2B2_2
 B1B2B2_2
 B2B2B2_2);
A1B2A1_2 = ((A1B2_2)*(B2A1_2))*1000 ;
 A2B2A1_2 = ((A2B2_2)*(B2A1_2))*1000 ;
 D1B2A1_2 = ((D1B2_2)*(B2A1_2))*1000 ;
 D2B2A1_2 = ((D2B2_2)*(B2A1_2))*1000 ;
 D3B2A1_2 = ((D3B2_2)*(B2A1_2))*1000 ;
 B1B2A1_2 = ((B1B2_2)*(B2A1_2))*1000 ;
 B2B2A1_2 = ((B2B2_2)*(B2A1_2))*1000 ;
 A1B2A2_2 = ((A1B2_2)*(B2A2_2))*1000 ;
 A2B2A2_2 = ((A2B2_2)*(B2A2_2))*1000 ;
 D1B2A2_2 = ((D1B2_2)*(B2A2_2))*1000 ;
 D2B2A2_2 = ((D2B2_2)*(B2A2_2))*1000 ;
 D3B2A2_2 = ((D3B2_2)*(B2A2_2))*1000 ;
 B1B2A2_2 = ((B1B2_2)*(B2A2_2))*1000 ;
 B2B2A2_2 = ((B2B2_2)*(B2A2_2))*1000 ;
 A1B2D1_2 = ((A1B2_2)*(B2D1_2))*1000 ;
 A2B2D1_2 = ((A2B2_2)*(B2D1_2))*1000 ;
 D1B2D1_2 = ((D1B2_2)*(B2D1_2))*1000 ;
 D2B2D1_2 = ((D2B2_2)*(B2D1_2))*1000 ;
 D3B2D1_2 = ((D3B2_2)*(B2D1_2))*1000 ;
 B1B2D1_2 = ((B1B2_2)*(B2D1_2))*1000 ;
 B2B2D1_2 = ((B2B2_2)*(B2D1_2))*1000 ;
 A1B2D2_2 = ((A1B2_2)*(B2D2_2))*1000 ;
 A2B2D2_2 = ((A2B2_2)*(B2D2_2))*1000 ;
 D1B2D2_2 = ((D1B2_2)*(B2D2_2))*1000 ;
 D2B2D2_2 = ((D2B2_2)*(B2D2_2))*1000 ;
 D3B2D2_2 = ((D3B2_2)*(B2D2_2))*1000 ;
 B1B2D2_2 = ((B1B2_2)*(B2D2_2))*1000 ;
```

```
B2B2D2_2 = ((B2B2_2)*(B2D2_2))*1000 ;
A1B2D3_2 = ((A1B2_2)*(B2D3_2))*1000 ;
A2B2D3_2 = ((A2B2_2)*(B2D3_2))*1000 ;
D1B2D3_2 = ((D1B2_2)*(B2D3_2))*1000 ;
D2B2D3_2 = ((D2B2_2)*(B2D3_2))*1000 ;
D3B2D3_2 = ((D3B2_2)*(B2D3_2))*1000 ;
B1B2D3_2 = ((B1B2_2)*(B2D3_2))*1000 ;
B2B2D3_2 = ((B2B2_2)*(B2D3_2))*1000 ;
A1B2B1_2 = ((A1B2_2)*(B2B1_2))*1000 ;
A2B2B1_2 = ((A2B2_2)*(B2B1_2))*1000 ;
D1B2B1_2 = ((D1B2_2)*(B2B1_2))*1000 ;
D2B2B1_2 = ((D2B2_2)*(B2B1_2))*1000 ;
D3B2B1_2 = ((D3B2_2)*(B2B1_2))*1000 ;
B1B2B1_2 = ((B1B2_2)*(B2B1_2))*1000 ;
B2B2B1_2 = ((B2B2_2)*(B2B1_2))*1000 ;
A1B2B2_2 = ((A1B2_2)*(B2B2_2))*1000 ;
A2B2B2_2 = ((A2B2_2)*(B2B2_2))*1000 ;
D1B2B2_2 = ((D1B2_2)*(B2B2_2))*1000 ;
D2B2B2_2 = ((D2B2_2)*(B2B2_2))*1000 ;
D3B2B2_2 = ((D3B2_2)*(B2B2_2))*1000 ;
B1B2B2_2 = ((B1B2_2)*(B2B2_2))*1000 ;
B2B2B2_2 = ((B2B2_2)*(B2B2_2))*1000 ;


! Summaries of all indirect effects


! all indirect effects A1 group 0
NEW(aiA1_0);
aiA1_0 = A1A1A1_0 +
A2A1A1_0 +
D1A1A1_0 +
D2A1A1_0 +
D3A1A1_0 +
B1A1A1_0 +
B2A1A1_0 +
A1A1A2_0 +
A2A1A2_0 +
D1A1A2_0 +
D2A1A2_0 +
D3A1A2_0 +
B1A1A2_0 +
B2A1A2_0 +
A1A1D1_0 +
A2A1D1_0 +
D1A1D1_0 +
D2A1D1_0 +
D3A1D1_0 +
B1A1D1_0 +
```

```
B2A1D1_0 +
A1A1D2_0 +
A2A1D2_0 +
D1A1D2_0 +
D2A1D2_0 +
D3A1D2_0 +
B1A1D2_0 +
B2A1D2_0 +
A1A1D3_0 +
A2A1D3_0 +
D1A1D3_0 +
D2A1D3_0 +
D3A1D3_0 +
B1A1D3_0 +
B2A1D3_0 +
A1A1B1_0 +
A2A1B1_0 +
D1A1B1_0 +
D2A1B1_0 +
D3A1B1_0 +
B1A1B1_0 +
B2A1B1_0 +
A1A1B2_0 +
A2A1B2_0 +
D1A1B2_0 +
D2A1B2_0 +
D3A1B2_0 +
B1A1B2_0 +
B2A1B2_0;

! all indirect effects A1 group 1
NEW(aiA1_1);
aiA1_1 = A1A1A1_1 +
A2A1A1_1 +
D1A1A1_1 +
D2A1A1_1 +
D3A1A1_1 +
B1A1A1_1 +
B2A1A1_1 +
A1A1A2_1 +
A2A1A2_1 +
D1A1A2_1 +
D2A1A2_1 +
D3A1A2_1 +
B1A1A2_1 +
B2A1A2_1 +
A1A1D1_1 +
A2A1D1_1 +
D1A1D1_1 +
```

```
 D2A1D1_1 +
 D3A1D1_1 +
 B1A1D1_1 +
 B2A1D1_1 +
 A1A1D2_1 +
 A2A1D2_1 +
 D1A1D2_1 +
 D2A1D2_1 +
 D3A1D2_1 +
 B1A1D2_1 +
 B2A1D2_1 +
 A1A1D3_1 +
 A2A1D3_1 +
 D1A1D3_1 +
 D2A1D3_1 +
 D3A1D3_1 +
 B1A1D3_1 +
 B2A1D3_1 +
 A1A1B1_1 +
 A2A1B1_1 +
 D1A1B1_1 +
 D2A1B1_1 +
 D3A1B1_1 +
 B1A1B1_1 +
 B2A1B1_1 +
 A1A1B2_1 +
 A2A1B2_1 +
 D1A1B2_1 +
 D2A1B2_1 +
 D3A1B2_1 +
 B1A1B2_1 +
 B2A1B2_1;

 ! all indirect effects A1 group 2
 NEW(aiA1_2);
aiA1_2 = A1A1A1_2 +
 A2A1A1_2 +
 D1A1A1_2 +
 D2A1A1_2 +
 D3A1A1_2 +
 B1A1A1_2 +
 B2A1A1_2 +
 A1A1A2_2 +
 A2A1A2_2 +
 D1A1A2_2 +
 D2A1A2_2 +
 D3A1A2_2 +
 B1A1A2_2 +
 B2A1A2_2 +
```

```
A1A1D1_2 +
A2A1D1_2 +
D1A1D1_2 +
D2A1D1_2 +
D3A1D1_2 +
B1A1D1_2 +
B2A1D1_2 +
A1A1D2_2 +
A2A1D2_2 +
D1A1D2_2 +
D2A1D2_2 +
D3A1D2_2 +
B1A1D2_2 +
B2A1D2_2 +
A1A1D3_2 +
A2A1D3_2 +
D1A1D3_2 +
D2A1D3_2 +
D3A1D3_2 +
B1A1D3_2 +
B2A1D3_2 +
A1A1B1_2 +
A2A1B1_2 +
D1A1B1_2 +
D2A1B1_2 +
D3A1B1_2 +
B1A1B1_2 +
B2A1B1_2 +
A1A1B2_2 +
A2A1B2_2 +
D1A1B2_2 +
D2A1B2_2 +
D3A1B2_2 +
B1A1B2_2 +
B2A1B2_2;

! all indirect effects A2 group 0
NEW(aiA2_0);
aiA2_0 = A1A2A1_0 +
A2A2A1_0 +
D1A2A1_0 +
D2A2A1_0 +
D3A2A1_0 +
B1A2A1_0 +
B2A2A1_0 +
A1A2A2_0 +
A2A2A2_0 +
D1A2A2_0 +
D2A2A2_0 +
```

```
D3A2A2_0 +
B1A2A2_0 +
B2A2A2_0 +
A1A2D1_0 +
A2A2D1_0 +
D1A2D1_0 +
D2A2D1_0 +
D3A2D1_0 +
B1A2D1_0 +
B2A2D1_0 +
A1A2D2_0 +
A2A2D2_0 +
D1A2D2_0 +
D2A2D2_0 +
D3A2D2_0 +
B1A2D2_0 +
B2A2D2_0 +
A1A2D3_0 +
A2A2D3_0 +
D1A2D3_0 +
D2A2D3_0 +
D3A2D3_0 +
B1A2D3_0 +
B2A2D3_0 +
A1A2B1_0 +
A2A2B1_0 +
D1A2B1_0 +
D2A2B1_0 +
D3A2B1_0 +
B1A2B1_0 +
B2A2B1_0 +
A1A2B2_0 +
A2A2B2_0 +
D1A2B2_0 +
D2A2B2_0 +
D3A2B2_0 +
B1A2B2_0 +
B2A2B2_0;

! all indirect effects A2 group 1
NEW(aiA2_1);
aiA2_1 = A1A2A1_1 +
A2A2A1_1 +
D1A2A1_1 +
D2A2A1_1 +
D3A2A1_1 +
B1A2A1_1 +
B2A2A1_1 +
A1A2A2_1 +
```

```
A2A2A2_1 +
D1A2A2_1 +
D2A2A2_1 +
D3A2A2_1 +
B1A2A2_1 +
B2A2A2_1 +
A1A2D1_1 +
A2A2D1_1 +
D1A2D1_1 +
D2A2D1_1 +
D3A2D1_1 +
B1A2D1_1 +
B2A2D1_1 +
A1A2D2_1 +
A2A2D2_1 +
D1A2D2_1 +
D2A2D2_1 +
D3A2D2_1 +
B1A2D2_1 +
B2A2D2_1 +
A1A2D3_1 +
A2A2D3_1 +
D1A2D3_1 +
D2A2D3_1 +
D3A2D3_1 +
B1A2D3_1 +
B2A2D3_1 +
A1A2B1_1 +
A2A2B1_1 +
D1A2B1_1 +
D2A2B1_1 +
D3A2B1_1 +
B1A2B1_1 +
B2A2B1_1 +
A1A2B2_1 +
A2A2B2_1 +
D1A2B2_1 +
D2A2B2_1 +
D3A2B2_1 +
B1A2B2_1 +
B2A2B2_1;

! all indirect effects A2 group 2
NEW(aiA2_2);
aiA2_2 = A1A2A1_2 +
A2A2A1_2 +
D1A2A1_2 +
D2A2A1_2 +
D3A2A1_2 +
```

B1A2A1_2 +
B2A2A1_2 +
A1A2A2_2 +
A2A2A2_2 +
D1A2A2_2 +
D2A2A2_2 +
D3A2A2_2 +
B1A2A2_2 +
B2A2A2_2 +
A1A2D1_2 +
A2A2D1_2 +
D1A2D1_2 +
D2A2D1_2 +
D3A2D1_2 +
B1A2D1_2 +
B2A2D1_2 +
A1A2D2_2 +
A2A2D2_2 +
D1A2D2_2 +
D2A2D2_2 +
D3A2D2_2 +
B1A2D2_2 +
B2A2D2_2 +
A1A2D3_2 +
A2A2D3_2 +
D1A2D3_2 +
D2A2D3_2 +
D3A2D3_2 +
B1A2D3_2 +
B2A2D3_2 +
A1A2B1_2 +
A2A2B1_2 +
D1A2B1_2 +
D2A2B1_2 +
D3A2B1_2 +
B1A2B1_2 +
B2A2B1_2 +
A1A2B2_2 +
A2A2B2_2 +
D1A2B2_2 +
D2A2B2_2 +
D3A2B2_2 +
B1A2B2_2 +
B2A2B2_2;

! all indirect effects D1 group 0
NEW(aiD1_0);
aiD1_0 = A1D1A1_0 +
A2D1A1_0 +

D1D1A1_0 +
D2D1A1_0 +
D3D1A1_0 +
B1D1A1_0 +
B2D1A1_0 +
A1D1A2_0 +
A2D1A2_0 +
D1D1A2_0 +
D2D1A2_0 +
D3D1A2_0 +
B1D1A2_0 +
B2D1A2_0 +
A1D1D1_0 +
A2D1D1_0 +
D1D1D1_0 +
D2D1D1_0 +
D3D1D1_0 +
B1D1D1_0 +
B2D1D1_0 +
A1D1D2_0 +
A2D1D2_0 +
D1D1D2_0 +
D2D1D2_0 +
D3D1D2_0 +
B1D1D2_0 +
B2D1D2_0 +
A1D1D3_0 +
A2D1D3_0 +
D1D1D3_0 +
D2D1D3_0 +
D3D1D3_0 +
B1D1D3_0 +
B2D1D3_0 +
A1D1B1_0 +
A2D1B1_0 +
D1D1B1_0 +
D2D1B1_0 +
D3D1B1_0 +
B1D1B1_0 +
B2D1B1_0 +
A1D1B2_0 +
A2D1B2_0 +
D1D1B2_0 +
D2D1B2_0 +
D3D1B2_0 +
B1D1B2_0 +
B2D1B2_0;

! all indirect effects D1 group 1

```
 NEW(aiD1_1);
aiD1_1 = A1D1A1_1 +
 A2D1A1_1 +
 D1D1A1_1 +
 D2D1A1_1 +
 D3D1A1_1 +
 B1D1A1_1 +
 B2D1A1_1 +
 A1D1A2_1 +
 A2D1A2_1 +
 D1D1A2_1 +
 D2D1A2_1 +
 D3D1A2_1 +
 B1D1A2_1 +
 B2D1A2_1 +
 A1D1D1_1 +
 A2D1D1_1 +
 D1D1D1_1 +
 D2D1D1_1 +
 D3D1D1_1 +
 B1D1D1_1 +
 B2D1D1_1 +
 A1D1D2_1 +
 A2D1D2_1 +
 D1D1D2_1 +
 D2D1D2_1 +
 D3D1D2_1 +
 B1D1D2_1 +
 B2D1D2_1 +
 A1D1D3_1 +
 A2D1D3_1 +
 D1D1D3_1 +
 D2D1D3_1 +
 D3D1D3_1 +
 B1D1D3_1 +
 B2D1D3_1 +
 A1D1B1_1 +
 A2D1B1_1 +
 D1D1B1_1 +
 D2D1B1_1 +
 D3D1B1_1 +
 B1D1B1_1 +
 B2D1B1_1 +
 A1D1B2_1 +
 A2D1B2_1 +
 D1D1B2_1 +
 D2D1B2_1 +
 D3D1B2_1 +
 B1D1B2_1 +
```

```
 B2D1B2_1;

 ! all indirect effects D1 group 2
 NEW(aiD1_2);
aiD1_2 = A1D1A1_2 +
 A2D1A1_2 +
 D1D1A1_2 +
 D2D1A1_2 +
 D3D1A1_2 +
 B1D1A1_2 +
 B2D1A1_2 +
 A1D1A2_2 +
 A2D1A2_2 +
 D1D1A2_2 +
 D2D1A2_2 +
 D3D1A2_2 +
 B1D1A2_2 +
 B2D1A2_2 +
 A1D1D1_2 +
 A2D1D1_2 +
 D1D1D1_2 +
 D2D1D1_2 +
 D3D1D1_2 +
 B1D1D1_2 +
 B2D1D1_2 +
 A1D1D2_2 +
 A2D1D2_2 +
 D1D1D2_2 +
 D2D1D2_2 +
 D3D1D2_2 +
 B1D1D2_2 +
 B2D1D2_2 +
 A1D1D3_2 +
 A2D1D3_2 +
 D1D1D3_2 +
 D2D1D3_2 +
 D3D1D3_2 +
 B1D1D3_2 +
 B2D1D3_2 +
 A1D1B1_2 +
 A2D1B1_2 +
 D1D1B1_2 +
 D2D1B1_2 +
 D3D1B1_2 +
 B1D1B1_2 +
 B2D1B1_2 +
 A1D1B2_2 +
 A2D1B2_2 +
 D1D1B2_2 +
```

```
 D2D1B2_2 +
 D3D1B2_2 +
 B1D1B2_2 +
 B2D1B2_2;

 ! all indirect effects D2 group 0
 NEW(aiD2_0);
aiD2_0 = A1D2A1_0 +
 A2D2A1_0 +
 D1D2A1_0 +
 D2D2A1_0 +
 D3D2A1_0 +
 B1D2A1_0 +
 B2D2A1_0 +
 A1D2A2_0 +
 A2D2A2_0 +
 D1D2A2_0 +
 D2D2A2_0 +
 D3D2A2_0 +
 B1D2A2_0 +
 B2D2A2_0 +
 A1D2D1_0 +
 A2D2D1_0 +
 D1D2D1_0 +
 D2D2D1_0 +
 D3D2D1_0 +
 B1D2D1_0 +
 B2D2D1_0 +
 A1D2D2_0 +
 A2D2D2_0 +
 D1D2D2_0 +
 D2D2D2_0 +
 D3D2D2_0 +
 B1D2D2_0 +
 B2D2D2_0 +
 A1D2D3_0 +
 A2D2D3_0 +
 D1D2D3_0 +
 D2D2D3_0 +
 D3D2D3_0 +
 B1D2D3_0 +
 B2D2D3_0 +
 A1D2B1_0 +
 A2D2B1_0 +
 D1D2B1_0 +
 D2D2B1_0 +
 D3D2B1_0 +
 B1D2B1_0 +
 B2D2B1_0 +
```

```
 A1D2B2_0 +
 A2D2B2_0 +
 D1D2B2_0 +
 D2D2B2_0 +
 D3D2B2_0 +
 B1D2B2_0 +
 B2D2B2_0;

 ! all indirect effects D2 group 1
 NEW(aiD2_1);
aiD2_1 = A1D2A1_1 +
 A2D2A1_1 +
 D1D2A1_1 +
 D2D2A1_1 +
 D3D2A1_1 +
 B1D2A1_1 +
 B2D2A1_1 +
 A1D2A2_1 +
 A2D2A2_1 +
 D1D2A2_1 +
 D2D2A2_1 +
 D3D2A2_1 +
 B1D2A2_1 +
 B2D2A2_1 +
 A1D2D1_1 +
 A2D2D1_1 +
 D1D2D1_1 +
 D2D2D1_1 +
 D3D2D1_1 +
 B1D2D1_1 +
 B2D2D1_1 +
 A1D2D2_1 +
 A2D2D2_1 +
 D1D2D2_1 +
 D2D2D2_1 +
 D3D2D2_1 +
 B1D2D2_1 +
 B2D2D2_1 +
 A1D2D3_1 +
 A2D2D3_1 +
 D1D2D3_1 +
 D2D2D3_1 +
 D3D2D3_1 +
 B1D2D3_1 +
 B2D2D3_1 +
 A1D2B1_1 +
 A2D2B1_1 +
 D1D2B1_1 +
 D2D2B1_1 +
```

```
 D3D2B1_1 +
 B1D2B1_1 +
 B2D2B1_1 +
 A1D2B2_1 +
 A2D2B2_1 +
 D1D2B2_1 +
 D2D2B2_1 +
 D3D2B2_1 +
 B1D2B2_1 +
 B2D2B2_1;

 ! all indirect effects D2 group 2
 NEW(aiD2_2);
aiD2_2 = A1D2A1_2 +
 A2D2A1_2 +
 D1D2A1_2 +
 D2D2A1_2 +
 D3D2A1_2 +
 B1D2A1_2 +
 B2D2A1_2 +
 A1D2A2_2 +
 A2D2A2_2 +
 D1D2A2_2 +
 D2D2A2_2 +
 D3D2A2_2 +
 B1D2A2_2 +
 B2D2A2_2 +
 A1D2D1_2 +
 A2D2D1_2 +
 D1D2D1_2 +
 D2D2D1_2 +
 D3D2D1_2 +
 B1D2D1_2 +
 B2D2D1_2 +
 A1D2D2_2 +
 A2D2D2_2 +
 D1D2D2_2 +
 D2D2D2_2 +
 D3D2D2_2 +
 B1D2D2_2 +
 B2D2D2_2 +
 A1D2D3_2 +
 A2D2D3_2 +
 D1D2D3_2 +
 D2D2D3_2 +
 D3D2D3_2 +
 B1D2D3_2 +
 B2D2D3_2 +
 A1D2B1_2 +
```

```
A2D2B1_2 +
D1D2B1_2 +
D2D2B1_2 +
D3D2B1_2 +
B1D2B1_2 +
B2D2B1_2 +
A1D2B2_2 +
A2D2B2_2 +
D1D2B2_2 +
D2D2B2_2 +
D3D2B2_2 +
B1D2B2_2 +
B2D2B2_2;

! all indirect effects D3 group 0
NEW(aiD3_0);
aiD3_0 = A1D3A1_0 +
A2D3A1_0 +
D1D3A1_0 +
D2D3A1_0 +
D3D3A1_0 +
B1D3A1_0 +
B2D3A1_0 +
A1D3A2_0 +
A2D3A2_0 +
D1D3A2_0 +
D2D3A2_0 +
D3D3A2_0 +
B1D3A2_0 +
B2D3A2_0 +
A1D3D1_0 +
A2D3D1_0 +
D1D3D1_0 +
D2D3D1_0 +
D3D3D1_0 +
B1D3D1_0 +
B2D3D1_0 +
A1D3D2_0 +
A2D3D2_0 +
D1D3D2_0 +
D2D3D2_0 +
D3D3D2_0 +
B1D3D2_0 +
B2D3D2_0 +
A1D3D3_0 +
A2D3D3_0 +
D1D3D3_0 +
D2D3D3_0 +
D3D3D3_0 +
```

```
 B1D3D3_0 +
 B2D3D3_0 +
 A1D3B1_0 +
 A2D3B1_0 +
 D1D3B1_0 +
 D2D3B1_0 +
 D3D3B1_0 +
 B1D3B1_0 +
 B2D3B1_0 +
 A1D3B2_0 +
 A2D3B2_0 +
 D1D3B2_0 +
 D2D3B2_0 +
 D3D3B2_0 +
 B1D3B2_0 +
 B2D3B2_0;

 ! all indirect effects D3 group 1
 NEW(aiD3_1);
aiD3_1 = A1D3A1_1 +
 A2D3A1_1 +
 D1D3A1_1 +
 D2D3A1_1 +
 D3D3A1_1 +
 B1D3A1_1 +
 B2D3A1_1 +
 A1D3A2_1 +
 A2D3A2_1 +
 D1D3A2_1 +
 D2D3A2_1 +
 D3D3A2_1 +
 B1D3A2_1 +
 B2D3A2_1 +
 A1D3D1_1 +
 A2D3D1_1 +
 D1D3D1_1 +
 D2D3D1_1 +
 D3D3D1_1 +
 B1D3D1_1 +
 B2D3D1_1 +
 A1D3D2_1 +
 A2D3D2_1 +
 D1D3D2_1 +
 D2D3D2_1 +
 D3D3D2_1 +
 B1D3D2_1 +
 B2D3D2_1 +
 A1D3D3_1 +
 A2D3D3_1 +
```

```
 D1D3D3_1 +
 D2D3D3_1 +
 D3D3D3_1 +
 B1D3D3_1 +
 B2D3D3_1 +
 A1D3B1_1 +
 A2D3B1_1 +
 D1D3B1_1 +
 D2D3B1_1 +
 D3D3B1_1 +
 B1D3B1_1 +
 B2D3B1_1 +
 A1D3B2_1 +
 A2D3B2_1 +
 D1D3B2_1 +
 D2D3B2_1 +
 D3D3B2_1 +
 B1D3B2_1 +
 B2D3B2_1;

 ! all indirect effects D3 group 2
 NEW(aiD3_2);
aiD3_2 = A1D3A1_2 +
 A2D3A1_2 +
 D1D3A1_2 +
 D2D3A1_2 +
 D3D3A1_2 +
 B1D3A1_2 +
 B2D3A1_2 +
 A1D3A2_2 +
 A2D3A2_2 +
 D1D3A2_2 +
 D2D3A2_2 +
 D3D3A2_2 +
 B1D3A2_2 +
 B2D3A2_2 +
 A1D3D1_2 +
 A2D3D1_2 +
 D1D3D1_2 +
 D2D3D1_2 +
 D3D3D1_2 +
 B1D3D1_2 +
 B2D3D1_2 +
 A1D3D2_2 +
 A2D3D2_2 +
 D1D3D2_2 +
 D2D3D2_2 +
 D3D3D2_2 +
 B1D3D2_2 +
```

```
B2D3D2_2 +
A1D3D3_2 +
A2D3D3_2 +
D1D3D3_2 +
D2D3D3_2 +
D3D3D3_2 +
B1D3D3_2 +
B2D3D3_2 +
A1D3B1_2 +
A2D3B1_2 +
D1D3B1_2 +
D2D3B1_2 +
D3D3B1_2 +
B1D3B1_2 +
B2D3B1_2 +
A1D3B2_2 +
A2D3B2_2 +
D1D3B2_2 +
D2D3B2_2 +
D3D3B2_2 +
B1D3B2_2 +
B2D3B2_2;

! all indirect effects B1 group 0
NEW(aiB1_0);
aiB1_0 = A1B1A1_0 +
A2B1A1_0 +
D1B1A1_0 +
D2B1A1_0 +
D3B1A1_0 +
B1B1A1_0 +
B2B1A1_0 +
A1B1A2_0 +
A2B1A2_0 +
D1B1A2_0 +
D2B1A2_0 +
D3B1A2_0 +
B1B1A2_0 +
B2B1A2_0 +
A1B1D1_0 +
A2B1D1_0 +
D1B1D1_0 +
D2B1D1_0 +
D3B1D1_0 +
B1B1D1_0 +
B2B1D1_0 +
A1B1D2_0 +
A2B1D2_0 +
D1B1D2_0 +
```

```
 D2B1D2_0 +
 D3B1D2_0 +
 B1B1D2_0 +
 B2B1D2_0 +
 A1B1D3_0 +
 A2B1D3_0 +
 D1B1D3_0 +
 D2B1D3_0 +
 D3B1D3_0 +
 B1B1D3_0 +
 B2B1D3_0 +
 A1B1B1_0 +
 A2B1B1_0 +
 D1B1B1_0 +
 D2B1B1_0 +
 D3B1B1_0 +
 B1B1B1_0 +
 B2B1B1_0 +
 A1B1B2_0 +
 A2B1B2_0 +
 D1B1B2_0 +
 D2B1B2_0 +
 D3B1B2_0 +
 B1B1B2_0 +
 B2B1B2_0;

 ! all indirect effects B1 group 1
 NEW(aiB1_1);
aiB1_1 = A1B1A1_1 +
 A2B1A1_1 +
 D1B1A1_1 +
 D2B1A1_1 +
 D3B1A1_1 +
 B1B1A1_1 +
 B2B1A1_1 +
 A1B1A2_1 +
 A2B1A2_1 +
 D1B1A2_1 +
 D2B1A2_1 +
 D3B1A2_1 +
 B1B1A2_1 +
 B2B1A2_1 +
 A1B1D1_1 +
 A2B1D1_1 +
 D1B1D1_1 +
 D2B1D1_1 +
 D3B1D1_1 +
 B1B1D1_1 +
 B2B1D1_1 +
```

```
 A1B1D2_1 +
 A2B1D2_1 +
 D1B1D2_1 +
 D2B1D2_1 +
 D3B1D2_1 +
 B1B1D2_1 +
 B2B1D2_1 +
 A1B1D3_1 +
 A2B1D3_1 +
 D1B1D3_1 +
 D2B1D3_1 +
 D3B1D3_1 +
 B1B1D3_1 +
 B2B1D3_1 +
 A1B1B1_1 +
 A2B1B1_1 +
 D1B1B1_1 +
 D2B1B1_1 +
 D3B1B1_1 +
 B1B1B1_1 +
 B2B1B1_1 +
 A1B1B2_1 +
 A2B1B2_1 +
 D1B1B2_1 +
 D2B1B2_1 +
 D3B1B2_1 +
 B1B1B2_1 +
 B2B1B2_1;

 ! all indirect effects B1 group 2
 NEW(aiB1_2);
aiB1_2 = A1B1A1_2 +
 A2B1A1_2 +
 D1B1A1_2 +
 D2B1A1_2 +
 D3B1A1_2 +
 B1B1A1_2 +
 B2B1A1_2 +
 A1B1A2_2 +
 A2B1A2_2 +
 D1B1A2_2 +
 D2B1A2_2 +
 D3B1A2_2 +
 B1B1A2_2 +
 B2B1A2_2 +
 A1B1D1_2 +
 A2B1D1_2 +
 D1B1D1_2 +
 D2B1D1_2 +
```

```
 D3B1D1_2 +
 B1B1D1_2 +
 B2B1D1_2 +
 A1B1D2_2 +
 A2B1D2_2 +
 D1B1D2_2 +
 D2B1D2_2 +
 D3B1D2_2 +
 B1B1D2_2 +
 B2B1D2_2 +
 A1B1D3_2 +
 A2B1D3_2 +
 D1B1D3_2 +
 D2B1D3_2 +
 D3B1D3_2 +
 B1B1D3_2 +
 B2B1D3_2 +
 A1B1B1_2 +
 A2B1B1_2 +
 D1B1B1_2 +
 D2B1B1_2 +
 D3B1B1_2 +
 B1B1B1_2 +
 B2B1B1_2 +
 A1B1B2_2 +
 A2B1B2_2 +
 D1B1B2_2 +
 D2B1B2_2 +
 D3B1B2_2 +
 B1B1B2_2 +
 B2B1B2_2;

 ! all indirect effects B2 group 0
 NEW(aiB2_0);
aiB2_0 = A1B2A1_0 +
 A2B2A1_0 +
 D1B2A1_0 +
 D2B2A1_0 +
 D3B2A1_0 +
 B1B2A1_0 +
 B2B2A1_0 +
 A1B2A2_0 +
 A2B2A2_0 +
 D1B2A2_0 +
 D2B2A2_0 +
 D3B2A2_0 +
 B1B2A2_0 +
 B2B2A2_0 +
 A1B2D1_0 +
```

```
A2B2D1_0 +
D1B2D1_0 +
D2B2D1_0 +
D3B2D1_0 +
B1B2D1_0 +
B2B2D1_0 +
A1B2D2_0 +
A2B2D2_0 +
D1B2D2_0 +
D2B2D2_0 +
D3B2D2_0 +
B1B2D2_0 +
B2B2D2_0 +
A1B2D3_0 +
A2B2D3_0 +
D1B2D3_0 +
D2B2D3_0 +
D3B2D3_0 +
B1B2D3_0 +
B2B2D3_0 +
A1B2B1_0 +
A2B2B1_0 +
D1B2B1_0 +
D2B2B1_0 +
D3B2B1_0 +
B1B2B1_0 +
B2B2B1_0 +
A1B2B2_0 +
A2B2B2_0 +
D1B2B2_0 +
D2B2B2_0 +
D3B2B2_0 +
B1B2B2_0 +
B2B2B2_0;

! all indirect effects B2 group 1
NEW(aiB2_1);
aiB2_1 = A1B2A1_1 +
A2B2A1_1 +
D1B2A1_1 +
D2B2A1_1 +
D3B2A1_1 +
B1B2A1_1 +
B2B2A1_1 +
A1B2A2_1 +
A2B2A2_1 +
D1B2A2_1 +
D2B2A2_1 +
D3B2A2_1 +
```

```
B1B2A2_1 +
B2B2A2_1 +
A1B2D1_1 +
A2B2D1_1 +
D1B2D1_1 +
D2B2D1_1 +
D3B2D1_1 +
B1B2D1_1 +
B2B2D1_1 +
A1B2D2_1 +
A2B2D2_1 +
D1B2D2_1 +
D2B2D2_1 +
D3B2D2_1 +
B1B2D2_1 +
B2B2D2_1 +
A1B2D3_1 +
A2B2D3_1 +
D1B2D3_1 +
D2B2D3_1 +
D3B2D3_1 +
B1B2D3_1 +
B2B2D3_1 +
A1B2B1_1 +
A2B2B1_1 +
D1B2B1_1 +
D2B2B1_1 +
D3B2B1_1 +
B1B2B1_1 +
B2B2B1_1 +
A1B2B2_1 +
A2B2B2_1 +
D1B2B2_1 +
D2B2B2_1 +
D3B2B2_1 +
B1B2B2_1 +
B2B2B2_1;

! all indirect effects B2 group 2
NEW(aiB2_2);
aiB2_2 = A1B2A1_2 +
A2B2A1_2 +
D1B2A1_2 +
D2B2A1_2 +
D3B2A1_2 +
B1B2A1_2 +
B2B2A1_2 +
A1B2A2_2 +
A2B2A2_2 +
```

```
 D1B2A2_2 +
 D2B2A2_2 +
 D3B2A2_2 +
 B1B2A2_2 +
 B2B2A2_2 +
 A1B2D1_2 +
 A2B2D1_2 +
 D1B2D1_2 +
 D2B2D1_2 +
 D3B2D1_2 +
 B1B2D1_2 +
 B2B2D1_2 +
 A1B2D2_2 +
 A2B2D2_2 +
 D1B2D2_2 +
 D2B2D2_2 +
 D3B2D2_2 +
 B1B2D2_2 +
 B2B2D2_2 +
 A1B2D3_2 +
 A2B2D3_2 +
 D1B2D3_2 +
 D2B2D3_2 +
 D3B2D3_2 +
 B1B2D3_2 +
 B2B2D3_2 +
 A1B2B1_2 +
 A2B2B1_2 +
 D1B2B1_2 +
 D2B2B1_2 +
 D3B2B1_2 +
 B1B2B1_2 +
 B2B2B1_2 +
 A1B2B2_2 +
 A2B2B2_2 +
 D1B2B2_2 +
 D2B2B2_2 +
 D3B2B2_2 +
 B1B2B2_2 +
 B2B2B2_2;

 ! Generalized bridge centrality


 ! r-centrality  A1 Group 0

NEW(rcA1_0  rcA1_0s);
rcA1_0 = D1A1A2_0 +
 D2A1A2_0 +
```

```
 D3A1A2_0 +
 B1A1A2_0 +
 B2A1A2_0 +
 A2A1D1_0 +
 A2A1D2_0 +
 A2A1D3_0 +
 A2A1B1_0 +
 A2A1B2_0 +
 D1A1B1_0 +
 D2A1B1_0 +
 D3A1B1_0 +
 D1A1B2_0 +
 D2A1B2_0 +
 D3A1B2_0 +
 B1A1D1_0 +
 B2A1D1_0 +
 B1A1D2_0 +
 B2A1D2_0 +
 B1A1D3_0 +
 B2A1D3_0 +
 B2A1B1_0 +
 B1A1B2_0;

 ! Re-scaled r-centrality
rcA1_0s = (rcA1_0)/24;


 ! r-centrality  A2 Group 0

NEW(rcA2_0  rcA2_0s);
rcA2_0 = D1A2A1_0 +
 D2A2A1_0 +
 D3A2A1_0 +
 B1A2A1_0 +
 B2A2A1_0 +
 A1A2D1_0 +
 A1A2D2_0 +
 A1A2D3_0 +
 A1A2B1_0 +
 A1A2B2_0 +
 D1A2B1_0 +
 D2A2B1_0 +
 D3A2B1_0 +
 D1A2B2_0 +
 D2A2B2_0 +
 D3A2B2_0 +
 B1A2D1_0 +
 B2A2D1_0 +
 B1A2D2_0 +
```

```
 B2A2D2_0 +
 B1A2D3_0 +
 B2A2D3_0 +
 B2A2B1_0 +
 B1A2B2_0;

 ! Re-scaled r-centrality
rcA2_0s = (rcA2_0)/24;


 ! r-centrality  D1 Group 0

NEW(rcD1_0  rcD1_0s);
rcD1_0 = A1D1D2_0 +
 A2D1D2_0 +
 B1D1D2_0 +
 B2D1D2_0 +
 A1D1D3_0 +
 A2D1D3_0 +
 B1D1D3_0 +
 B2D1D3_0 +
 D2D1A1_0 +
 D3D1A1_0 +
 D2D1A2_0 +
 D3D1A2_0 +
 D2D1B1_0 +
 D3D1B1_0 +
 D2D1B2_0 +
 D3D1B2_0 +
 A1D1B1_0 +
 A2D1B1_0 +
 A1D1B2_0 +
 A2D1B2_0 +
 B1D1A1_0 +
 B2D1A1_0 +
 B1D1A2_0 +
 B2D1A2_0 +
 B2D1B1_0 +
 B1D1B2_0;

 ! Re-scaled r-centrality
rcD1_0s = (rcD1_0)/26;


 ! r-centrality  D2 Group 0

NEW(rcD2_0  rcD2_0s);
rcD2_0 = A1D2D1_0 +
 A2D2D1_0 +
```

```
 B1D2D1_0 +
 B2D2D1_0 +
 A1D2D3_0 +
 A2D2D3_0 +
 B1D2D3_0 +
 B2D2D3_0 +
 D1D2A1_0 +
 D3D2A1_0 +
 D1D2A2_0 +
 D3D2A2_0 +
 D1D2B1_0 +
 D3D2B1_0 +
 D1D2B2_0 +
 D3D2B2_0 +
 A1D2B1_0 +
 A2D2B1_0 +
 A1D2B2_0 +
 A2D2B2_0 +
 B1D2A1_0 +
 B2D2A1_0 +
 B1D2A2_0 +
 B2D2A2_0 +
 B2D2B1_0 +
 B1D2B2_0;

 ! Re-scaled r-centrality
rcD2_0s = (rcD2_0)/26;


 ! r-centrality  D3 Group 0

NEW(rcD3_0  rcD3_0s);
rcD3_0 = A1D3D1_0 +
 A2D3D1_0 +
 B1D3D1_0 +
 B2D3D1_0 +
 A1D3D2_0 +
 A2D3D2_0 +
 B1D3D2_0 +
 B2D3D2_0 +
 D1D3A1_0 +
 D2D3A1_0 +
 D1D3A2_0 +
 D2D3A2_0 +
 D1D3B1_0 +
 D2D3B1_0 +
 D1D3B2_0 +
 D2D3B2_0 +
 A1D3B1_0 +
```

```
 A2D3B1_0 +
 A1D3B2_0 +
 A2D3B2_0 +
 B1D3A1_0 +
 B2D3A1_0 +
 B1D3A2_0 +
 B2D3A2_0 +
 B2D3B1_0 +
 B1D3B2_0;

 ! Re-scaled r-centrality
rcD3_0s = (rcD3_0)/26;


 ! r-centrality  B1 Group 0

NEW(rcB1_0  rcB1_0s);
rcB1_0 = D1B1A1_0 +
 D2B1A1_0 +
 D3B1A1_0 +
 B2B1A1_0 +
 D1B1A2_0 +
 D2B1A2_0 +
 D3B1A2_0 +
 B2B1A2_0 +
 A1B1D1_0 +
 A2B1D1_0 +
 B2B1D1_0 +
 A1B1D2_0 +
 A2B1D2_0 +
 B2B1D2_0 +
 A1B1D3_0 +
 A2B1D3_0 +
 B2B1D3_0 +
 A1B1B2_0 +
 A2B1B2_0 +
 D1B1B2_0 +
 D2B1B2_0 +
 D3B1B2_0;

 ! Re-scaled r-centrality
rcB1_0s = (rcB1_0)/22;


 ! r-centrality  B2 Group 0

NEW(rcB2_0  rcB2_0s);
rcB2_0 = D1B2A1_0 +
 D2B2A1_0 +
```

```
 D3B2A1_0 +
 B1B2A1_0 +
 D1B2A2_0 +
 D2B2A2_0 +
 D3B2A2_0 +
 B1B2A2_0 +
 A1B2D1_0 +
 A2B2D1_0 +
 B1B2D1_0 +
 A1B2D2_0 +
 A2B2D2_0 +
 B1B2D2_0 +
 A1B2D3_0 +
 A2B2D3_0 +
 B1B2D3_0 +
 A1B2B1_0 +
 A2B2B1_0 +
 D1B2B1_0 +
 D2B2B1_0 +
 D3B2B1_0;

 ! Re-scaled r-centrality
rcB2_0s = (rcB2_0)/22;


 ! r-centrality  A1 Group 1

NEW(rcA1_1  rcA1_1s);
rcA1_1 = D1A1A2_1 +
 D2A1A2_1 +
 D3A1A2_1 +
 B1A1A2_1 +
 B2A1A2_1 +
 A2A1D1_1 +
 A2A1D2_1 +
 A2A1D3_1 +
 A2A1B1_1 +
 A2A1B2_1 +
 D1A1B1_1 +
 D2A1B1_1 +
 D3A1B1_1 +
 D1A1B2_1 +
 D2A1B2_1 +
 D3A1B2_1 +
 B1A1D1_1 +
 B2A1D1_1 +
 B1A1D2_1 +
 B2A1D2_1 +
 B1A1D3_1 +
```

```
 B2A1D3_1 +
 B2A1B1_1 +
 B1A1B2_1;

 ! Re-scaled r-centrality
rcA1_1s = (rcA1_1)/24;


 ! r-centrality  A2 Group 1

NEW(rcA2_1  rcA2_1s);
rcA2_1 = D1A2A1_1 +
 D2A2A1_1 +
 D3A2A1_1 +
 B1A2A1_1 +
 B2A2A1_1 +
 A1A2D1_1 +
 A1A2D2_1 +
 A1A2D3_1 +
 A1A2B1_1 +
 A1A2B2_1 +
 D1A2B1_1 +
 D2A2B1_1 +
 D3A2B1_1 +
 D1A2B2_1 +
 D2A2B2_1 +
 D3A2B2_1 +
 B1A2D1_1 +
 B2A2D1_1 +
 B1A2D2_1 +
 B2A2D2_1 +
 B1A2D3_1 +
 B2A2D3_1 +
 B2A2B1_1 +
 B1A2B2_1;

 ! Re-scaled r-centrality
rcA2_1s = (rcA2_1)/24;


 ! r-centrality  D1 Group 1

NEW(rcD1_1  rcD1_1s);
rcD1_1 = A1D1D2_1 +
 A2D1D2_1 +
 B1D1D2_1 +
 B2D1D2_1 +
 A1D1D3_1 +
 A2D1D3_1 +
```

```
 B1D1D3_1 +
 B2D1D3_1 +
 D2D1A1_1 +
 D3D1A1_1 +
 D2D1A2_1 +
 D3D1A2_1 +
 D2D1B1_1 +
 D3D1B1_1 +
 D2D1B2_1 +
 D3D1B2_1 +
 A1D1B1_1 +
 A2D1B1_1 +
 A1D1B2_1 +
 A2D1B2_1 +
 B1D1A1_1 +
 B2D1A1_1 +
 B1D1A2_1 +
 B2D1A2_1 +
 B2D1B1_1 +
 B1D1B2_1;

 ! Re-scaled r-centrality
rcD1_1s = (rcD1_1)/26;


 ! r-centrality  D2 Group 1

NEW(rcD2_1  rcD2_1s);
rcD2_1 = A1D2D1_1 +
 A2D2D1_1 +
 B1D2D1_1 +
 B2D2D1_1 +
 A1D2D3_1 +
 A2D2D3_1 +
 B1D2D3_1 +
 B2D2D3_1 +
 D1D2A1_1 +
 D3D2A1_1 +
 D1D2A2_1 +
 D3D2A2_1 +
 D1D2B1_1 +
 D3D2B1_1 +
 D1D2B2_1 +
 D3D2B2_1 +
 A1D2B1_1 +
 A2D2B1_1 +
 A1D2B2_1 +
 A2D2B2_1 +
 B1D2A1_1 +
```

```
 B2D2A1_1 +
 B1D2A2_1 +
 B2D2A2_1 +
 B2D2B1_1 +
 B1D2B2_1;

 ! Re-scaled r-centrality
rcD2_1s = (rcD2_1)/26;


 ! r-centrality  D3 Group 1

NEW(rcD3_1  rcD3_1s);
rcD3_1 = A1D3D1_1 +
 A2D3D1_1 +
 B1D3D1_1 +
 B2D3D1_1 +
 A1D3D2_1 +
 A2D3D2_1 +
 B1D3D2_1 +
 B2D3D2_1 +
 D1D3A1_1 +
 D2D3A1_1 +
 D1D3A2_1 +
 D2D3A2_1 +
 D1D3B1_1 +
 D2D3B1_1 +
 D1D3B2_1 +
 D2D3B2_1 +
 A1D3B1_1 +
 A2D3B1_1 +
 A1D3B2_1 +
 A2D3B2_1 +
 B1D3A1_1 +
 B2D3A1_1 +
 B1D3A2_1 +
 B2D3A2_1 +
 B2D3B1_1 +
 B1D3B2_1;

 ! Re-scaled r-centrality
rcD3_1s = (rcD3_1)/26;


 ! r-centrality  B1 Group 1

NEW(rcB1_1  rcB1_1s);
rcB1_1 = D1B1A1_1 +
 D2B1A1_1 +
```

```
 D3B1A1_1 +
 B2B1A1_1 +
 D1B1A2_1 +
 D2B1A2_1 +
 D3B1A2_1 +
 B2B1A2_1 +
 A1B1D1_1 +
 A2B1D1_1 +
 B2B1D1_1 +
 A1B1D2_1 +
 A2B1D2_1 +
 B2B1D2_1 +
 A1B1D3_1 +
 A2B1D3_1 +
 B2B1D3_1 +
 A1B1B2_1 +
 A2B1B2_1 +
 D1B1B2_1 +
 D2B1B2_1 +
 D3B1B2_1;

 ! Re-scaled r-centrality
rcB1_1s = (rcB1_1)/22;


 ! r-centrality  B2 Group 1

NEW(rcB2_1  rcB2_1s);
rcB2_1 = D1B2A1_1 +
 D2B2A1_1 +
 D3B2A1_1 +
 B1B2A1_1 +
 D1B2A2_1 +
 D2B2A2_1 +
 D3B2A2_1 +
 B1B2A2_1 +
 A1B2D1_1 +
 A2B2D1_1 +
 B1B2D1_1 +
 A1B2D2_1 +
 A2B2D2_1 +
 B1B2D2_1 +
 A1B2D3_1 +
 A2B2D3_1 +
 B1B2D3_1 +
 A1B2B1_1 +
 A2B2B1_1 +
 D1B2B1_1 +
 D2B2B1_1 +
```

```
 D3B2B1_1;

 ! Re-scaled r-centrality
rcB2_1s = (rcB2_1)/22;


 ! r-centrality  A1 Group 2

NEW(rcA1_2  rcA1_2s);
rcA1_2 = D1A1A2_2 +
 D2A1A2_2 +
 D3A1A2_2 +
 B1A1A2_2 +
 B2A1A2_2 +
 A2A1D1_2 +
 A2A1D2_2 +
 A2A1D3_2 +
 A2A1B1_2 +
 A2A1B2_2 +
 D1A1B1_2 +
 D2A1B1_2 +
 D3A1B1_2 +
 D1A1B2_2 +
 D2A1B2_2 +
 D3A1B2_2 +
 B1A1D1_2 +
 B2A1D1_2 +
 B1A1D2_2 +
 B2A1D2_2 +
 B1A1D3_2 +
 B2A1D3_2 +
 B2A1B1_2 +
 B1A1B2_2;

 ! Re-scaled r-centrality
rcA1_2s = (rcA1_2)/24;


 ! r-centrality  A2 Group 2

NEW(rcA2_2  rcA2_2s);
rcA2_2 = D1A2A1_2 +
 D2A2A1_2 +
 D3A2A1_2 +
 B1A2A1_2 +
 B2A2A1_2 +
 A1A2D1_2 +
 A1A2D2_2 +
 A1A2D3_2 +
```

```
 A1A2B1_2 +
 A1A2B2_2 +
 D1A2B1_2 +
 D2A2B1_2 +
 D3A2B1_2 +
 D1A2B2_2 +
 D2A2B2_2 +
 D3A2B2_2 +
 B1A2D1_2 +
 B2A2D1_2 +
 B1A2D2_2 +
 B2A2D2_2 +
 B1A2D3_2 +
 B2A2D3_2 +
 B2A2B1_2 +
 B1A2B2_2;

 ! Re-scaled r-centrality
rcA2_2s = (rcA2_2)/24;


 ! r-centrality  D1 Group 2

NEW(rcD1_2  rcD1_2s);
rcD1_2 = A1D1D2_2 +
 A2D1D2_2 +
 B1D1D2_2 +
 B2D1D2_2 +
 A1D1D3_2 +
 A2D1D3_2 +
 B1D1D3_2 +
 B2D1D3_2 +
 D2D1A1_2 +
 D3D1A1_2 +
 D2D1A2_2 +
 D3D1A2_2 +
 D2D1B1_2 +
 D3D1B1_2 +
 D2D1B2_2 +
 D3D1B2_2 +
 A1D1B1_2 +
 A2D1B1_2 +
 A1D1B2_2 +
 A2D1B2_2 +
 B1D1A1_2 +
 B2D1A1_2 +
 B1D1A2_2 +
 B2D1A2_2 +
 B2D1B1_2 +
```

```
 B1D1B2_2;

 ! Re-scaled r-centrality
rcD1_2s = (rcD1_2)/26;


 ! r-centrality  D2 Group 2

NEW(rcD2_2  rcD2_2s);
rcD2_2 = A1D2D1_2 +
 A2D2D1_2 +
 B1D2D1_2 +
 B2D2D1_2 +
 A1D2D3_2 +
 A2D2D3_2 +
 B1D2D3_2 +
 B2D2D3_2 +
 D1D2A1_2 +
 D3D2A1_2 +
 D1D2A2_2 +
 D3D2A2_2 +
 D1D2B1_2 +
 D3D2B1_2 +
 D1D2B2_2 +
 D3D2B2_2 +
 A1D2B1_2 +
 A2D2B1_2 +
 A1D2B2_2 +
 A2D2B2_2 +
 B1D2A1_2 +
 B2D2A1_2 +
 B1D2A2_2 +
 B2D2A2_2 +
 B2D2B1_2 +
 B1D2B2_2;

 ! Re-scaled r-centrality
rcD2_2s = (rcD2_2)/26;


 ! r-centrality  D3 Group 2

NEW(rcD3_2  rcD3_2s);
rcD3_2 = A1D3D1_2 +
 A2D3D1_2 +
 B1D3D1_2 +
 B2D3D1_2 +
 A1D3D2_2 +
 A2D3D2_2 +
```

```
 B1D3D2_2 +
 B2D3D2_2 +
 D1D3A1_2 +
 D2D3A1_2 +
 D1D3A2_2 +
 D2D3A2_2 +
 D1D3B1_2 +
 D2D3B1_2 +
 D1D3B2_2 +
 D2D3B2_2 +
 A1D3B1_2 +
 A2D3B1_2 +
 A1D3B2_2 +
 A2D3B2_2 +
 B1D3A1_2 +
 B2D3A1_2 +
 B1D3A2_2 +
 B2D3A2_2 +
 B2D3B1_2 +
 B1D3B2_2;

 ! Re-scaled r-centrality
rcD3_2s = (rcD3_2)/26;


 ! r-centrality  B1 Group 2

NEW(rcB1_2  rcB1_2s);
rcB1_2 = D1B1A1_2 +
 D2B1A1_2 +
 D3B1A1_2 +
 B2B1A1_2 +
 D1B1A2_2 +
 D2B1A2_2 +
 D3B1A2_2 +
 B2B1A2_2 +
 A1B1D1_2 +
 A2B1D1_2 +
 B2B1D1_2 +
 A1B1D2_2 +
 A2B1D2_2 +
 B2B1D2_2 +
 A1B1D3_2 +
 A2B1D3_2 +
 B2B1D3_2 +
 A1B1B2_2 +
 A2B1B2_2 +
 D1B1B2_2 +
 D2B1B2_2 +
```

```
 D3B1B2_2;

 ! Re-scaled r-centrality
rcB1_2s = (rcB1_2)/22;


 ! r-centrality  B2 Group 2

NEW(rcB2_2  rcB2_2s);
rcB2_2 = D1B2A1_2 +
 D2B2A1_2 +
 D3B2A1_2 +
 B1B2A1_2 +
 D1B2A2_2 +
 D2B2A2_2 +
 D3B2A2_2 +
 B1B2A2_2 +
 A1B2D1_2 +
 A2B2D1_2 +
 B1B2D1_2 +
 A1B2D2_2 +
 A2B2D2_2 +
 B1B2D2_2 +
 A1B2D3_2 +
 A2B2D3_2 +
 B1B2D3_2 +
 A1B2B1_2 +
 A2B2B1_2 +
 D1B2B1_2 +
 D2B2B1_2 +
 D3B2B1_2;

 ! Re-scaled r-centrality
rcB2_2s = (rcB2_2)/22;

! New measures 12 June - Test diff r centrality
! Test for group 0 (comorbid)
! D3 (Down - all other bridge effects)
NEW(dfDA1_0);
dfDA1_0 = rcD3_0s - rcA1_0s;
NEW(dfDA2_0);
dfDA2_0 = rcD3_0s - rcA2_0s;
NEW(dfDB1_0);
dfDB1_0 = rcD3_0s - rcB1_0s;
NEW(dfDB2_0);
dfDB2_0 = rcD3_0s - rcB2_0s;
NEW(dfDD1_0);
dfDD1_0 = rcD3_0s - rcD1_0s;
NEW(dfDD2_0);
```

```
dfDD2_0 = rcD3_0s - rcD2_0s;

! Test for group 1 (depression) not cheerful(d1) - all other effects;
NEW(dfDA1_1);
dfDA1_1 = rcD1_1s - rcA1_1s;
NEW(dfDA2_1);
dfDA2_1 = rcD1_1s - rcA2_1s;
NEW(dfDB1_1);
dfDB1_1 = rcD1_1s - rcB1_1s;
NEW(dfDB2_1);
dfDB2_1 = rcD1_1s - rcB2_1s;
NEW(dfDD2_1);
dfDD2_1 = rcD1_1s - rcD2_1s;
NEW(dfDD3_1);
dfDD3_1 = rcD1_1s - rcD3_1s;


! Test for group 2 (anxiety) Down (d3) - all other effects;
NEW(dfDA1_2);
dfDA1_2 = rcD3_2s - rcA1_2s;
NEW(dfDA2_2);
dfDA2_2 = rcD3_2s - rcA2_2s;
NEW(dfDB1_2);
dfDB1_2 = rcD3_2s - rcB1_2s;
NEW(dfDB2_2);
dfDB2_2 = rcD3_2s - rcB2_2s;
NEW(dfDD1_2);
dfDD1_2 = rcD3_2s - rcD1_2s;
NEW(dfDD2_2);
dfDD2_2 = rcD3_2s - rcD2_2s;




OUTPUT:         TECH1 TECH8 STDYX cinterval patterns;
!SAVEDATA:
!PARAMETERS = bpars_saved.dat;
!FILE IS fscores_saved.dat;
!AVE IS FSCORES(30,10);
```
